# Phyto‐Confined CeO_x_ with Synergistic Lattice Distortion and Oxygen Vacancies Drives Efficient Urea Electrosynthesis

**DOI:** 10.1002/advs.202513799

**Published:** 2025-11-04

**Authors:** Ziming Zhao, Yaru Wei, Haoyu Duan, Yuhan Mei, Huan Li

**Affiliations:** ^1^ Chongqing Institute of Green and Intelligent Technology Chinese Academy of Sciences Chongqing 400714 P. R. China; ^2^ Chongqing School University of Chinese Academy of Sciences Chongqing 400714 P. R. China

**Keywords:** C‐N coupling, lattice‐distorted CeO_x_, oxygen vacancies, phyto‐hyperaccumulation confinement, synergistic effects

## Abstract

Electrocatalytic urea synthesis from carbon dioxide and nitrates is hindered by sluggish multi‐electron kinetics and unclear C‐N coupling mechanism. Herein, lattice‐distorted CeO_x_ nanoparticles are introduced with abundant oxygen vacancies (O_V_), confined within a porous carbon framework (*d*‐CeO_x_/PC), fabricated via a phyto‐hyperaccumulation confinement strategy. Precise structural modulation induces ultrasmall (≈2 nm), uniformly dispersed, contracted Ce─O bonds (≈2.29 Å) and creates a highly active environment for urea electrosynthesis. In situ ATR‐FTIR spectroscopy identifies key intermediates, confirming C‐N coupling pathway. Theoretical calculation reveals contracted bonds strengthen Ce 4f‐O 2p orbital hybridization, restricting lattice oxygen (O_L_) migration and slowing O_V_ diffusion/annihilation. Simultaneously, bond contraction induces localized electron redistribution around O_V_. These O_V_ promote mixed Ce^3+^/Ce^4+^ valence, while the highly covalent, contracted Ce─O bonds stabilize Ce^3+^, forming localized “electron reservoirs” for flexible multi‐step electron transfer. These synergistic effects enhance reactant (CO_2_/NO_3_^−^) adsorption, stabilize key intermediates (*CO/*NO), and drastically lower the C‐N coupling energy barrier (*NO+*CO→*OCNO, 0.18 eV), while suppressing competing hydrogenation pathways to byproducts. The porous carbon framework further improves durability (>100 h) and active site accessibility. This reduction in C‐N barrier, identified as the key kinetic descriptor enabled by structural modulation, provides mechanistic insight for designing catalysts for sustainable urea production from waste.

## Graphical abstract

Lattice‐distorted CeO_x_ nanoparticles with abundant oxygen vacancies, confined in porous carbon (*d*‐CeO_x_/PC), enable efficient electrocatalytic urea synthesis from CO_2_ and NO_3_^−^. Contracted Ce─O bonds (≈2.29 Å) stabilize O_V_, creating electron reservoirs for flexible multi‐electron transfer. This synergistically enhances reactant adsorption, stabilizes intermediates, and drastically lowers the C‐N coupling barrier (*NO+*CO→*OCNO, 0.18 eV), enabling selective urea synthesis.

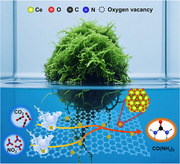

## 1 Introduction

Urea (CO(NH_2_)_2_), a cornerstone of modern agriculture and chemical industry, serves as a crucial nitrogen fertilizer and nitrogenous chemical intermediate, and its global annual production exceeds 100 million tons.^[^ ^1^, ^2^ ^]^ The current industrial production, however, relies almost exclusively on the century‐old Bosch‐Haber process followed by the energy‐intensive urea synthesis (CO_2_+2NH_3_→ CO(NH_2_)_2_ + H_2_O). This route demands high temperatures (400–600 °C) and pressures (10–20 MPa), consuming ≈2% of global energy and releasing ≈300 million tons of CO_2_ annually.^[^ ^3^, ^4^ ^]^ Consequently, the reliance on fossil fuels and associated environmental burdens motivate the need for a sustainable alternative to current approaches.

Electrocatalytic C‐N coupling with co‐reduction of CO_2_ and nitrate (NO_3_^−^) for urea synthesis emerges as eco‐friendly and cost‐effective solution, offering ambient‐temperature operation.^[^ ^5^, ^6^, ^7^, ^8^, ^9^, ^10^, ^11^, ^12^ ^]^ Meanwhile, superior to the independent conversion of nitrate pollutants and CO_2_, the coupling of the both is employed to synthesize high‐value organonitrogen compounds.^[^ ^13^ ^]^ This approach bypasses ammonia intermediates, directly coupling C‐N through multiple electrons transfer and protons‐coupling to form urea. Nevertheless, electrocatalytic urea synthesis faces formidable challenges arising from its intricate reaction pathways, which encompass both multistep electrochemical steps (especially, C‐N coupling).^[^ ^14^, ^15^ ^]^ The preferential hydrogenation of key intermediates (e.g., *CO, *NO, *NH) into byproducts such as ammonia, formate rather than facilitating C‐N coupling severely restricts the efficiency of urea production.^[^ ^16^ ^]^ Therefore, a deep understanding of the complex 16‐electron transfer co‐reduction mechanism and the rational design of highly active and selective electrocatalyst for C─N bond formation is a substantial scientific challenge.

Recent advancements, defect engineering in metal oxides has been investigated as a viable strategy to achieve efficient electrocatalytic C‐N coupling: abundant oxygen vacancies (O_V_) facilitate and stabilize key intermediates, enhancing the probability of C─N bond formation and selectivity^[^ ^17^, ^18^, ^19^, ^20^, ^21^, ^22^, ^23^, ^24^ ^]^; Especially for CeO_x_, O_V_ not only facilitate the insertion of nitro‐oxygen/carbon‐oxygen intermediates into these vacancies, thereby promoting coupling probability (over protonation), but also optimize the Ce^3+^/Ce^4+^ ratio and enhance electron localization, which improve redox kinetics.^[^ ^25^, ^26^, ^27^ ^]^ Additionally, decreasing the CeO_x_ crystallite size can further modulate its electronic structure. This modulation can increase the Ce^3+^ proportion and the number of O_V_, as well as enhance the utilization and dispersion of active sites. As a result, the electrocatalytic activity is significantly improved, highlighting the critical role of crystallite size in optimizing CeO_x_ based catalysts.^[^ ^28^, ^29^, ^30^, ^31^ ^]^ Despite its promise, small‐sized CeO_x_ still faces critical challenges. First, small‐sized CeO_x_ with high surface energy are inherently prone to migration and aggregation during synthesis and electrocatalysis, which reduces the active surface area and undermines the stability of the electrocatalyst.^[^ ^32^, ^33^, ^34^, ^35^ ^]^ Second, insufficient conductivity limits efficient electron transport on the electrocatalyst surface, particularly at high current densities.^[^ ^36^ ^]^ Third, under applied potentials, lattice oxygen atoms (O_L_) may migrate to fill the adjacent O_V_ during prolonged electrocatalysis, reducing the Ce^3^⁺/Ce⁴⁺ ratio and consequently decreasing the number of active sites.^[^ ^37^, ^38^ ^]^

To address these challenges, in the present work, small‐sized CeO_x_ with O_V_ and lattice distortion supported on porous framework carbon (denoted as PC) via phyto‐hyperaccumulation confinement is synthesized (namely *d*‐CeO_x_/PC), serving as an electrocatalyst for the coupling of CO_2_ and NO_3_^−^ toward urea synthesis. In the process, living *Sphagnum* moss was selected as the biological template owing to its exceptional hyperaccumulation capacity for metal ions and its unique hierarchical sponge‐like structure.^[^ ^39^ ^]^ This structure, rich in oxygen‐containing functional groups (e.g., hydroxyl, carboxyl), facilitates chelation and uniform enrichment of metal ions within its micro‐/mesoporous network.^[^ ^40^ ^]^ By leveraging this hyperaccumulation capacity, a uniform dispersion and enrichment of Ce and Zn ions were achieved. During freeze‐drying, the intracellular fluid migration driven by osmotic pressure further promotes the highly dispersed attachment of Ce and Zn ions onto the cell walls. Subsequent calcination at 800 °C under Ar atmosphere enabled biomass template removal, followed by selective Zn^2+^ leaching via alkaline etching. A final annealing step under Ar/H_2_ atmosphere yielded a porous framework carbon embedding small‐sized CeO_x_ nanoparticles. Owing to the confinement effect of biochar and selective removal of Zn^2+^, this approach obtains small‐sized CeO_x_ nanoparticles (≈2 nm) with distorted lattice parameters (Ce─O bond length shortened from 2.31 Å to 2.29 Å), decreasing the difficulty in precisely controlling small‐sized dispersion of CeO_x_ species in conventional supported synthesis. The compressed lattice enhances Ce─O bond strength, stabilizing O_V_ and promoting electron transfer. Moreover, the high specific surface area of the porous carbon framework improves electrical conductivity and maximizes active site exposure. The *d*‐CeO_x_/PC demonstrated excellent electrocatalytic performance in the synthesis of urea from CO_2_ and NO_3_^−^ under ambient conditions, attaining a remarkable yield of 532.13 mg h^−1^ g_cat._^−1^ and a Faradaic efficiency (FE) of 35.51% at a potential of −1.5 V versus RHE. This design synergistically enhances the electrocatalytic activity, stability, and selectivity of CeO_2_‐based materials for urea synthesis.

## 2 Results and Discussion

### 2.1 Morphology and Structural Characterization

**Figure 1:**
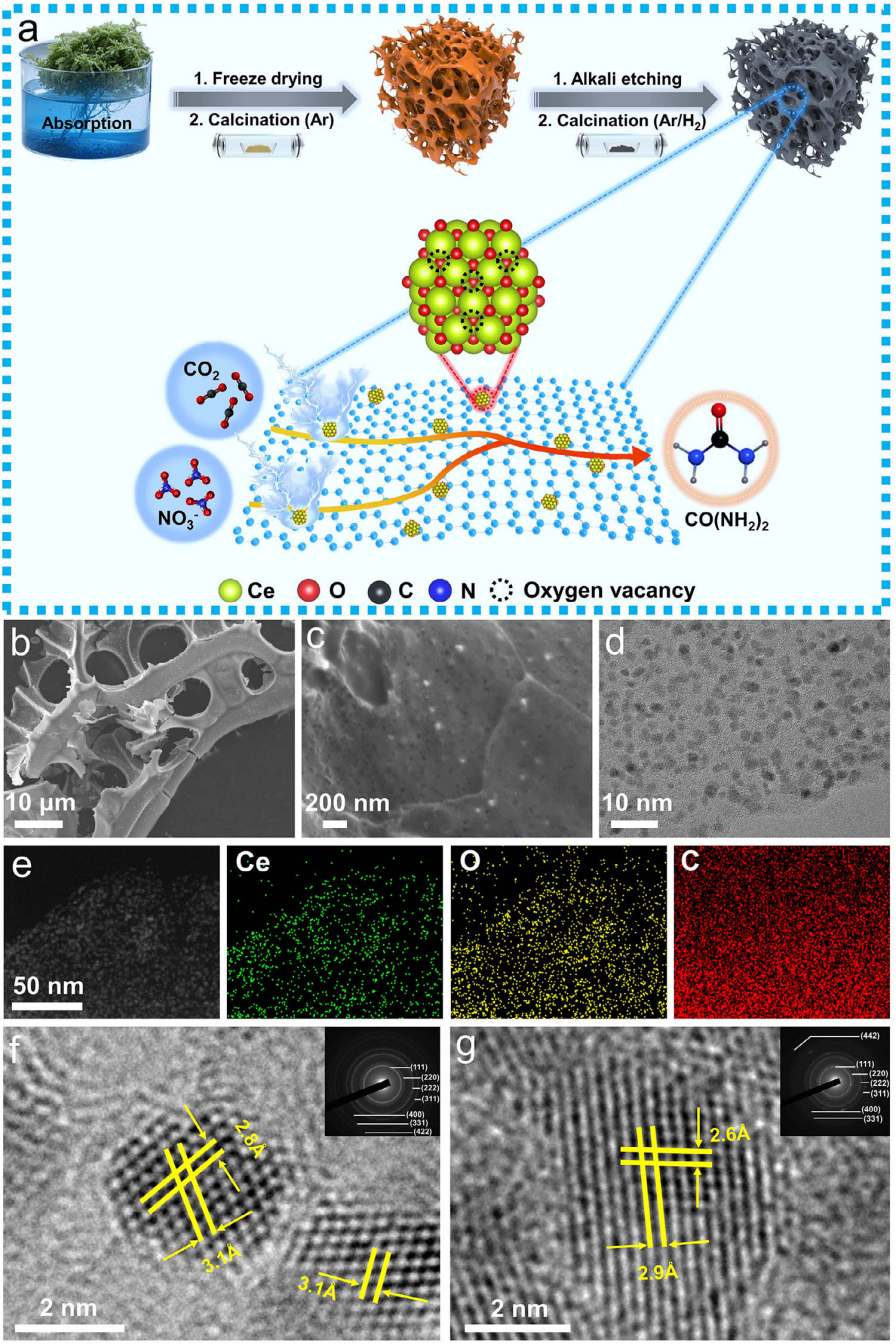
a) Schematic illustration for the fabrication procedure of *d*‐CeO_x_/PC; b,c) SEM images, d) TEM and e) Elemental mapping images of *d*‐CeO_x_/PC; f) HRTEM image of CeO_2_/PC, with the inset displaying the SAED pattern of CeO_2_/PC; g) HRTEM image of *d*‐CeO_x_/PC, with the inset displaying the SAED pattern of *d*‐CeO_x_/PC.

The synthetic procedure of the *d*‐CeO_x_/PC is illustrated in **Figure** **1**a. Scanning electron microscopy (SEM) images of the *d*‐CeO_x_/PC reveal that the *Sphagnum* moss‐derived carbon support maintains its hierarchically layered and highly interconnected three‐dimensional architecture, featuring well‐defined porous structures distributed uniformly across the surface (Figure 1b,c; Figure S1a,b, Supporting Information). The intricate 3D framework and pore structure not only provide structural robustness but also facilitates efficient mass transport pathways and electrical conductivity critical for electrocatalytic processes. Transmission electron microscopy (TEM) and energy dispersive X‐ray (EDX) elemental mapping of the *d*‐CeO_x_/PC demonstrate that small‐sized *d*‐CeO_x_ nanoparticles are uniformly dispersed on the PC support, accompanied by homogeneous distribution of Ce, O, and C elements (Figure 1d,e). A comparison of particle sizes across different samples reveals that CeO_2_/CeO_x_ nanoparticles in CeO_2_/PC and CeO_x_/PC exhibit slightly larger dimensions (Figure S2a,b, Supporting Information), whereas *d*‐CeO_2_ nanoparticles in *d*‐CeO_2_/PC show sizes comparable to those of *d*‐CeO_x_ (Figure S2c, Supporting Information). These observations are further supported by particle size distribution statistics: *d*‐CeO_x_ nanoparticles in *d*‐CeO_x_/PC span a diameter range of 1.5‐4 nm, with the average particle sizes of 2.49 ± 0.47 nm uniformly dispersed on the PC support (Figure S3a, Supporting Information); similarly, *d*‐CeO_2_ nanoparticles in *d*‐CeO_2_/PC display a comparable size distribution (average particle sizes of 3.03 ± 0.77 nm), matching that of *d*‐CeO_x_ (Figure S3b, Supporting Information). In contrast, CeO_x_/CeO_2_ nanoparticles in CeO_x_/PC (Figure S3c, Supporting Information) and CeO_2_/PC (Figure S3d, Supporting Information) exhibit larger average sizes of 4.11 ± 0.69 nm and 4.22 ± 0.96 nm, respectively. This reduced particle size and improved dispersion are attributed to the deliberate lattice compression introduced in both *d*‐CeO_x_ and *d*‐CeO_2_ after Zn etching, which facilitates the formation of smaller, more well‐dispersed crystallites. Additionally, pure CeO_2_ nanoparticles synthesized without the plant‐mediated transport mechanism exhibit diameters exceeding 10 nm with size inhomogeneity and agglomeration (Figure S2d, Supporting Information), highlighting the biotemplate's role in confining nanoparticle growth. Notably, high‐resolution TEM (HRTEM) image of the CeO_2_/PC displays lattice spacing of 2.8 and 3.1 Å (Figure 1f), corresponding to the (200) and (111) planes of cubic fluorite CeO_2_, respectively.^[^ ^41^ ^]^ In contrast, the (200) and (111) lattice spacing of *d*‐CeO_x_ with lattice distortion and O_V_ contract to 2.6 Å and 2.9 Å, respectively, accompanied by altered interplanar angles (Figure 1g). The insets in Figure 1f,g display the selected area electron diffraction (SAED) patterns, where isolated diffraction spots overlapped with concentric ring structures. The spacing values of these spot rings correspond to the various crystal planes of the cubic fluorite phase of CeO_2_, thus verifying the polycrystalline feature of *d*‐CeO_x_/PC. Collectively, these results confirm that *d*‐CeO_x_ exhibits lattice distortion, uniform nanoscale dimensions, and excellent dispersibility.

**Figure 2:**
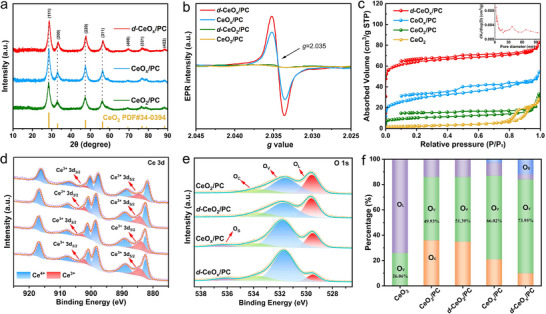
a) XRD spectra of CeO_2_/PC, CeO_x_/PC and *d*‐CeO_x_/PC; b) EPR spectra of CeO_2_/PC, *d*‐CeO_2_/PC, CeO_x_/PC and *d*‐CeO_x_/PC; c) Nitrogen adsorption‐desorption isotherm of pure CeO_2_, CeO_2_/PC, CeO_x_/PC and *d*‐CeO_x_/PC, with the inset showing the BJH pore size distribution of *d*‐CeO_x_/PC; d) High‐resolution XPS spectra of Ce 3d and e) O1s of CeO_2_/PC, *d*‐CeO_2_/PC, CeO_x_/PC and *d*‐CeO_x_/PC in sequence; f) The corresponding proportions of O_L_, O_V_, O_C_ and O_S_ in pure CeO_2_, CeO_2_/PC, *d*‐CeO_2_/PC, CeO_x_/PC and *d*‐CeO_x_/PC.

The crystal structures of *d*‐CeO_x_/PC and control samples were characterized by X‐ray diffraction (XRD). As shown in **Figure** **2**a, diffraction peaks at 28.5°, 33.0°, 47.4°, 56.3°, 59.0°, 69.4°, 76.6°, and 88.4° correspond to the (111), (200), (220), (311), (400), (331), and (422) crystal planes of the cubic fluorite structure of CeO_2_, consistent with the data in JCPDS card No. 34–0394. No significant peak shift was observed for CeO_2_ formed on PC or CeO_x_/PC derived from Ar/H_2_ heat treatment. However, for *d*‐CeO_2_/PC and the subsequent *d*‐CeO_x_/PC with Ar/H_2_ treatment, lattice distortion and the generation of O_V_ caused their diffraction peaks to exhibit an overall rightward shift and weakening (Figure 2a; Figure S4, Supporting Information). According to refinement of the XRD patterns (Figure S5, Supporting Information), the unit cell parameters of all samples were determined and summarized in Table S1 (Supporting Information). Results indicate that, compared to the theoretical lattice parameters of pristine cubic CeO_2_ (a = b = c ≈ 5.41 Å), both *d*‐CeO_x_/PC and *d*‐CeO_2_/PC exhibit reduced lattice parameters (a, b, c), accompanied by a deviation from ideal cubic symmetry (a ≠ b = c). The unit cell volume for these two composites is calculated as 144.15 Å^3^, significantly smaller than the theoretical value (158.38 Å^3^). In contrast, CeO_x_/PC and CeO_2_/PC show only minor deviations from the theoretical lattice parameters. These observations confirm the existence of compressive lattice distortion in both *d*‐CeO_x_/PC and *d*‐CeO_2_/PC. Additionally, combined XRD and TEM analyses revealed that the carbon support in *d*‐CeO_x_/PC exhibited an amorphous structure.

Electron paramagnetic resonance (EPR) spectroscopy is an effective method for detecting vacancies (O_V_). As shown in Figure 2b, all samples (CeO_2_/PC, *d*‐CeO_2_/PC, CeO_x_/PC, and *d*‐CeO_x_/PC) exhibit defect signals at g = 2.035, corresponding to O_V_, with distinct EPR intensities.^[^ ^42^, ^43^ ^]^ The peak intensity order indicates that *d*‐CeO_x_/PC possesses the highest O_V_ concentration. Abundant O_V_ induce charge redistribution within the Ce‐O lattice, promoting the reduction of Ce^4+^ to Ce^3+^ and forming highly localized electron states as electron storage sites and active centers, which regulate band structures and conductivity, thereby lowering reaction energy barriers and enhancing catalytic efficiency. Brunauer‐Emmett‐Teller (BET) measurements were conducted to characterize the specific surface area and pore size distribution of the samples (Figure 2c). The *d*‐CeO_x_/PC exhibits a typical Type I isotherm, with a BET specific surface area of 245.99 m^2^ g^−1^, which is significantly higher than those of pure CeO_2_ (8.69 m^2^ g^−1^), CeO_2_/PC (43.26 m^2^ g^−1^), and CeO_x_/PC (100.93 m^2^ g^−1^). Its pore size distribution is dominated by micropores and mesopores, as shown in the inset of Figure 2c. Notably, the micropores originate from the small stomata inherently present in plant tissues, and BJH analysis of CeO_x_/PC, CeO_2_/PC (Figure S6a,b, Supporting Information) and *d*‐CeO_x_/PC revealed that the microporous structure remained intact across all samples, indicating that the calcination process largely did not cause structural collapse. Meanwhile, the formation of vacancies promotes mesopore generation: CeO_x_/PC shows a higher mesopore proportion than CeO_2_/PC, and *d*‐CeO_x_/PC further increases the mesopore fraction due to concurrent O_V_ and lattice distortion. The enhanced mesoporosity facilitates reactant mass transfer during electrocatalysis.

X‐ray photoelectron spectroscopy (XPS) was performed to investigate the surface chemical states and elemental compositions of *d*‐CeO_x_/PC and control samples. The XPS survey spectra of all samples confirmed the presence of Ce, O, and C elements with binding energies at 883.2 eV, 529.7 eV, and 284.8 eV, respectively (Figure S7, Supporting Information). As shown in Figure 2d, the high‐resolution Ce 3d spectrum reveals characteristic peaks indicative of both Ce^4+^ and Ce^3+^ states. Peaks at 900.8, 907.6, and 916.7 eV correspond to Ce^4+^ final states (3d^9^4f^0^, 3d^9^4f^2^, and 3d^9^4f^1^, respectively), with their spin‐orbit split counterparts observed at 881.9, 888.9, and 897.9 eV. Additionally, the peaks located at 884.3 and 902.3 eV are assigned to the Ce^3+^ 3d_5/2_ (final state 3d^9^4f^1^) and 3d_3/2_ (final state 3d^9^4f^2^) states.^[^ ^44^, ^45^, ^46^ ^]^ The area ratio of Ce^3+^ characteristic peaks correlates closely with O_V_ content. Compared to CeO_2_/PC and *d*‐CeO_2_/PC, CeO_x_/PC and *d*‐CeO_x_/PC show significantly enhanced Ce^3+^ 3d_5/2_ and 3d_3/2_ peak intensities, while pure CeO_2_ exhibits the lowest Ce^3+^ intensity among all samples (Figure S8a, Supporting Information), indicating higher Ce^3+^ and O_V_ contents in CeO_x_/PC and *d*‐CeO_x_/PC. For the O 1s high‐resolution spectrum (Figure 2e), peaks for lattice oxygen (O_L_), oxygen vacancies (O_V_), chemisorbed oxygen (O_C_), and surface oxygen species (O_S_) appear at 529.4, 531.6, 533.6, and 536.1 eV, respectively.^[^ ^47^, ^48^ ^]^ Quantitative analysis of peak areas (Figure 2f) reveals O_V_ proportions of 73.95% for *d*‐CeO_x_/PC and 66.02% for CeO_x_/PC, significantly higher than *d*‐CeO_2_/PC (51.39%) and CeO_2_/PC (49.93%), indicating that *d*‐CeO_x_/PC has more O_V_, in agreement with the XPS results of Ce 3d. The increased O_V_ concentration may promote the formation of O_S_ species. In contrast, pure CeO_2_ shows the lowest O_V_ content (26.06%) and no O_C_ and O_S_ peaks (Figure S8b, Supporting Information). In the C 1s high‐resolution spectra (Figure S9a,b, Supporting Information), the peak at 283.8 eV corresponds to C─C bonds in PC, while peaks at 284.8 and 288.7 eV are attributed to external carbon standards.

**Figure 3:**
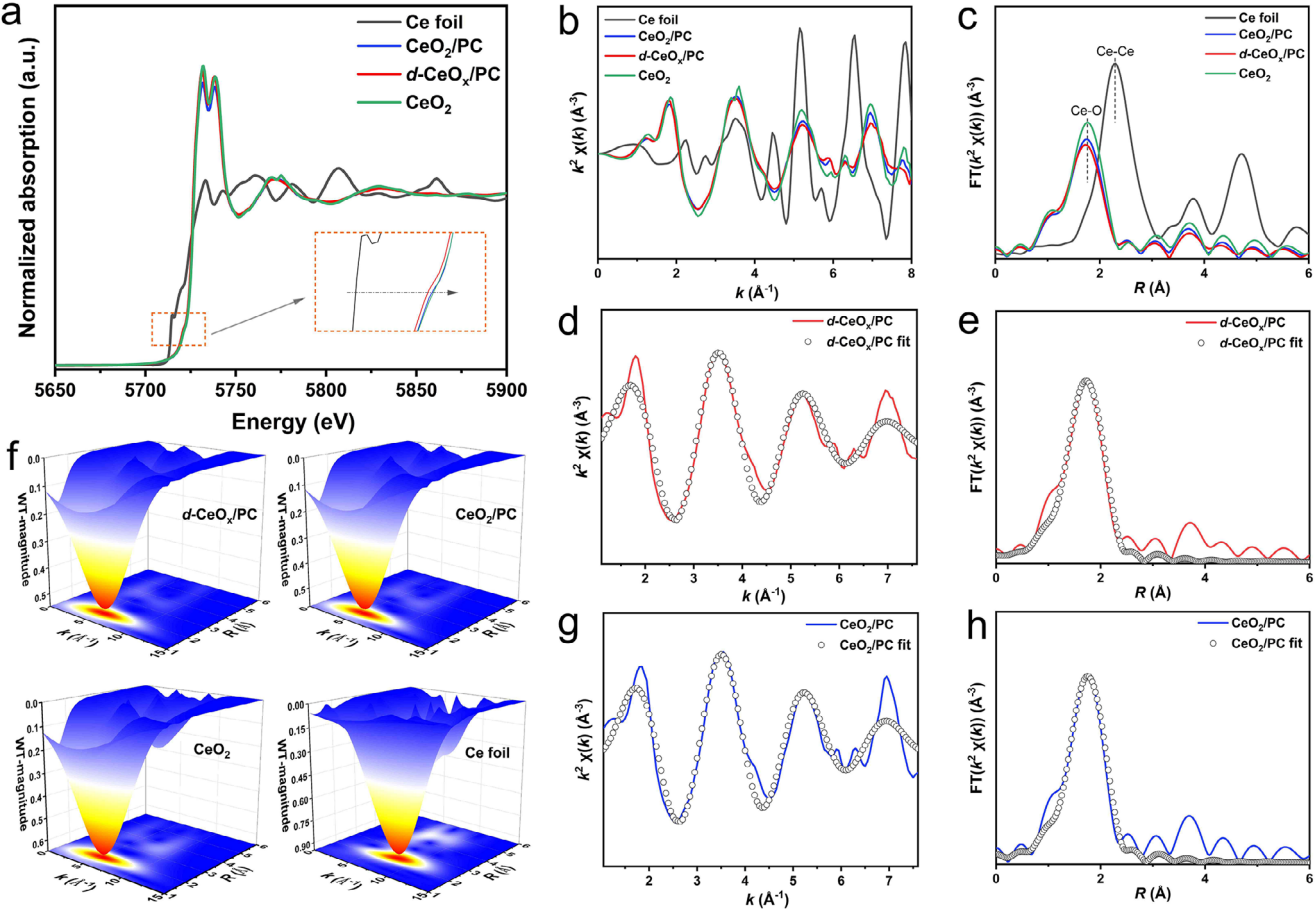
a) Normalized Ce L_3_‐edge XANES and b) EXAFS spectra in K‐space for Ce foil, pure CeO_2_, CeO_2_/PC and *d*‐CeO_x_/PC; c) Fourier transforms of EXAFS spectra in R‐space for Ce foil, pure CeO_2_, CeO_2_/PC and *d*‐CeO_x_/PC; Fitting profiles of K‐space EXAFS spectra for d) *d*‐CeO_x_/PC and g) CeO_2_/PC; Fitting profiles of R‐space EXAFS spectra for e) *d*‐CeO_x_/PC and h) CeO_2_/PC; Wavelet transform patterns of Ce L_3_‐edge EXAFS spectra for f) Ce foil, pure CeO_2_, CeO_2_/PC and *d*‐CeO_x_/PC.

X‐ray absorption fine structure (XAFS) spectroscopy was employed to further investigate the coordination environment of Ce atoms, Ce─O bond lengths, and electronic structure changes in CeO_2_ on PC before and after lattice distortion and O_V_ formation. As shown in **Figure** **3**a, the X‐ray absorption near‐edge structure (XANES) at the Ce L_3_‐edge indicates that the peak position of CeO_2_/PC is close to that of pure CeO_2_, while the peak position of *d*‐CeO_x_/PC is more distant. However, both peak positions of *d*‐CeO_x_/PC and CeO_2_/PC are located between metallic Ce foil and pure CeO_2_, indicating that the valence state of Ce in them is between 0 and +4. Notably, *d*‐CeO_x_/PC exhibits a higher Ce^3+^ content, which is consistent with the XPS results. The K‐space EXAFS spectra (Figure 3b) and their respective fitting profiles (Figure 3d,g) for the samples reveal significant differences in the Ce L_3_ edge oscillation curves between *d*‐CeO_x_/PC and CeO_2_/PC, suggesting distinct coordination modes of Ce atoms. According to the fitting parameters, the coordination number of Ce in *d*‐CeO_x_/PC is calculated to be 7.52, lower than that in CeO_2_/PC (8.02), illustrating a decrease in Ce coordination number due to O_V_ formation in *d*‐CeO_x_. This difference (Δ coordination number = 8.02 – 7.52 = 0.5) indicates that each Ce atom in *d*‐CeO_x_ has ≈0.5 fewer oxygen neighbors. The corresponding bulk O_V_ concentration is quantified as ≈6.25% per Ce site. The lower bulk O_V_ concentration (from EXAFS) relative to the higher surface O_V_ concentration (from XPS) is reasonable, given that defect concentrations are typically far higher at the surface than in the bulk. The Fourier transform k^2^‐weighted extended X‐ray absorption fine structure (FT‐EXAFS) spectra in Figure 3c show a main peak at 1.7 Å attributed to the first shell of Ce‐O coordination.^[^ ^49^ ^]^ Compared to pure CeO_2_ and CeO_2_/PC, this main peak in *d*‐CeO_x_/PC becomes weakened and shifted to a shorter distance, indicating a contracted Ce─O bond in *d*‐CeO_x_. The coordination environment of CeO_2_/PC and *d*‐CeO_x_/PC was decoded through EXAFS spectrum fitting in R‐space (Figure 3e,h), with the refined parameters listed in Table S2 (Supporting Information). To further confirm the structure of *d*‐CeO_x_/PC, wavelet transform (WT) analysis was performed, which provides high‐resolution characterization in both K and R spaces. As shown in Figure 3f, all WT plots exhibit only one intensity maximum at ≈5.0 Å^−1^, assigned to the backscattering contribution of the first‐shell Ce‐O coordination. Compared with metallic Ce foil, no additional signal is detected near the higher‐shell peaks, indicating the absence of metallic Ce dispersion on PC.

### 2.2 Electrocatalytic Urea Production

**Figure 4:**
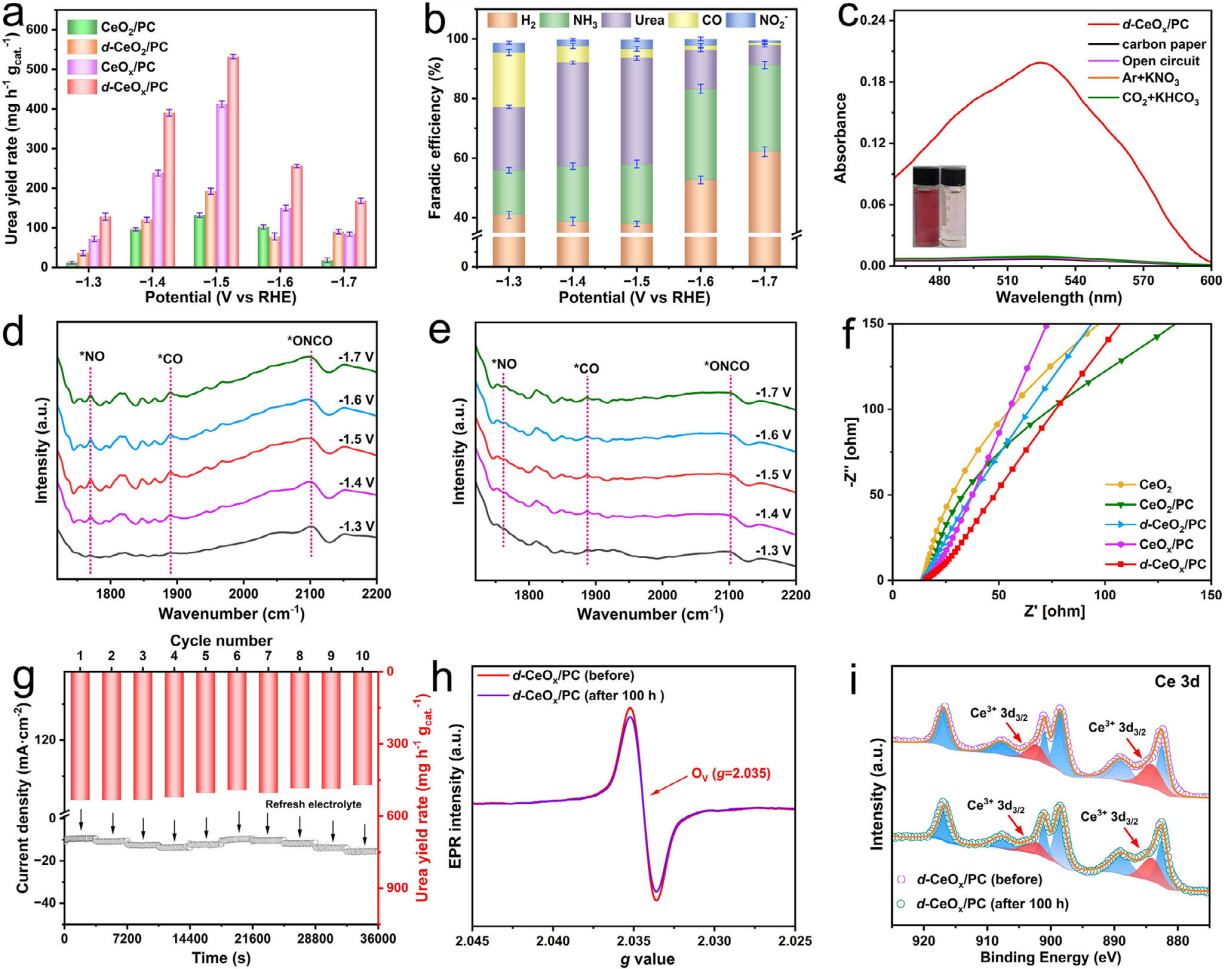
Urea yield rates of *d*‐CeO_x_/PC, CeO_x_/PC, *d*‐CeO_2_/PC and CeO_2_/PC at a series of potentials ranging from −1.3 to −1.7 V versus RHE; b) Faradaic efficiencies for all products at a range of applied potentials; c) UV–vis absorption spectra of electrolytes for *d*‐CeO_x_/PC under varied reaction conditions; In situ ATR‐FTIR spectroscopy of intermediate species on d) *d*‐CeO_x_/PC and e) CeO_2_/PC; f) Nyquist plots of EIS for all samples; g) Cycling performance of *d*‐CeO_x_/PC at −1.5 V versus RHE; h) EPR spectra and i) Ce 3d XPS spectra of *d*‐CeO_x_/PC before and after the stability test of 100 h.

To elucidate the electrocatalytic activity and selectivity of catalysts for CO_2_ and NO_3_^−^ conversion to urea, systematic performance in the co‐reduction reaction was evaluated using an H‐type cell under controlled potentials. The cathodic compartment contained 0.1 M KNO_3_ electrolyte, with continuous high‐purity CO_2_ bubbling at 30 mL min^−1^ during potentiostatic electrolysis. Liquid urea products generated within the electrolyte were quantified by ultraviolet spectrophotometry following a colorimetric reaction with diacetyl monoxime, with the corresponding calibration curve provided in Figure S10 (Supporting Information). Chronoamperometry was employed to investigate the electrocatalytic urea synthesis performance of *d*‐CeO_x_/PC, CeO_x_/PC, *d*‐CeO_2_/PC, CeO_2_/PC, and pure CeO_2_ under applied potentials ranging from −1.3 to −1.7 V versus RHE (Figures S11 and S12, Supporting Information). The results show that *d*‐CeO_x_/PC exhibits the highest urea yield rate across the entire potential range compared to control samples, as represented in **Figure** **4**a. Specifically at the potential of −1.5 V versus RHE, *d*‐CeO_x_/PC exhibits the maximum ultraviolet response, where the urea yield reaches the highest value of 532.13 ± 5.26 mg h^−1^ g_cat._^−1^ with a corresponding FE of 35.51 ± 3.62% (five repeated experiments) (Figures S13 and S14, Supporting Information), which is nearly ten times that of pure CeO_2_ (Figure S15, Supporting Information).

**Figure 5:**
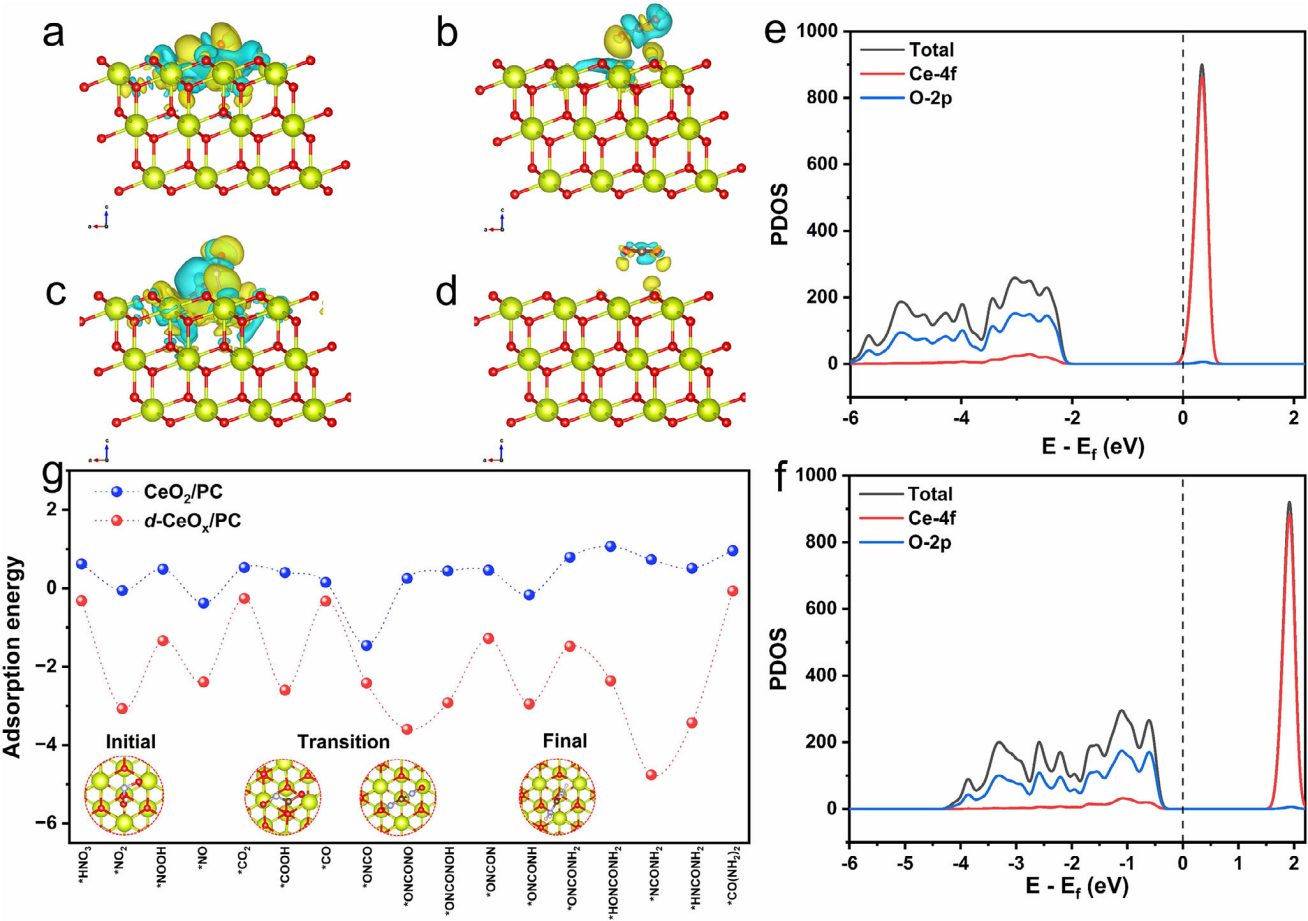
Difference charge density of *d*‐CeO_x_/PC during a) *NO and b) CO_2_ adsorption; Difference charge density of CeO_2_/PC during c) *NO and d) CO_2_ adsorption; e,f) PDOS for *d*‐CeO_x_/PC and CeO_2_/PC upon their respective adsorption processes; g) Variation in adsorption energies of key intermediates throughout the overall reaction over *d*‐CeO_x_/PC and CeO_2_/PC.

To further quantify the intrinsic activity per active site, turnover frequency (TOF) was calculated by normalizing the urea formation rate to the number of active sites (see Supporting Information for details). The *d*‐CeO_x_/PC exhibits the highest TOF value at −1.5 V versus RHE, significantly surpassing other catalysts, which indicates its superior intrinsic activity. It is noteworthy that CeO_x_/PC exhibits the second‐highest electrocatalytic activity among all control samples, thereby indicating a significant correlation between urea production efficiency and O_V_ concentration. Gas chromatography (GC) analysis identifies molecular hydrogen (H_2_) and carbon monoxide (CO) as predominant gaseous co‐products. The observed low FEs for H_2_ generation within the −1.3 to −1.5 V versus RHE potential range signify suppressed hydrogen evolution reaction (HER) selectivity for *d*‐CeO_x_/PC at lower negative potentials (Figure 4b). Ammonia (NH_3_) production from the singular nitrate reduction reaction (NO_3_RR) and co‐electrocatalytic processes was quantitatively measured ultraviolet spectrophotometrically via the indophenol blue method, with ammonium ion (NH_4_^+^) quantification standard curve illustrated in Figure S16 (Supporting Information). The *d*‐CeO_x_/PC exhibits higher NH_3_ yield rates and FEs for NO_3_RR alone than those in the co‐electrolysis process at −1.3 to −1.7 V versus RHE (Figure S17, Supporting Information), suggesting that its O_V_‐enriched architecture provides abundant NO_3_RR active sites, whereas the nitrogen‐containing intermediates are consumed via coupling in the co‐electrolysis process, thus decreasing ammonia production. The quantitative determination of nitrite (NO_2_^−^) generated during the electrocatalytic process was performed via the Griess reagent method, which indicates its low FE. The strategic introduction of O_V_ generates coordinatively unsaturated active sites that strengthen chemisorption of reactive species. Computational analysis confirms that under O_V_‐rich conditions, the adsorption energy of CO_2_ molecules undergoes favorable optimization from 0.53 eV to a more thermodynamically stable −0.26 eV (**Figure** **5**g). Combined with product analysis of singular CO_2_ reduction, *d*‐CeO_x_/PC shows excellent electrocatalytic activity for CO_2_ reduction to CO, attributed to enhanced CO_2_ adsorption coupled with appropriate CO desorption kinetics. Furthermore, the significant reduction of CO formation observed during co‐electrolysis indicates competitive occupation of active sites by NO_3_^−^ reduction intermediates, which subsequently undergo coupling reactions with adsorbed *CO species leading to their consumption (Figure 4b; Figure S18, Supporting Information). Concurrently, *d*‐CeO_x_/PC exhibits substantially lower NH_3_ yield rates and FEs during co‐electrolysis compared to isolated NO_3_RR, implying that the synergy of contracted Ce─O bonds and O_V_ effectively stabilize critical nitrogen‐containing intermediates while inhibiting their deep hydrogenation pathways, thereby promoting favorable C‐N coupling processes essential for subsequent urea formation.

A series of control experiments were implemented to definitively trace the origin of urea generation, encompassing electrocatalytic evaluations under the following conditions: within CO_2_‐saturated 0.1 M KHCO_3_ and Ar‐saturated 0.1 M KNO_3_ electrolytes, at open‐circuit potential conditions, and utilizing blank carbon paper electrodes devoid of catalyst. As illustrated in Figure 4c, no urea was detected after electrolyzing CO_2_‐saturated 0.1 M KHCO_3_ or Ar‐saturated 0.1 M KNO_3_ electrolytes individually. Similarly, urea was also undetectable when carbon paper loaded with *d*‐CeO_x_/PC was immersed in CO_2_‐saturated 0.1 M KNO_3_ electrolyte without applied potential, or when electrochemical tests were performed on catalyst‐free carbon paper in the same electrolyte. Conversely, the most intense characteristic UV absorption peak was exclusively observed subsequent to electrocatalytic operation of *d*‐CeO_x_/PC within CO_2_‐saturated 0.1 M KNO_3_ electrolyte at −1.5 V versus RHE. These results confirm that substantial urea production occurs only when NO_3_^−^ and CO_2_ are used as feedstocks simultaneously, demonstrating that the generated urea originates intrinsically from the electrochemical co‐reduction process rather than extraneous contamination.

In situ attenuated total reflection Fourier transform infrared (ATR‐FTIR) spectroscopy was further used to investigate key intermediates during electrolysis in CO_2_‐saturated 0.1 M KNO_3_ electrolyte at −1.3 V to −1.7 V versus RHE (Figure 4d). Several characteristic absorption bands corresponding to key species along the C‐N coupling pathway were successfully identified. The band at ≈1640 cm^−1^ is assigned to the H‐O‐H bending vibration of adsorbed H_2_O (Figure S19a, Supporting Information).^[^ ^50^ ^]^ Meanwhile, the signal at ≈1770 cm^−1^ is attributed to the N‐O stretching vibration of adsorbed *NO, confirming the effective reduction of NO_3_^−^ to *NO as a crucial precursor for C‐N coupling.^[^ ^26^ ^]^ Additionally, the band at ≈1890 cm^−1^ corresponds to the C‐O stretching vibration of linearly adsorbed *CO, resulting from CO_2_ reduction.^[^ ^51^ ^]^ Notably, a band at ≈2100 cm^−1^ emerges, which is assigned to the C = O stretch in *OCNO/*ONCO‐type intermediates.^[^ ^9^ ^]^ This signals the formation of the first C─N bond, a key step in urea synthesis. Furthermore, the band at ≈2358 cm^−1^ is attributed to the asymmetric stretching of physically adsorbed CO_2_ molecules,^[^ ^52^ ^]^ confirming the availability of CO_2_ near the active sites (Figure S19b, Supporting Information). Remarkably, the more negative the applied potential, the stronger the peak intensity of the intermediates, which reaches a maximum at −1.5 V and then decreases, indicating that the evolution of the intermediates is consistent with the variation in urea yield with applied potential. The evolution of these bands, specifically, the appearance of *NO and *CO followed by the emergence and intensification of the *ONCO signal, provides direct spectroscopic evidence for the coupling between *NO and *CO, supporting “*NO + *CO → *OCNO” as the pivotal C‐N coupling step. Additionally, compared to *d*‐CeO_x_/PC, the in situ ATR‐FTIR spectrum of CeO_2_/PC exhibits weaker or absent detectable intermediate peaks, particularly the C‐N coupling peak (Figure 4e). These in situ observations offer dynamic mechanistic insight and strongly corroborate the reaction pathway.

To gain in‐depth insights into the charge transfer kinetics of the electrocatalyst, electrochemical impedance spectroscopy (EIS) measurements were performed at −1.5 V versus RHE in CO_2_‐saturated 0.1 M KNO_3_ electrolyte. The radius of the Nyquist plot in the high‐frequency region correlates with the charge transfer resistance. A smaller radius typically indicates reduced charge transfer resistance and enhanced electron transfer kinetics.^[^ ^53^ ^]^ Compared with other control samples, *d*‐CeO_x_/PC displays the smallest semicircle and the steepest slope in Nyquist plot, signifying that loading *d*‐CeO_x_ with lattice distortion and O_V_ on PC significantly enhances the catalyst's conductivity and promotes efficient electron transfer across the electrocatalyst‐electrolyte interface, especially under high current densities (Figure 4f). Under 10 mV s^−1^ polarization in CO_2_‐saturated 0.1 M KNO_3_, *d*‐CeO_x_/PC manifests markedly augmented current densities compared to Ar‐saturated electrolyte (Figure S20, Supporting Information), reconfirming the critical mechanistic involvement of CO_2_ and its derived intermediates in the electrocatalytic urea synthesis pathway. Additionally, the electrochemical active area (A_echem_) of *d*‐CeO_x_/PC was determined by cyclic voltammetry (CV). CV measurements were conducted at different scan rates (10‐30 mV s^−1^) within the potential range of −0.3 to −0.5 V versus RHE, and the C_dl_ values of *d*‐CeO_x_/PC and other control samples were obtained, as shown in Figures S21 and S22 (Supporting Information). Based on the C_dl_ values, the A_echem_ was estimated. The results demonstrate that *d*‐CeO_x_/PC possesses the largest electrochemical active area (377.5 cm^2^ mg^−1^), providing more active sites for C‐N coupling to form urea and thus significantly promoting the electrocatalytic performance. Meanwhile, the ECSA‐normalized LSV unequivocally demonstrates that the *d*‐CeO_x_/PC also exhibits a significantly higher current density per unit active surface area compared to the other control samples (Figure S23, Supporting Information). This means that the performance enhancement of the *d*‐CeO_x_/PC originates not merely from a larger surface area but from the superior intrinsic activity of each active site. To further elucidate the rate‐determining step (RDS) of the reaction, Tafel analysis was conducted over a scan rate range of 5–50 mV s^−1^ (Figure S24, Supporting Information). The results reveal a distinct trend: the Tafel slope increases significantly with increasing scan rate, which is indicative of reduced coverage of key reaction intermediates (*CO, *NO, *OCNO) under non‐steady‐state conditions. This observation thus confirms that the C‐N coupling process is highly sensitive to surface intermediate coverage and is therefore likely the RDS. Collectively, these Tafel analysis results confirm that the overall reaction kinetics are controlled by the chemical coupling step rather than by electron transfer processes.

Electrochemical stability represents a critical performance indicator for practical electrocatalyst evaluation. Throughout ten consecutive cycling tests at −1.5 V versus RHE, the current density and urea yield rate of *d*‐CeO_x_/PC exhibit negligible change (Figure 4g), demonstrating exceptional electrochemical robustness. Further, a long‐term stability test was performed over 100 h at −1.5 V versus RHE (Figure S25, Supporting Information). After post‐stability tests, *d*‐CeO_x_/PC was characterized via TEM (Figure S26, Supporting Information), EPR (Figure 4h), XPS (Figure 4i; Figures S27–S29, Supporting Information) and ICP‐OES (Table S3, Supporting Information). Results demonstrate that despite a slight current decay observed after 72 h, TEM reveals no obvious aggregation or morphological changes of *d*‐CeO_x_ nanoparticles. XPS analysis further confirms that the Ce^3+^/Ce^4+^ ratio and O_V_ concentration remain nearly unchanged following the prolonged reaction. EPR spectroscopy exhibits no significant reduction in O_V_‐related signal intensity, confirming the exceptional stability of O_V_ in the distorted lattice without reoccupation. Additionally, ICP‐OES measurements verify negligible Ce species leaching (< 0.1wt.%), demonstrating that the plant‐mediated hyperaccumulation‐confinement strategy effectively anchors metal oxide nanoparticles in the derived carbon framework, thus enhancing structural integrity and suppressing metal dissolution.

Notably, the EPR resonance peak intensity of non‐distorted CeO_x_/PC undergoes significant attenuation following stability testing (Figure S30, Supporting Information), suggesting probable migration of O_L_ or O_C_ species into adjacent vacancy sites during prolonged electrocatalytic operation, consequently leading to partial refilling of O_V_ and reduction in catalytic active O_V_ site density. In contrast, the relationship between O_V_ and lattice distortion in *d*‐CeO_x_/PC is synergistic: the initial formation of O_V_ during synthesis induces local strain and bond contraction, while the creation of lattice distortion further stabilizes these structural defects. This creates a positive feedback loop, resulting in a high and stable concentration of both features. Consequently, the contracted Ce─O bond lengths significantly enhance orbital hybridization between Ce cations and O anions, thereby elevating the energy barrier for O_L_ desorption. This structural modification substantially impedes the migration of neighboring O_L_ atoms to occupy vacancy sites, effectively decelerating O_V_ diffusion and annihilation processes to prolong their functional lifetime. Simultaneously, the high covalent character within these contracted bond regions stabilizes the electronic configuration of Ce^3+^, facilitates efficient electron transfer from Ce^3+^ centers to adsorbed reactive species, accelerates complex multi‐electron transfer reactions, and minimizes O_L_ participation in reaction pathways, thereby collectively stabilizing highly active intermediates throughout the electrocatalytic process. This synergistic effect between stable high‐concentration O_V_ and lattice distortion collectively modulates the electronic structure of active sites, thereby endowing the C‐N coupling with high activity and selectivity. Moreover, compared with the electrocatalysts for C‐N coupling in urea synthesis reported in recent years (Table S4, Supporting Information), *d*‐CeO_x_/PC exhibits excellent electrocatalytic performance, particularly in terms of outstanding stability and relatively high current density.

### 2.3 Mechanistic Studies of Urea Production

Density functional theory (DFT) calculations were systematically performed to elucidate the pivotal role of small‐sized *d*‐CeO_x_ units as highly effective active sites for electrocatalytic urea synthesis via C‐N coupling on *d*‐CeO_x_/PC catalysts (full computational details are provided in the SI). Computational models were constructed based on the crystal parameters of CeO_2_ and *d*‐CeO_x_ supported on PC, specifically designed to investigate the influence of lattice distortion and the formation of O_V_ on the adsorption and activation capabilities toward key reactants. Given that the extensively exposed (111) facet constitutes the predominant active surface in small‐sized CeO_2_ and *d*‐CeO_x_ crystallites, the adsorption characteristics of *NO and CO_2_ on the (111) planes of both *d*‐CeO_x_/PC and CeO_2_/PC were rigorously evaluated as critical descriptors for predicting overall electrocatalytic coupling activity. Differential charge density analysis (Figure 5a,b) demonstrates effective adsorption of both *NO and CO_2_ molecules onto the (111) plane of *d*‐CeO_x_/PC, with adsorption energies of −2.39 and −0.26 eV, respectively. This thermodynamically favorable adsorption facilitates significant electron transfer from electron‐rich regions of the catalyst surface to the oxygen atoms of the adsorbed reactants, stabilizing *NO while activating C═O bonds, crucial for subsequent coupling steps. In contrast, although the (111) plane of CeO_2_/PC exhibits a favorable adsorption energy of −0.38 eV for *NO, it shows a positive adsorption energy of 0.53 eV for CO_2_ (Figure 5c,d). This endothermic adsorption profile severely hinders CO_2_ activation, rendering subsequent critical coupling steps kinetically unfavorable and thereby limiting the coupling activity of CeO_2_/PC. Further detailed analysis of the adsorption behavior for critical reaction intermediates reveals a significant advantage for the (111) plane of *d*‐CeO_x_/PC (Figure 5g). The reactant NO_3_^−^ can also be effectively adsorbed and activated on the (111) plane of *d*‐CeO_x_/PC with an adsorption energy of −0.32 eV, significantly lower than that of 0.62 eV for CeO_2_/PC. Subsequently, the stepwise dissociation processes leading to the formation of key intermediates *NO (via N─O bond cleavage) and *CO (via C = O bond cleavage) exhibit negative adsorption energies at each stage, confirming thermodynamically spontaneous (exothermic). This demonstrates the superior capability of *d*‐CeO_x_/PC not only for initial reactant activation but also for the effective stabilization of these pivotal intermediates. For the decisive C‐N coupling steps governing urea selectivity, *d*‐CeO_x_/PC exhibits stronger adsorption (more negative adsorption energies) for the key coupling intermediates *ONCO (−2.42 eV) and *ONCONO (−3.60 eV) than those of CeO_2_/PC. The low energies underscore the unique efficacy of small‐sized *d*‐CeO_x_ units in stabilizing the complex intermediates formed during the critical C─N bond formation. Collectively, these theoretical findings establish a clear mechanism: the efficient active sites on the (111) plane of *d*‐CeO_x_/PC, arising from the synergistic interplay between contracted Ce─O bonds and positioned O_V_, function as optimal reaction centers. These sites capture reactants, facilitate their activation through efficient electron transfer, and stabilize key intermediates generated along the complex reaction pathway, thereby significantly enhancing the overall electrocatalytic performance for urea synthesis.

Projected Density of States (PDOS) analysis provides deeper insight into the electronic origins of the enhanced adsorption and activation for *d*‐CeO_x_/PC (Figure 5e). Comparative analysis with the (111) plane of CeO_2_/PC (Figure 5f) reveals a significant increase in electronic DOS near the Fermi level for the (111) plane of *d*‐CeO_x_/PC, attributed to the occupation of Ce‐4f orbitals. The increase in PDOS indicates enhanced hybridization between Ce‐4f and O‐2p orbitals, forming highly localized electronic states within the active region. Furthermore, the heightened covalency of the contracted Ce─O bond regions effectively stabilizes the electronic configuration surrounding the Ce^3+^, creating “electron reservoirs”. These reservoirs facilitate electron transfer from O_V_‐adjacent Ce sites to adsorbed species, enabling flexible multi‐step electron transfer during urea synthesis and underpinning superior electrocatalytic activity.

**Figure 6:**
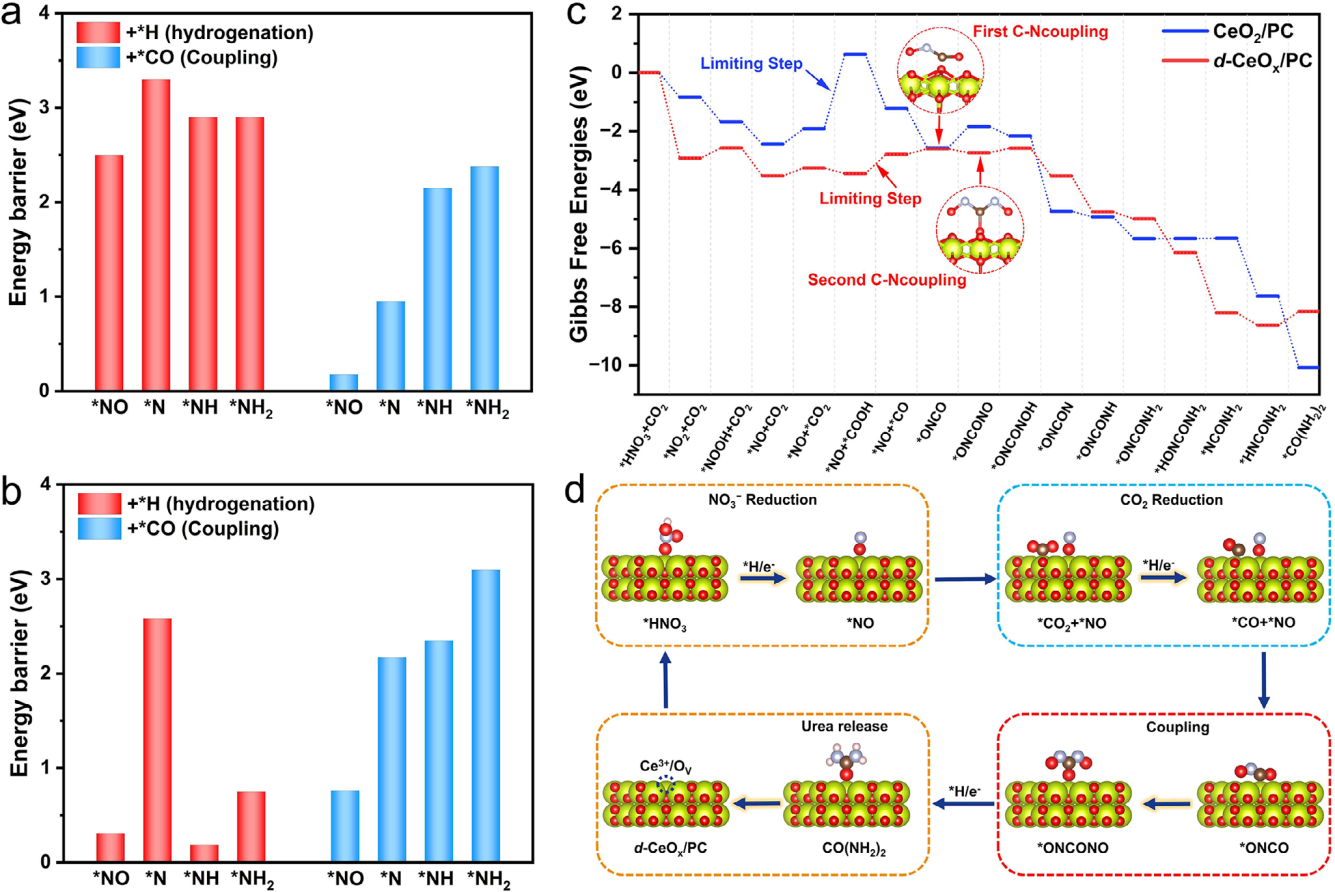
Comparison of the coupling barriers for *NO, *N, *NH, and *NH_2_ coupling with *CO, together with protonation barriers, over a) *d*‐CeO_x_/PC and b) CeO_2_/PC; c) Gibbs free energy diagrams for the entire urea synthesis reaction over *d*‐CeO_x_/PC and CeO_2_/PC; d) Schematic illustration of the reaction mechanism for urea formation via electrocatalytic coupling of NO_3_^−^ and CO_2_. The red, yellow, brown, gray and pink spheres denote O, Ce, C, N, and H atoms, respectively.

Subsequent in‐depth dissection of the intrinsic reaction pathways focused critically on the competitive dynamics between C‐N coupling routes and proton‐coupled electron transfer (PCET) for hydrogenation. As shown in **Figure** **6**a and d‐CeO_x_/PC exhibits significantly lower energy barriers for C‐N coupling of four N‐containing intermediates than corresponding hydrogenation reactions. This energetic difference demonstrates that C‐N coupling on *d*‐CeO_x_/PC with lattice‐distorted and O_V_‐rich structures holds thermodynamic and kinetic advantages over hydrogenation. Notably, the initial *NO/*CO coupling to *OCNO requires only a 0.18 eV barrier, much lower than *OCN (0.95 eV), *OCNH (2.15 eV), or *OCNH_2_ (2.38 eV), forming *OCNO as the favored C‐N coupling intermediate. Conversely, the energies for CeO_2_/PC present less favor for selective urea formation (Figure 6b). Although the initial *NO/*CO coupling proceeds barrier‐free, the subsequent second C‐N coupling (*OCNO+*NO→*ONCONO) encounters a higher barrier. Additionally, energy barriers for forming *OCN (2.17 eV), *OCNH (2.35 eV), or *OCNH_2_ (3.10 eV) via C‐N coupling exceed those for corresponding hydrogenation reactions, which drives N‐containing intermediates on CeO_2_/PC toward hydrogenation, reducing C‐N coupling selectivity and lowering urea yields. Analysis of the optimized adsorption geometries for the *NO intermediate on both electrocatalysts provides structural insight into the origin of differing C‐N coupling efficiencies. For *d*‐CeO_x_/PC, the oxygen atom of adsorbed *NO preferentially embeds into O_V_ site, which exposes only the nitrogen atom, reducing steric hindrance for nucleophilic attack and easing the approach of *CO during C─N bond formation. In contrast, *NO adsorption on CeO_2_/PC occurs without vacancy anchoring, leaving both N and O atoms exposed. The dual exposure creates steric hindrance, impeding the proximity of *CO and optimal orientation for coupling, thus increasing the C─N bond formation barrier. The comprehensive computational analyses presented herein exhibit excellent consistency with the experimental results, supporting a selective electrocatalytic urea synthesis mechanism mediated by the synergistic effects of contracted Ce─O bonds and O_V_ in *d*‐CeO_x_/PC.

The reaction energies for the urea synthesis pathway on both *d*‐CeO_x_/PC and CeO_2_/PC catalysts are comprehensively depicted in Figure 6c (corresponding structural models in Figures S31–S34, Supporting Information). For *d*‐CeO_x_/PC, the rate‐determining step (RDS) barrier is 0.66 eV, with initial C‐N coupling (to *ONCO) requiring only 0.18 eV. Importantly, the second C‐N coupling (to *ONCONO) occurs thermodynamically spontaneously, followed by sequential protonation and reduction to urea. In comparison, CeO_2_/PC has a significantly higher RDS barrier of 2.54 eV. The second C‐N coupling step features a notably higher energy barrier (0.72 eV) than that of *d*‐CeO_x_/PC, demanding substantial energy input for progression. Therefore, the introduction of contracted Ce─O bonds and O_V_ within *d*‐CeO_x_ unit structure can profoundly modulate the adsorption configuration and energetics of key intermediates, effectively suppress competing hydrogenation, lower the barriers for the crucial C─N bond formation, and ultimately optimize the entire reaction for efficient and selective urea synthesis.

The combination of in situ spectroscopy and theoretical calculations provides powerful and direct evidence for the proposal of mechanism. Therefore, a brief summary of the cyclic mechanism for electrocatalytic urea synthesis over *d*‐CeO_x_/PC is presented, as illustrated in Figure 6d: i) *Active Site Generation*: Synergistic effect of contracted Ce─O bonds and O_V_ create electron‐rich active sites, enhancing localized electron density and facilitating electron transfer for catalysis; ii) *NO_3_* ^−^ *Adsorption & Activation*: NO_3_^−^ chemisorbs on active sites, undergoing stepwise reduction to form the key intermediate *NO; iii) *CO_2_ Adsorption & Activation*: CO_2_ chemisorbs on adjacent active sites, activated to form the key intermediate *CO; iv) *First C‐N Coupling*: *NO and *CO undergo kinetically facile coupling to form *OCNO, establishing the first C─N bond; v) *Second C‐N Coupling & Urea Formation*: *OCNO couples with *NO to form *ONCONO, followed by sequential protonation to yield urea.

## 3 Conclusion

In summary, this work successfully developed a novel electrocatalyst, lattice‐distorted CeO_x_ nanoparticles with abundant O_V_ confined within a porous carbon framework (*d*‐CeO_x_/PC), for efficient and selective urea synthesis from the co‐reduction of CO_2_ and NO_3_^−^ under ambient conditions. Leveraging phyto‐hyperaccumulation and structural confinement, followed by Zn^2+^ leaching and controlled annealing, enabled the synthesis of ultrasmall (≈2 nm), uniformly dispersed CeO_x_ nanoparticles exhibiting contracted Ce─O bonds (2.29 vs 2.31 Å) and a high concentration of O_V_. The optimized *d*‐CeO_x_/PC catalyst achieved a remarkable urea yield rate of 532.13 mg h^−1^ g_cat._^−1^ and FE of 35.51% at −1.5 V (vs RHE), representing a substantial improvement over conventional CeO_2_‐based material. Comprehensive characterization and DFT calculations confirmed the critical roles of lattice distortion and O_V_. Specifically, shortened Ce─O bonds intensify hybridization between Ce 4f and O 2p orbitals. This enhanced orbital hybridization restricts the migration of adjacent O_L_ atoms toward O_V_, thereby decelerating O_V_ diffusion/annihilation kinetics and extending O_V_ lifetime. Concomitantly, bond contraction induces localized electronic states and facilitates electron density redistribution near O_V_. Such O_V_ promote Ce^3+^/Ce^4+^ mixed valences, while their high covalency stabilizes Ce^3+^, creating localized “electron reservoirs” that enable flexible multi‐step electron transfer. Moreover, the synergistic effect of lattice distortion and O_V_ significantly lowers the adsorption energies for CO_2_ and NO_3_^−^, facilitates the kinetically favorable C‐N coupling steps (e.g., *NO+*CO→*OCNO), and suppresses competing hydrogenation pathways leading to byproducts like NH_3_. The porous carbon framework further enhances conductivity, mass transport, and active site exposure, contributing to the high stability of the electrocatalyst over prolonged electrolysis. This study demonstrates a rational design strategy that integrates defect engineering, lattice strain, nanoconfinement, and conductive support to overcome the fundamental challenges in electrocatalytic C‐N coupling for urea production. The *d*‐CeO_x_/PC catalyst provides a promising sustainable route for synthesizing valuable organonitrogen compounds using greenhouse gas (CO_2_) and a pollutant (NO_3_^−^) as feedstocks under mild conditions, offering significant energy and environmental advantages over the traditional process.

## 4 Experimental Section

All the experiments are detailed in the Supporting Information.

## Conflict of Interest

The authors declare no conflict of interest.

## Supporting information



Supporting Information

## Data Availability

The data that support the findings of this study are available from the corresponding author upon reasonable request.
