# Supplementary material for: Phyto‐Confined CeOx with Synergistic Lattice Distortion and Oxygen Vacancies Drives Efficient Urea Electrosynthesis
Source: Adv Sci (Weinh). 2025 Nov 4;13(5):e13799. doi: 10.1002/advs.202513799 (PMC12850127; doi:10.1002/advs.202513799)
Supplement: Supplementary file 1 — Supporting Information [file ADVS-13-e13799-s001.docx]

**Supporting Information**

**Phyto**-**Confined CeO_x_ with Synergistic Lattice Distortion and Oxygen Vacancies Drives Efficient Urea Electrosynthesis**

*Ziming Zhao, Yaru Wei, Haoyu Duan, Yuhan Mei, * Huan Li**

Z. Zhao, Y. Wei, H. Duan, Y. Mei, H. Li

Chongqing Institute of Green and Intelligent Technology, Chinese Academy of Sciences, Chongqing 400714, P. R. China

Chongqing School, University of Chinese Academy of Sciences, Chongqing 400714, P. R. China

E-mail addresses: lihuan@cigit.ac.cn (H. Li); meiyuhan@cigit.ac.cn (Y. Mei)

**Experimental section:**

**1. Chemicals**

Cerium nitrate hexahydrate (Ce(NO_3_)_3_·6H_2_O), Zinc nitrate hexahydrate (Zn(NO_3_)_2_·6H_2_O), sodium hydroxide (NaOH), diacetyl monoxime (C_4_H_7_O_2_N), thiosemicarbazide (CH_5_N_3_S), potassium nitrate (KNO_3_), urea (CH_4_N_2_O), salicylic acid (C_7_H_6_O_3_), sodium nitroferricyanide dihydrate (C_5_FeN_6_Na_2_O·2H_2_O), sodium citrate dihydrate (C_6_H_5_Na_3_O_7_·2H_2_O) and potassium bicarbonate (KHCO_3_) were purchased from Shanghai Aladdin Biochemical Technology Co., Ltd. Dimethylaminobenzaldehyde (C_9_H_11_NO), ammonium chloride (NH_4_Cl), sodium hypochlorite (NaClO) and nafion (5 wt%) were purchased from Shanghai Macklin Biochemical Technology Co., Ltd. Carbon papers (Toray) were purchased from Guangzhou Kehua Scientific Instrument Trade Co., Ltd. Nafion 211 membrane (DuPont) were purchased from Guangzhou Huayu Trading Co., Ltd. High purity CO_2_ (99.999%) and Ar (99.999%) were purchased from Chongqing Lituo Gas. None of the chemicals were further purified. Deionized water was used throughout the experiment.

**2. Electrocatalysts characterization**

The samples were comprehensively characterized using scanning electron microscope (SEM), transmission electron microscopy (TEM), powder X-ray diffraction (XRD), X-ray photoelectron spectroscopy (XPS), electron paramagnetic resonance (EPR) spectroscopy, inductively coupled plasma-optical emission spectroscopy (ICP-OES), X-ray absorption fine structure (XAFS) spectroscopy and Brunauer-Emmett-Teller (BET) specific surface area analysis. SEM images were collected by thermal field emission scanning electron microscopy (JSM-7800F JEOL). TEM images and EDS elemental mapping were carried out by a JEM-2100PLUS microscope, with samples prepared by ultrasonic dispersion of the powder in ethanol followed by drop-casting onto a copper grid for drying. XRD spectroscopy were conducted on an X’Pert3 Powder X-ray diffractometer using Cu Kα radiation over a 2θ range of 10°–90°. Surface chemical states were analyzed via XPS with thermo Scientific K-Alpha spectrometer, while BET surface areas were determined from N_2_ adsorption isotherms at 77 K using a Belsorp-max. Vacancy structures were characterized by EPR on an ER200DSRC10/12 spectrometer. Content of elements were characterized by ICP-OES on an Agilent 7700x. X-ray absorption fine structure (XAFS), extended X-ray absorption fine structure (EXAFS) and X-ray absorption near-edge structure (XANES) were characterized by the Shanghai Synchrotron Radiation Facility (SSRF), specifically using the BL14B1 beamline equipped with a high-energy X-ray diffraction system. Additional analyses included UV-Vis absorbance spectra of the electrolyte recorded by a Lambda 35 spectrophotometer, and electrocatalytic synthesis of urea performed in CO_2_-saturated 0.1 M KNO_3_ using a VersaSTAT3F electrochemical workstation.

**3. The synthesis of electrocatalysts**

*3.1 Absorption of metal ions*

The living *Sphagnum* moss with superhydrophilic porous structures was selected as the carrier precursor. 1 g of cleaned *Sphagnum* moss was immersed in a mixed solution containing 0.1 M of Ce(NO_3_)_3_ and Zn(NO_3_)_2_ (Ce^3+^/Zn^2+^ molar ratio 95:5) for three days. Leveraging its hyperaccumulation capacity for metal ions during the process, Ce^3+^ and Zn^2+^ ions were transported and dispersed into the moss tissues. Subsequently, during freeze-drying at −60°C, 1 Pa for 72 h, the osmotic pressure gradient (intracellular > extracellular) drove the migration of Ce^3+^ and Zn^2+^ ions toward cell walls and membrane interfaces. Combined with vacuum sublimation dehydration, this process preserved the three-dimensional porous architecture of the plant skeleton. Similarly, an identical procedure was followed for the control sample, where 1 g of *Sphagnum* moss was immersed in a 0.1 M Ce(NO_3_)_3_ solution exclusively, followed by freeze-drying under the same conditions for subsequent use.

*3.2* *Preparation of* *CeO_2_ supported on PC (CeO_2_/PC)*

The *Sphagnum* moss that only absorbed Ce(NO_3_)_3_ from the previous step was ground into fine powder. Under an Ar atmosphere, the powder was heated to 800℃ at a ramping rate of 5℃ min^-1^ and calcined for 4 h. The fabricated composite underwent a thorough washing process to remove different soluble salts (such as Na^+^, K^+^ ions, etc.), and then dried at 60 °C for 12 h to obtain CeO_2_/PC.

*3.3 Preparation of CeO**_x_ with O_V_ supported on PC (CeO_x_/PC)*

The CeO_2_/PC composite obtained from *step 2.2* was heated to 800℃ at a rate of 5℃ min^-1^ in an Ar/H_2_ (9:1) reducing atmosphere and calcined for 1 h. Subsequently, the product was washed three times with water and ethanol, respectively, and then dried at 60°C for 12 h, yielding the CeO_x_/PC.

*3.4 Preparation of CeO_2_ with lattice distortion supported on PC (**d-CeO_2_/PC)*

The *Sphagnum* moss that absorbed Ce(NO_3_)_3_ and Zn(NO_3_)_2_ from *step 2.1* was ground into fine powder. The powder was then heated to 800°C at a ramping rate of 5°C min^-1^ under an Ar atmosphere and calcined for 4 h. Subsequently, 50 mg of the obtained powder sample was dispersed in 100 mL of deionized water, and 50 mL of 3 M NaOH solution was dropwise added to the dispersion. The mixture was subjected to ultrasonic etching for 1 h to completely remove Zn^2+^ ions (< 0.1 wt%) at room temperature. After thorough washing to eliminate soluble salts, the sample was dried at 60 °C for 12 h, obtaining the *d*-CeO_2_/PC.

*3.5 Preparation of CeO_x_ with O_V_ and lattice distortion supported on PC (d-CeO_x_/PC)*

The *d*-CeO_2_/PC composite derived from *step 2.4* was subjected to thermal treatment under a reducing atmosphere of Ar/H_2_ (9:1). Specifically, it was heated to 800°C at a rate of 5°C min^-1^ and maintained at this temperature for 1 h. The resulting material was then sequentially washed three times with deionized water and ethanol to remove any residual impurities. Afterward, the product was dried at 60 °C for 12 h, resulting in the formation of *d*-CeO_x_/PC.

*3.6* *Preparation of pure CeO_2_*

The pure CeO_2_ nanoparticles were synthesized via a hydrothermal approach. Initially, Solution A was prepared by dissolving 0.89 g of Ce(NO_3_)_3_·6H_2_O in 20 mL of deionized water. Concurrently, Solution B was prepared by dissolving 8.40 g of NaOH in 15 mL of deionized water. Under continuous magnetic stirring at ambient temperature, Solution B was slowly dropped into Solution A at a flow rate of 0.5 mL/min. The resulting homogeneous mixture was further stirred for 30 minutes to ensure complete reaction. The white precipitate obtained was thoroughly washed multiple times with ethanol and deionized water to remove impurities. The purified product was then freeze-dried to preserve its nanostructure. Finally, the dried powder was placed in a tube furnace and annealed under an argon atmosphere at 500°C for 4 hours, with a heating rate of 5°C/min. This annealing process ensured the formation of pure CeO_2_ nanoparticles with structural integrity.^[1]^

**4. Electrochemical measurements**

Electrochemical evaluations were conducted using a three electrode H-cell configuration on an electrochemical workstation (VersaSTAT3F). A Nafion 211 membrane served as the ion-exchange separator between the anode and cathode compartments. Prior to use, the membrane underwent a pretreatment: it was first boiled in ultrapure water for 1 h, then heated in a 5% H_2_O_2_ aqueous solution at 80 °C for 1 h, followed by treatment in 0.05 M H_2_SO_4_ for 1 h and ultrasonic washing with ultrapure water for 1 h.

To fabricate the working electrode, 5 mg of the electrocatalyst was dispersed in a mixture of 950 μL of isopropanol-water solution (volume ratio 2:1) and 50 μL of 5 wt% Nafion aqueous solution through 30 minutes of sonication, forming a homogeneous catalyst ink. Subsequently, 50 μL of catalyst ink was dripped onto a piece of carbon paper and allowed to air-dry. The resulting working electrode had a geometric area of 0.1 cm^2^, corresponding to a catalyst loading of 0.1 mg cm^-2^. An Ag/AgCl electrode was employed as the reference electrode, while a carbon rod functioned as the counter electrode.

Prior to the experiments, the cathode electrolyte was purged with CO_2_ at a flow rate of 50 mL min^-1^ for 30 minutes to ensure saturation. During the electrocatalysis process, the CO_2_ flow rate was maintained at 30 mL min^-1^, and the electrolyte was continuously stirred at 350 rpm. Applied potentials were measured relative to the Ag/AgCl reference electrode and then converted to the reversible hydrogen electrode (RHE) scale using the formula E_RHE_ = E_Ag/AgCl_+0.0591pH + 0.197. Controlled potential electrolysis experiments were carried out at each specified potential for 60 minutes. The electrochemical reactions were carried out in 0.1 M KNO₃ electrolyte, with 20 mL of the solution placed in both the anode and cathode chambers.

A series of electrochemical tests were performed: cyclic voltammetry (CV) was conducted at a scan rate of 10 to 30 mV s^-1^, with the potential range spanning from –0.5 to – 0.3 V *vs.* RHE; linear sweep voltammetry (LSV) was carried out at a scan rate of 20 mV s^-1^; chronoamperometric tests were performed at various applied potentials while continuously supplying CO_2_ to the cathodic cell. Additionally, electrochemical impedance spectroscopy (EIS) with a frequency range from 100 kHz to 0.01 Hz measurements were executed in a three-electrode configuration. The 0.1 M KNO₃ electrolyte was saturated with CO_2_ prior to and during the EIS tests.

**5. Determination of products**

*Determination of urea concentration:*^[2,3]^ The urea concentration was quantified using the diacetyl monoxime method as described in reference. Specifically, 1 mL of the sample solution was extracted from the cathodic chamber and transferred to a test tube. To this, 1 mL of diacetylmonoxime-thiosemicarbazide (DAMO-TSC) reagent (prepared by dissolving 5 g of DAMO and 100 mg of TSC in distilled water, followed by dilution to 1000 mL) and 2 mL of acid-ferric solution (prepared by mixing 100 mL of concentrated phosphoric acid, 300 mL of concentrated sulfuric acid, and 600 mL of distilled water, then dissolving 100 mg of FeCl_3_ in the mixture) were sequentially added. The solution was thoroughly mixed, heated to 100°C, and maintained at this temperature for 15 minutes. After cooling to room temperature, the UV-vis absorption spectrum was recorded at a wavelength of 525 nm for quantitative analysis. The Faradaic efficiency (FE) is defined as the ratio of the number of electrons consumed for urea formation to the total charge passed through the circuit. Given that sixteen electrons are theoretically required for the synthesis of one urea (CO(NH_2_)_2_) molecule, the FE and urea yield rate can be calculated using the following equations:

$$\text{Yield rate}_{\text{urea}}\text{=}\frac{\text{C}_{\text{urea}}\text{×}\text{V}}{\begin{aligned} \text{m}_{\text{cat.}}\text{×}\text{t} \end{aligned}}$$

$$\text{ }\text{ }\text{FE}_{\text{urea}}\text{=}\frac{\text{16}\text{×}\text{F}\text{×}\text{C}_{\text{urea}}\text{×}\text{V}}{\text{60.06×}\text{Q}}$$

*F* represents the Faraday constant, *Q* denotes the total electric charge passed, *C_urea_* is the concentration of synthesized urea, *V* is the volume of the electrolyte, *t* is the duration of the electrolysis, and *m_cat._* is the mass of the catalyst loaded. The urea formation rate was calculated on an hourly basis and reported as the average value over the entire testing period.

*Determination of ammonia concentration:*^[4,5]^ The ammonia yields were quantified via the indophenol blue method as detailed in reference, where 1 mL of 0.05 M NaClO and 0.2 mL of 1% aqueous Na_2_[Fe(CN)_6_]·2H_2_O were added to 2 mL of 1 M NaOH solution containing 5% salicylic acid (C_7_H_6_O_3_) and 5% trisodium citrate dihydrate (C_6_H_5_Na_3_O_7_·2H_2_O) as the chromogenic agent. Subsequently, 2 mL of electrolyte extracted from the cathodic chamber was mixed with 2 mL of the chromogenic agent and its absorbance at 655 nm was measured to determine ammonia concentration via the calibration curve, from which the ammonia yield was calculated using the following formula:

$$\text{ Yield rate}_{\text{NH}_{\text{3}}}\text{=}\frac{\text{C}_{\text{NH}_{\text{3}}}\text{×}\text{V}}{m\text{×}t}$$

$$\text{FE}_{\text{NH}_{\text{3}}}\text{=}\frac{\text{8}\text{×}\text{F}\text{×}\text{C}_{\text{NH}_{\text{3}}}\text{×}\text{V}}{\text{17×}\text{Q}}$$

*C_NH3_* denotes the measured ammonia concentration, *V* is the total volume of the electrolyte, t represents the electrocatalysis duration, m is the mass of the loaded catalyst, F is the Faraday constant, and Q signifies the total charge passed through the working electrode.

*Determination of nitrite concentration:*^[6,7]^ The nitrite (NO_2_⁻) concentration is determined via Griess assay. Briefly, 2 mL of electrolyte is mixed with 0.1 mL of 4-aminobenzenesulfonamide (10 g L^-1^ in 10 wt% HCl), reacted for 8 min, followed by addition of 0.1 mL N-(-1-naphthyl) ethylenediamine dihydrochloride aqueous solution (1 g L^-1^) and further reaction for 10 min in the dark. Absorbance was measured at 540 nm using UV-vis spectrophotometry. Concentration is quantified using a pre-established calibration curve from standard NaNO_2_ solutions. nitrite FE was calculated using the following formula:

$$\text{FE}_{\text{nitrite}}\text{=}\frac{\text{2}\text{×}\text{F}\text{×}\text{C}_{\text{nitrite}}\text{×}\text{V}}{\text{46}\text{×}\text{Q}}$$

*C_nitrite_* denotes the measured nitrite concentration, *V* is the total volume of the electrolyte, F is the Faraday constant, and Q signifies the total charge passed through the working electrode.

*Determination of Gaseous products:* The gas products (including CO and H_2_) of electrocatalytic the coupling reaction of NO_3_⁻ and CO_2_ were quantitatively analyzed using an Agilent 7890A gas chromatograph. The system employed a thermal conductivity detector (TCD) for H_2_ quantification and a flame ionization detector (FID) equipped with a methanizer for CO detection. The analytical protocol involved backflushing non-methane hydrocarbons (NMHCs) after the elution of CO, CO_2_, and CH_4_, which were resolved as distinct peaks. Carbon-containing species were catalytically converted to methane in a methanizer maintained at 270 °C, enabling sensitive FID detection.

**6. Electrochemical active surface areas (A_echem_) and** **impedance spectroscopy (EIS)**

The electrochemical double-layer capacitance (C_dl_) of various samples was evaluated via cyclic voltammetry (CV) experiments in CO_2_-saturated 0.1 M KNO_3_ electrolyte. This approach was employed to estimate the electrochemical active surface area (A_echem_) of the samples by examining the non-Faradaic region. The CV tests were executed across a potential range of −0.3 to −0.5 V versus the reversible hydrogen electrode (RHE) at multiple scan rates, namely 10 mV s^-1^, 15 mV s^-1^, 20 mV s^-1^, 25 mV s^-1^, and 30 mV s^-1^.​ The capacitive current, calculated as Δj (where Δj represents the difference between the anodic current (j_a_) and cathodic current (j_c_) at identical potentials), was determined at −0.4 V vs. RHE. Subsequently, these capacitive current values were plotted against the corresponding scan rates. The slope of the resulting linear fit directly corresponds to the C_dl_ value. Electrochemical active area (ECSA) was performed using the formula:

ESCA= C_dl_/ C_std_

Where C_dl_ is the double-layer capacitance for catalyst, and C_std_ is the specific capacitance for a flat surface, taken as the standard value of 0.040 mF cm^-2^. It is crucial to emphasize that these comparative analyses are valid only when all samples are measured under consistent experimental conditions.​

ECSA-normalized LSV performance comparisons of on different catalysts:

To compare the intrinsic activity, ECSA-normalized the LSV curves were performed according to the electrochemical active area (ECSA). The normalization was performed using the formula:

J _ECSA_ = J _geo_ / ESCA

where J _geo_ is the current density normalized by the geometric area (mA cm^-2^). ^[8, 9]^

In addition, electrochemical impedance spectroscopy (EIS) measurements were conducted under operational conditions at a potential of −1.5 V vs. RHE. A sinusoidal voltage with an amplitude of 5 mV was applied, and the frequency sweep spanned from 100 kHz to 0.01 Hz. Through this process, the geometric values of the charge transfer resistance (R_ct_) and series resistance (R_s_) were accurately measured.

**7. The turnover frequency (TOF) calculation**

The turnover frequency (TOF) per active site was calculated using the following formula：^[10,11]^

$$TOF=\frac{{number of total urea turnovers/cm}^{2} of geometric area}{{number of active sites/cm}^{2} of geometric area}$$

The number of urea turnovers was derived from the current density via the equation：

$$no. of urea=$$

$$\left( j\frac{mA}{{cm}^{2}} \right)\left( \frac{1C s^{-1}}{1000 mA} \right)\left( \frac{{1 mol of e}^{-1}}{96485.3 C} \right)\left( \frac{1 mol of urea}{16 mol ofe^{-}} \right)\left( \frac{{6.022 mol \times10}^{23} urea moleculars}{1 mol urea} \right)$$

$$=1.95\times{10}^{14}\frac{urea/s}{{cm}^{2}}per\frac{mA}{{cm}^{2}}$$

The Ce^3+^-OV as the catalytic active sites, the low-coordinated Ce^3+^ ions on the *d*-CeO_x_ surface were identified as the active species in the *d*-CeO_x_/PC catalyst. Based on the electrochemical active area of *d*-CeO_x_/PC and the unit cell structure of *d*-CeO_x_ (volume (12.01 Å^3^) is calculated from the XAFS results), the TOF value was estimated as follows:

$${Active sites}_{d-CeOx/PC}={Active sites}_{d-CeOx}=\left( \frac{12 atom/unit cell}{12.01 Å^{3}/unit cell} \right)\mathrm{atoms}\mathrm{cm}^{-2}$$

$$TOF=\frac{{(1.95\times{10}^{14}\frac{urea/s}{\mathrm{cm}^{2}}\mathrm{per}\frac{\mathrm{mA}}{\mathrm{cm}^{2}})cm}^{2}}{Active sites\times A_{\mathrm{echem}}}\times│j│$$

**8. Density functional theory (DFT) calculation**

Computational Methods：

Density functional theory (DFT) calculations were performed using Vienna Ab initio Simulation Package (VASP)^[12,13]^ based on density functional theory. The projector augmented wave (PAW) pseudopotential method^[14,15]^ and the Perdew-Burke-Ernzerhof (PBE) exchange-correlation functional^[16]^ were employed, and the Hubbard U correction was applied to the 4f electrons of Ce atoms. U = 4.5 eV was chosen in the calculations. A plane-wave cutoff of 500 eV was employed for all calculations, and the Gaussian-smearing was employed with a smearing width of 0.1 eV. The electronic and ionic relaxations convergence criteria were 10^-4^ eV and 0.05 eV/Å. The CeO_2_ (CeO_2_/PC) unit cell was calculated to have the bulk lattice constants of a = b = c = 5.40 Å. A 2×2 3-layer CeO_2_ (111) surface was modeled for the DFT calculation with a vacuum space of about 6.9 Å along the z direction. The *d*-CeO_x_ (*d*-CeO_x_/PC) unit cell was calculated to have the bulk lattice constants of a = 5.23 Å, b = c = 5.25 Å. A 2×2 3-layer *d*-CeO_x_ (111) surface was modeled for the DFT calculation with a vacuum space of about 6.8 Å along the z direction, and the O-vacancy model was also created by removing one O atom from the *d*-CeO_x_ (111) surface. The top two layers together with the adsorbates were allowed to fully relax, while the bottom layer was fixed during optimization for all surface calculations. A Gamma centered k-point grid of 2×2×1 was used to sample the reciprocal space for surface calculations, and a denser grid of 4×4×1 was employed for density of states (DOS) calculations.

Energy Calculations：

The adsorption energies (${\Delta E}_{ads}$) involving gas-phase species were determined according to the following formula:

$${\Delta E}_{ads}=E_{surface+adsorbate}-E_{surface}-E_{adsorbate(gas)}$$

Here $E_{surface+adsorbate}$ is the free energy of the adsorbate–surface system, $E_{surface}$ is the free energy of the bare surface, and $E_{adsorbate(gas)}$ is the free energy of the gas-phase adsorbate molecule. The Gibbs free energy of reaction ($\Delta G$), which was based on the hydrogen electrode (CHE) model proposed by Nørskov et al.,^[17,18]^ was computed to assess the catalytic activity of urea synthesis reactions, i. e.

$$\Delta G=\Delta E+{\Delta E}_{ZPE}+\Delta H-T\Delta S-eU+{\Delta G}_{pH}$$

Where $\Delta E$ is the electronic energy obtained from the DFT calculations, ${\Delta E}_{ZPE}$ represents the change in zero-point energy change, while $\Delta H$ and $T\Delta S$ are the enthalpic and entropic corrections, respectively. T = 298.15 K. The zero-point energy, enthalpic and entropic corrections were calculated from the vibrational frequencies. $e$ and $U$are the number of electrons transferred and the applied electrode potential, respectively. ${\Delta G}_{pH}$ is the energy correction of pH. In the context of this work, the free energy of a proton or an electron was equal to half the free energy of H_2_ (g) at an applied electric potential U = 0 V vs. SHE and pH = 0. To compute the free energies in urea synthesis reactions, we employed this formula for reaction *A + *B→*AB:

$${\Delta G}_{rxn}=G_{*AB}+G_{*}-G_{*A}-G_{*B}$$

Here $G_{*A}$ and $G_{*B}$ are the free energies of species A and species B adsorbed on the surface. $G_{*AB}$ is the free energy of species AB adsorbed on the surface, and $G_{*}$ is the free energy of the clean surface. The limiting potential (U_L_) is the applied potential that can make every elementary step exergonic in the reaction mechanism, and was described by the following equation: ^[19,20]^

$$U_{L}={-\Delta G}_{max}/e$$

${-\Delta G}_{max}$ is the free-energy change for the rate limiting step along the assumed urea synthesis pathway.

**
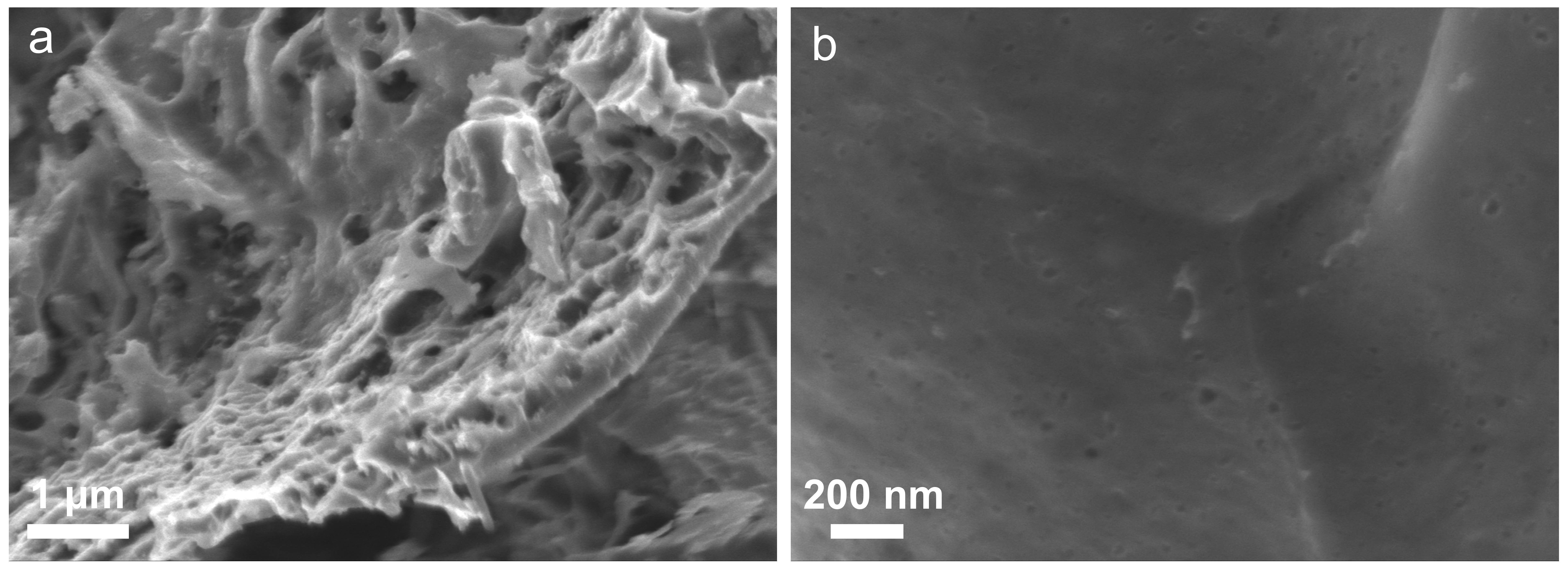
**

**Figure S1.** (a, b) SEM images of *d*-CeO_x_/PC.


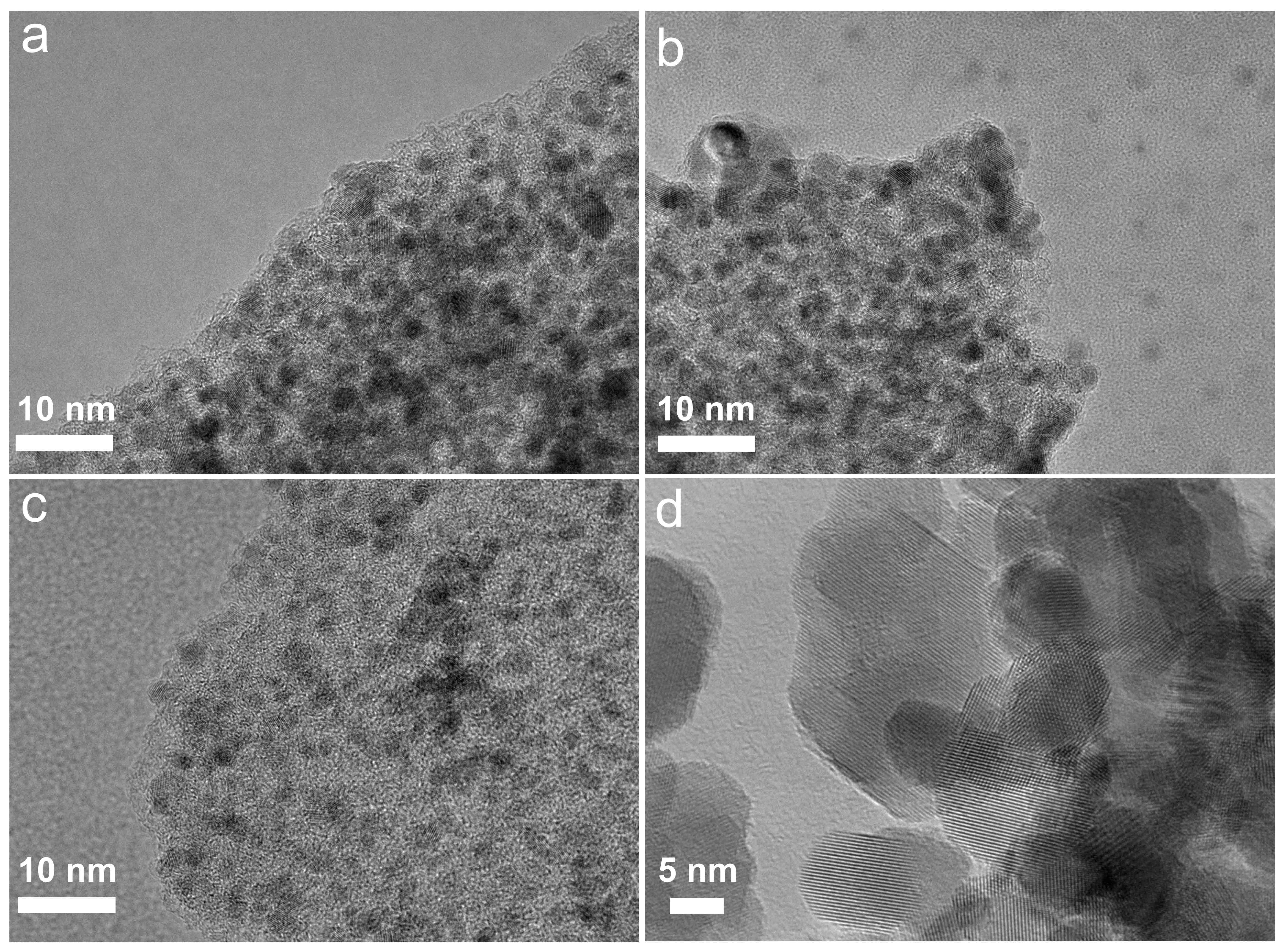


**Figure S2.** (a) TEM image of CeO_2_/PC; (b) TEM image of CeO_x_/PC; (c) TEM image of *d*-CeO_2_/PC; (d) TEM image of pure CeO_2_ nanoparticles.

**
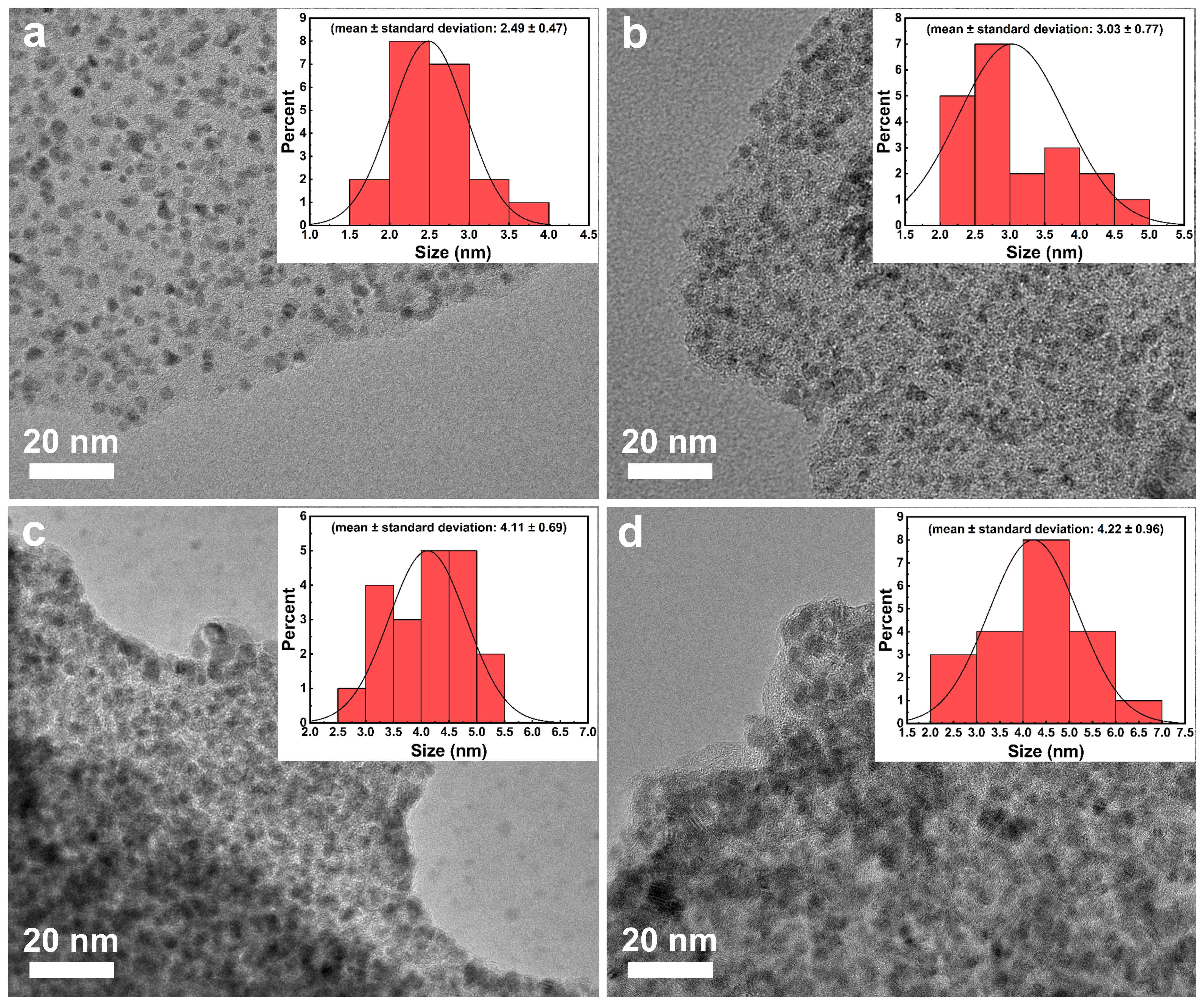
**

**Figure S3.** TEM image and corresponding grain size distribution statistics (including mean ± standard deviation) of (a) *d*-CeO_x_/PC, (b) *d*-CeO_2_/PC, (c) CeO_x_/PC and (d) CeO_2_/PC.

**
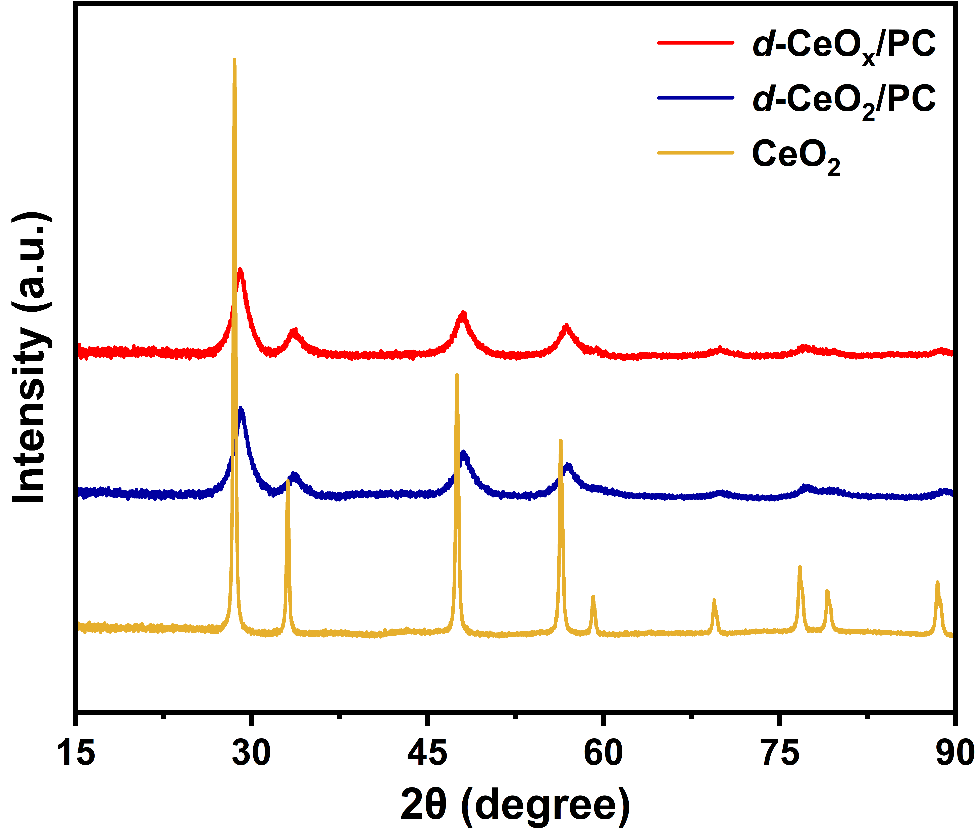
**

**Figure S4.** XRD spectra of pure CeO_2_, *d*-CeO_2_/PC and *d*-CeO_x_/PC.





**Figure S5.** XRD refinement (a) *d*-CeO_x_/PC, (b) CeO_x_/PC, (c) *d*-CeO_2_/PC and (d) CeO_2_/PC.

**
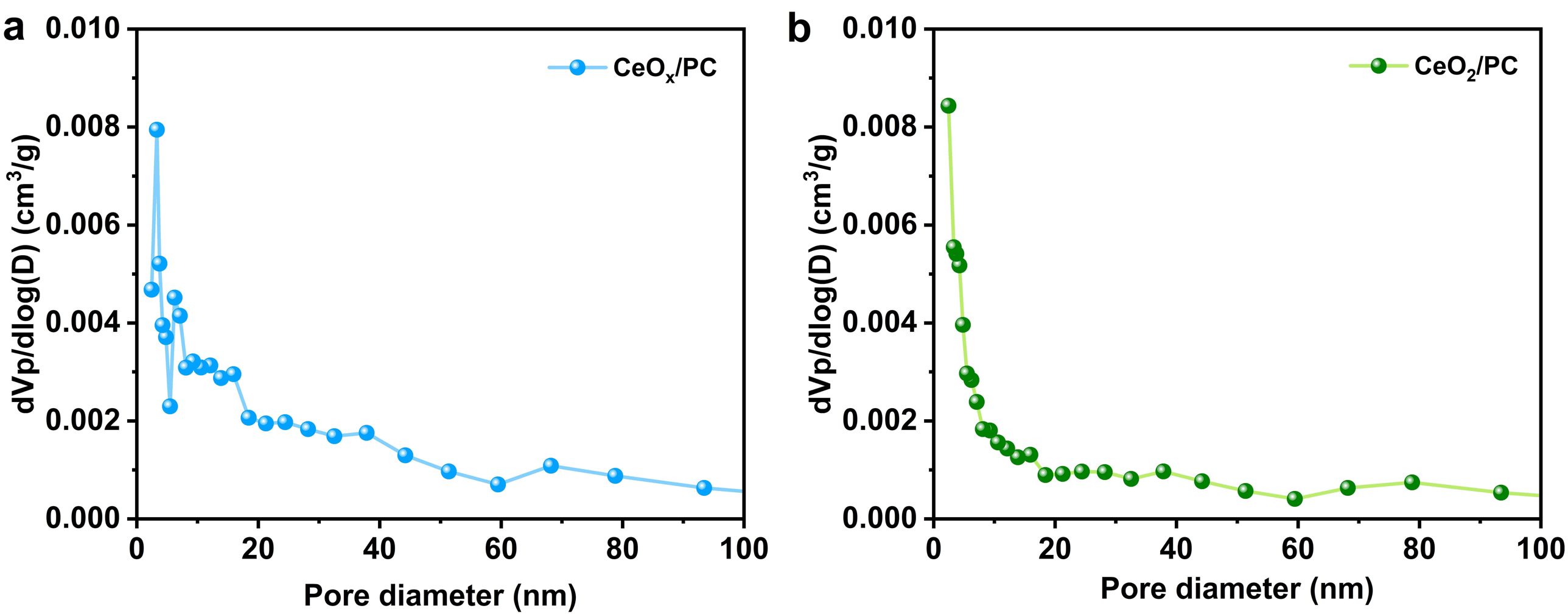
**

**Figure S6.** BJH pore size distribution of (a) CeO_x_/PC and (b) CeO_2_/PC.

**
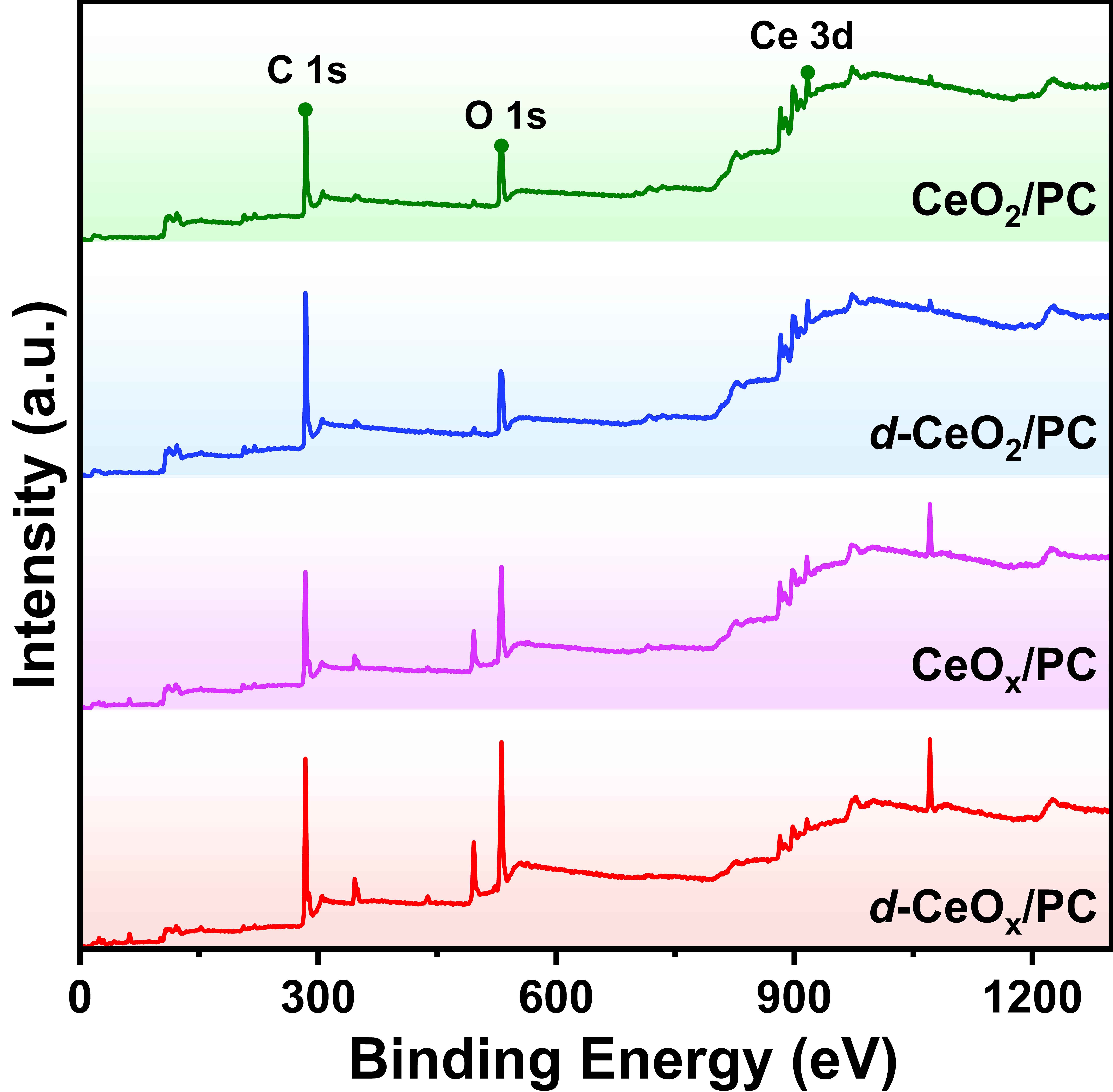
**

**Figure S7.** XPS survey spectrum of CeO_2_/PC, *d*-CeO_2_/PC, CeO_x_/PC and *d*-CeO_x_/PC.


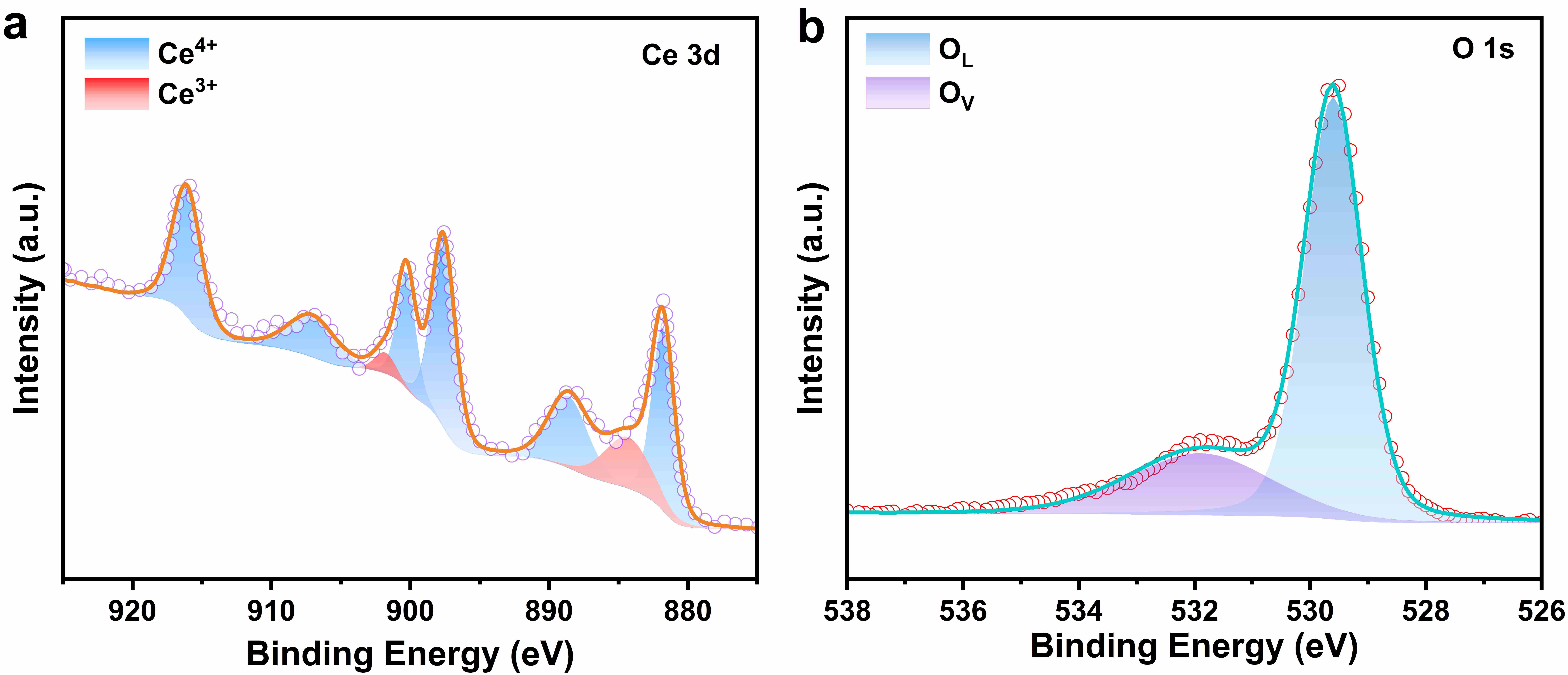


**Figure S8.** (a) High resolution XPS spectra of Ce 3d and (b) O1s of pure CeO_2_.


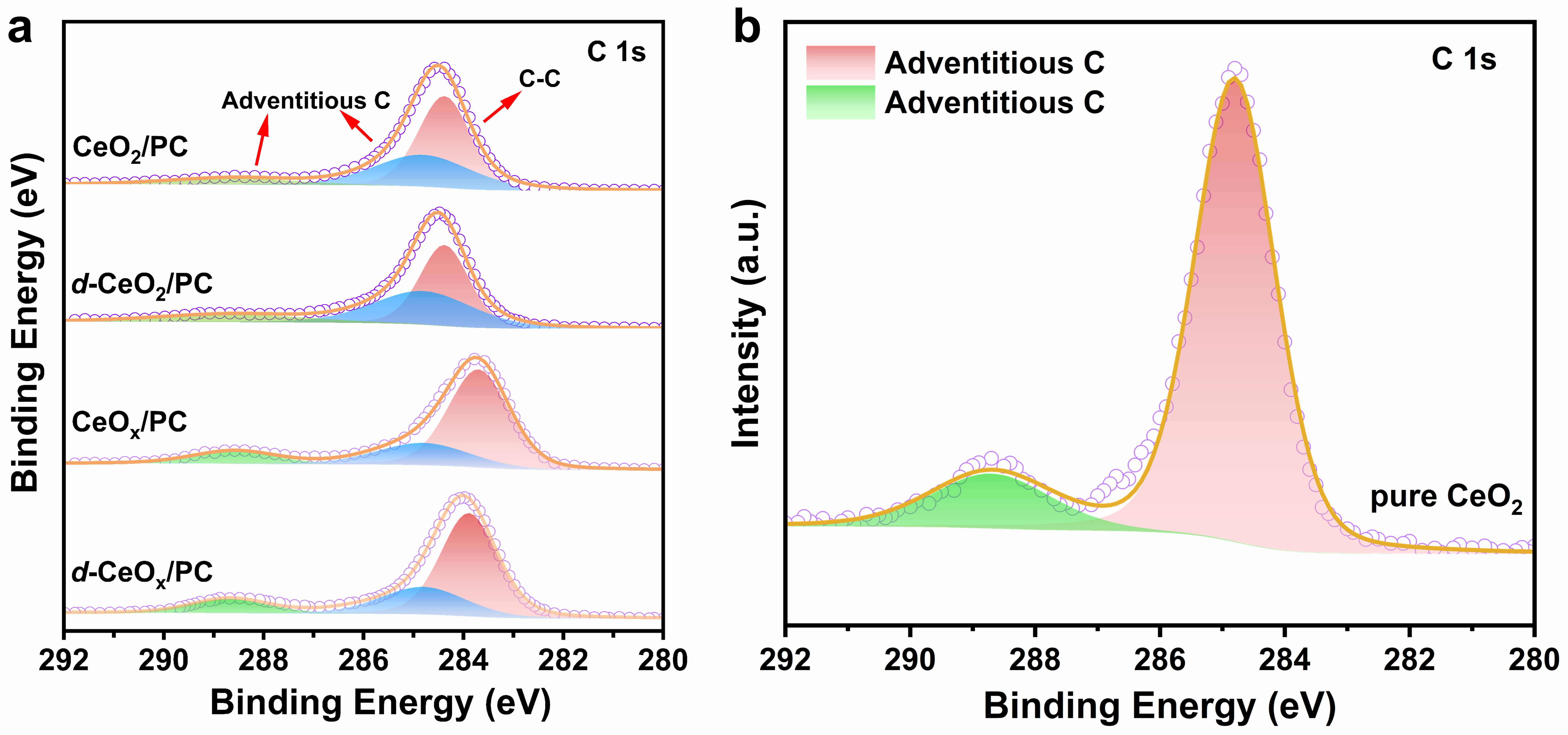


**Figure S9.** (a) High resolution XPS spectra of C 1s of CeO_2_/PC, *d*-CeO_2_/PC, CeO_x_/PC and *d*-CeO_x_/PC and (b) C 1s of pure CeO_2_.


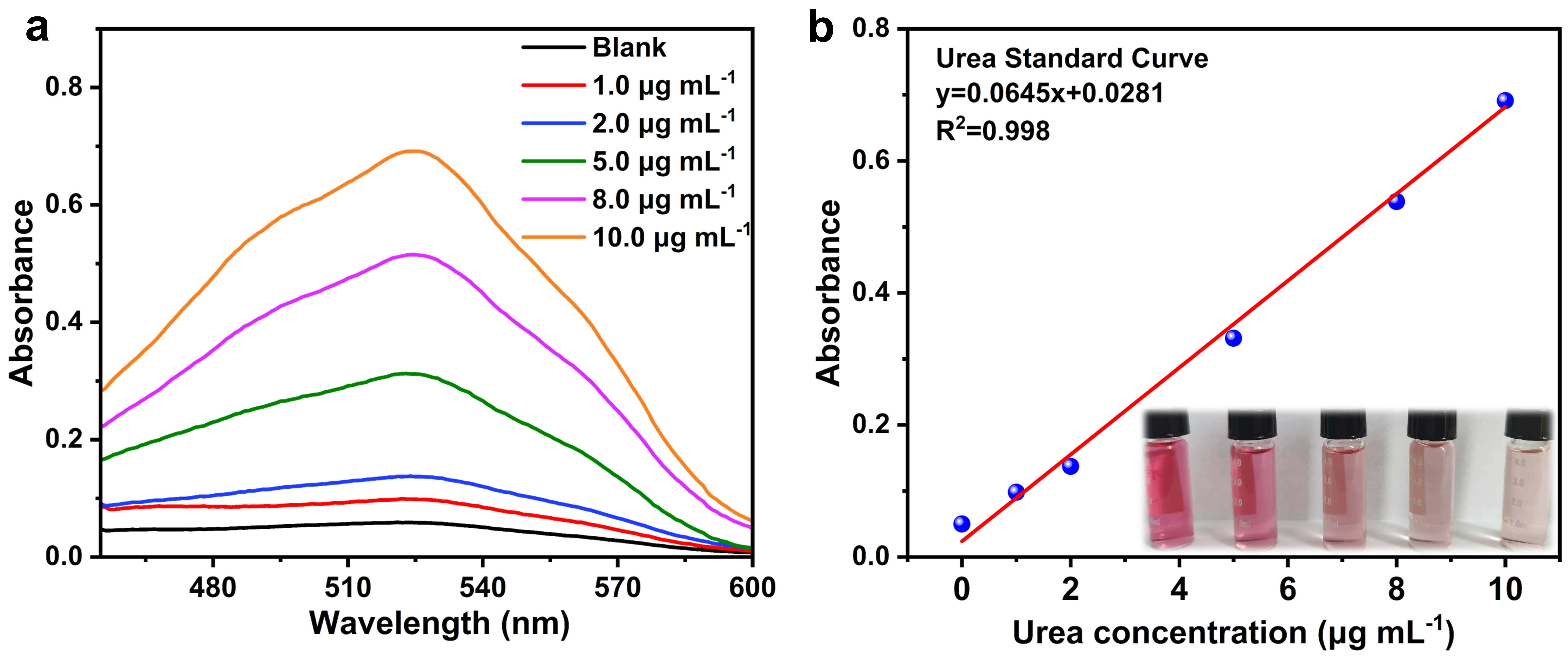


**Figure S10.** (a) UV-vis spectra from diacetyl monoxime assays of urea following heating at 100 °C for 15 min. (b) Concentration-absorbance curves of urea solutions with gradient standard concentrations in 0.1 M KNO_3_. Absorbance at 525 nm was recorded using a UV-vis spectrophotometer, and the standard curve exhibits a strong linear correlation between absorbance and urea concentration (y = 0.0645x + 0.0281, R^2^ = 0.998).

**
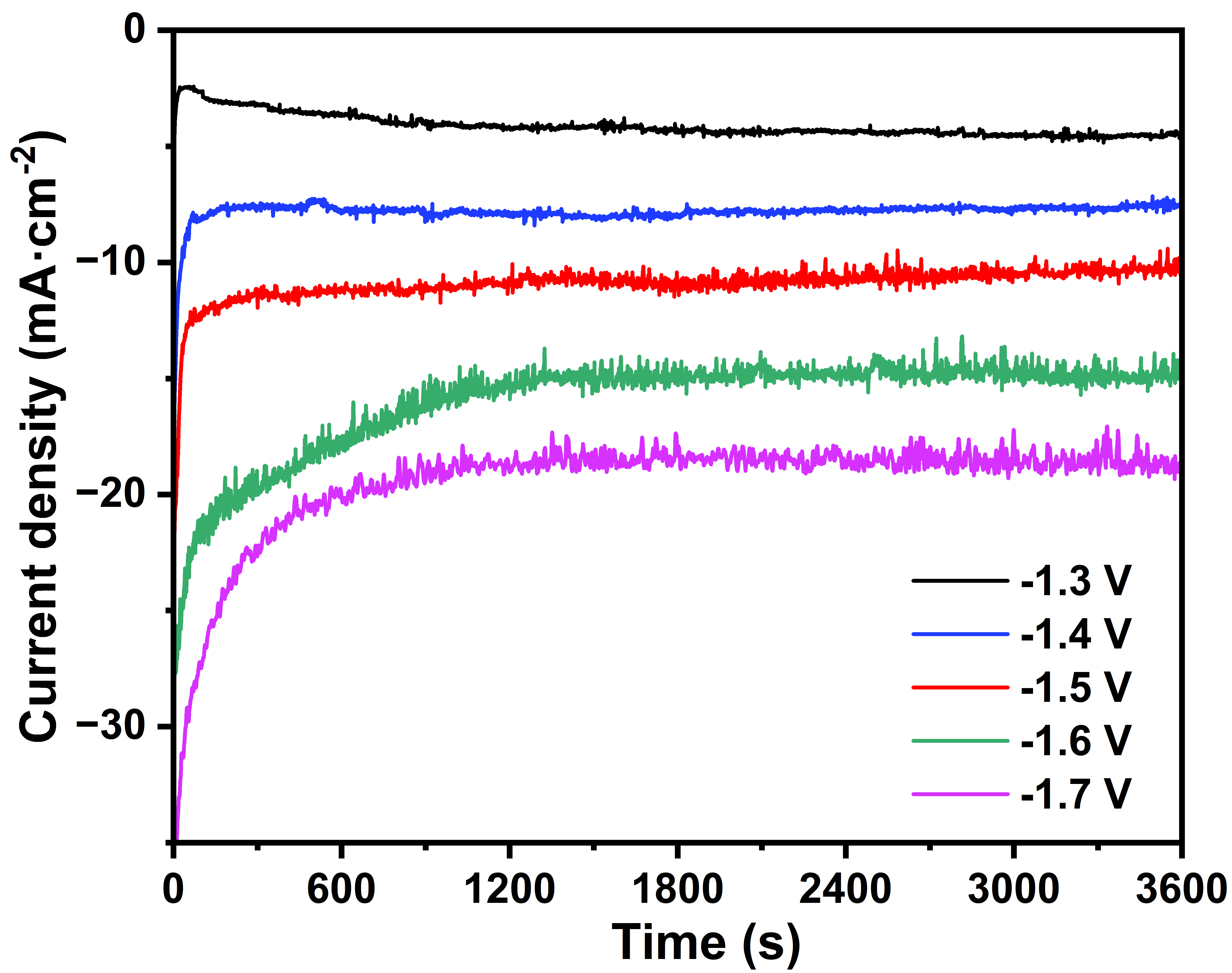
**

**Figure S11.** Chronoamperometric profiles of *d*-CeO_x_/PC recorded 1 h at a series of potentials ranging from −1.3 to −1.7 V vs. RHE.

**
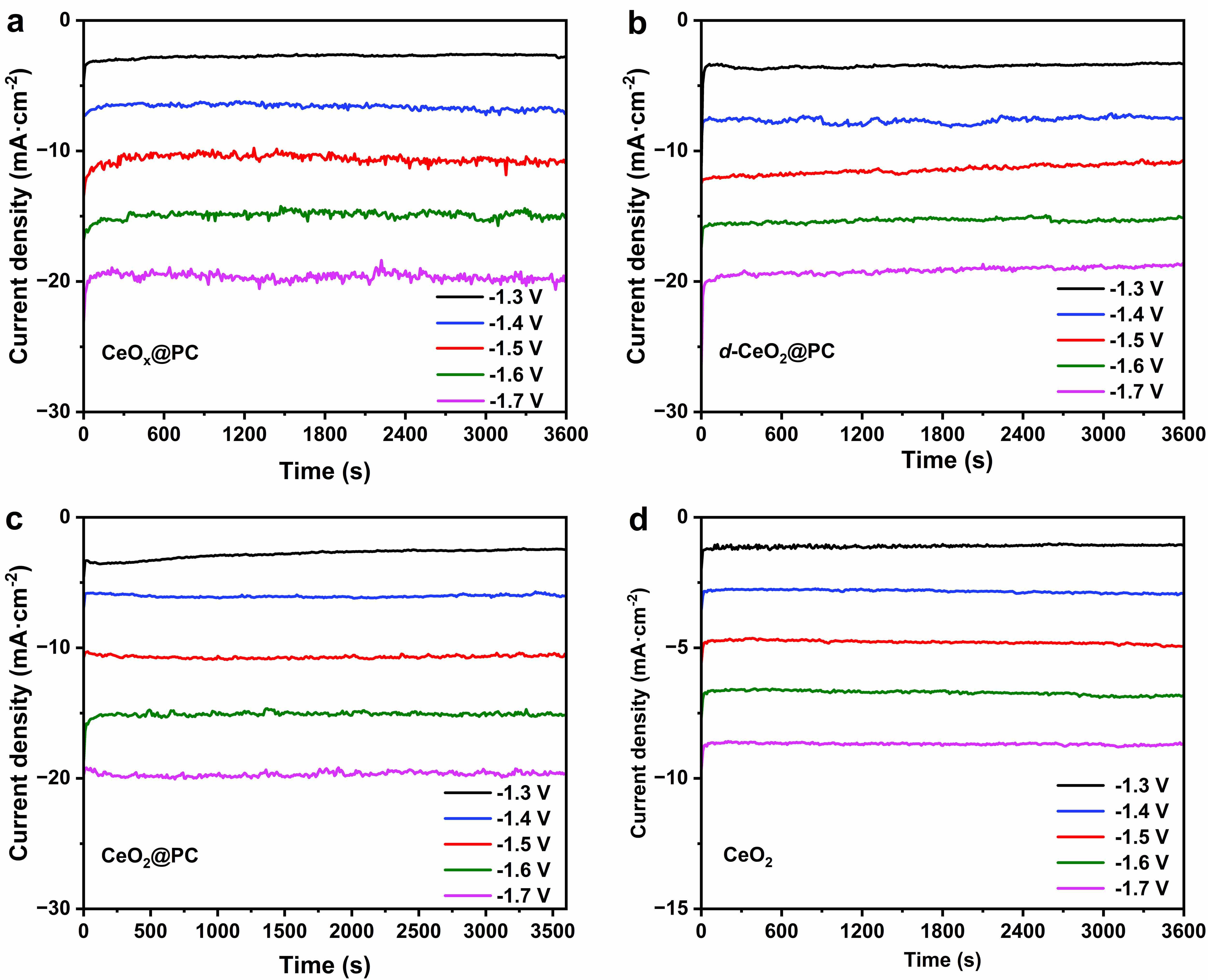
**

**Figure S12.** Chronoamperometric profiles of (a) CeO_x_/PC, (b) *d*-CeO_2_/PC, (c) CeO_2_/PC and (d) pure CeO_2_ recorded 1 h at a series of potentials ranging from −1.3 to −1.7 V vs. RHE.

**
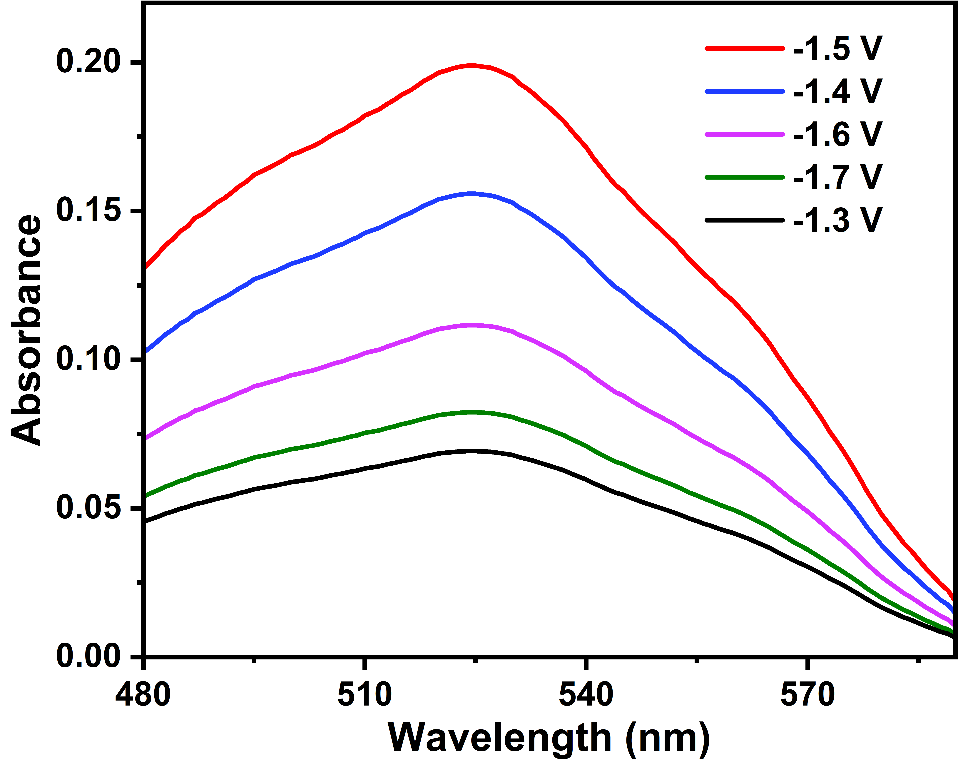
**

**Figure S13.** UV-vis spectra of the electrolyte (0.1 M KNO_3_) for *d*-CeO_x_/PC, following 1 h of electrolysis at various potentials under CO_2_ atmosphere and analyzed via the diacetyl monoxime assay.


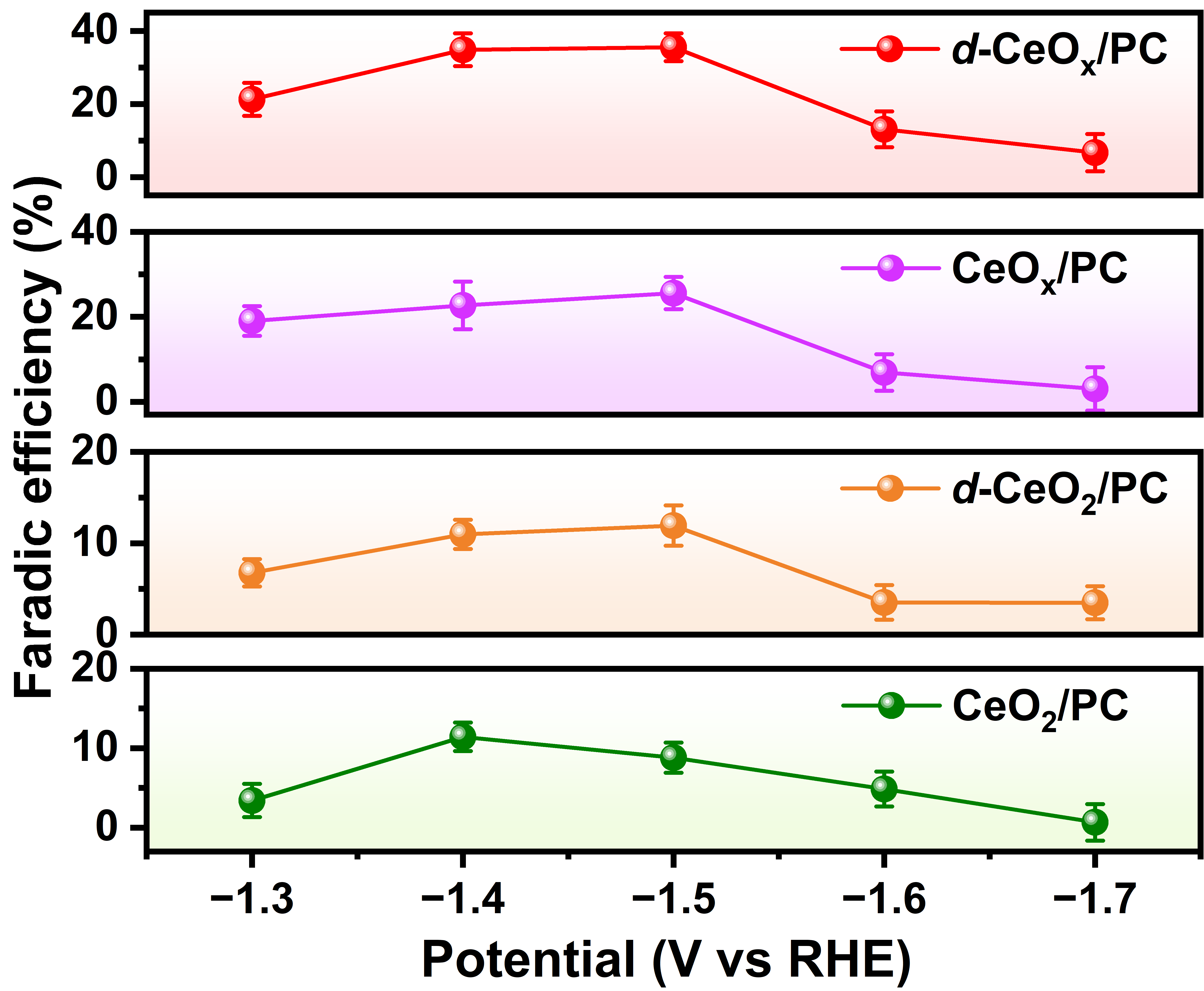


**Figure S14.** FEs of CeO_2_/PC, *d*-CeO_2_/PC, CeO_x_/PC and *d*-CeO_x_/PC at a series of potentials ranging from −1.3 to −1.7 V vs. RHE;


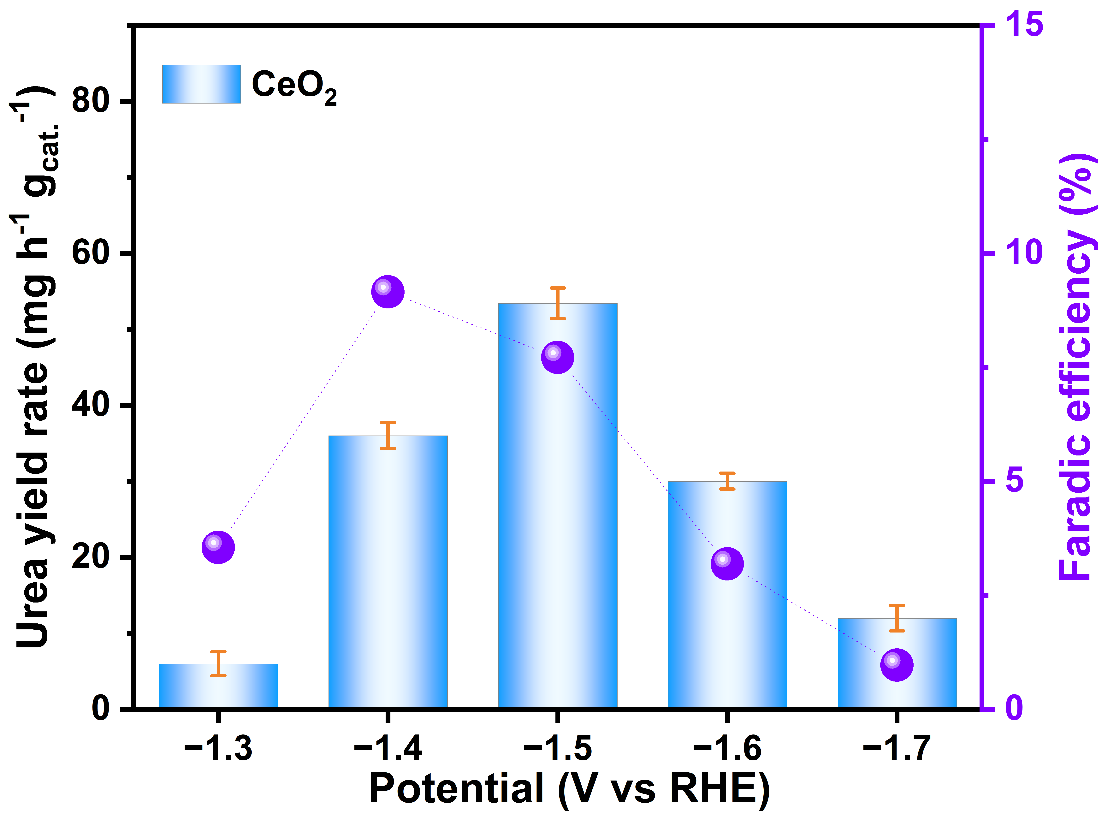


**Figure S15.** Urea yield rates of pure CeO_2_ at different potentials and the corresponding FEs.


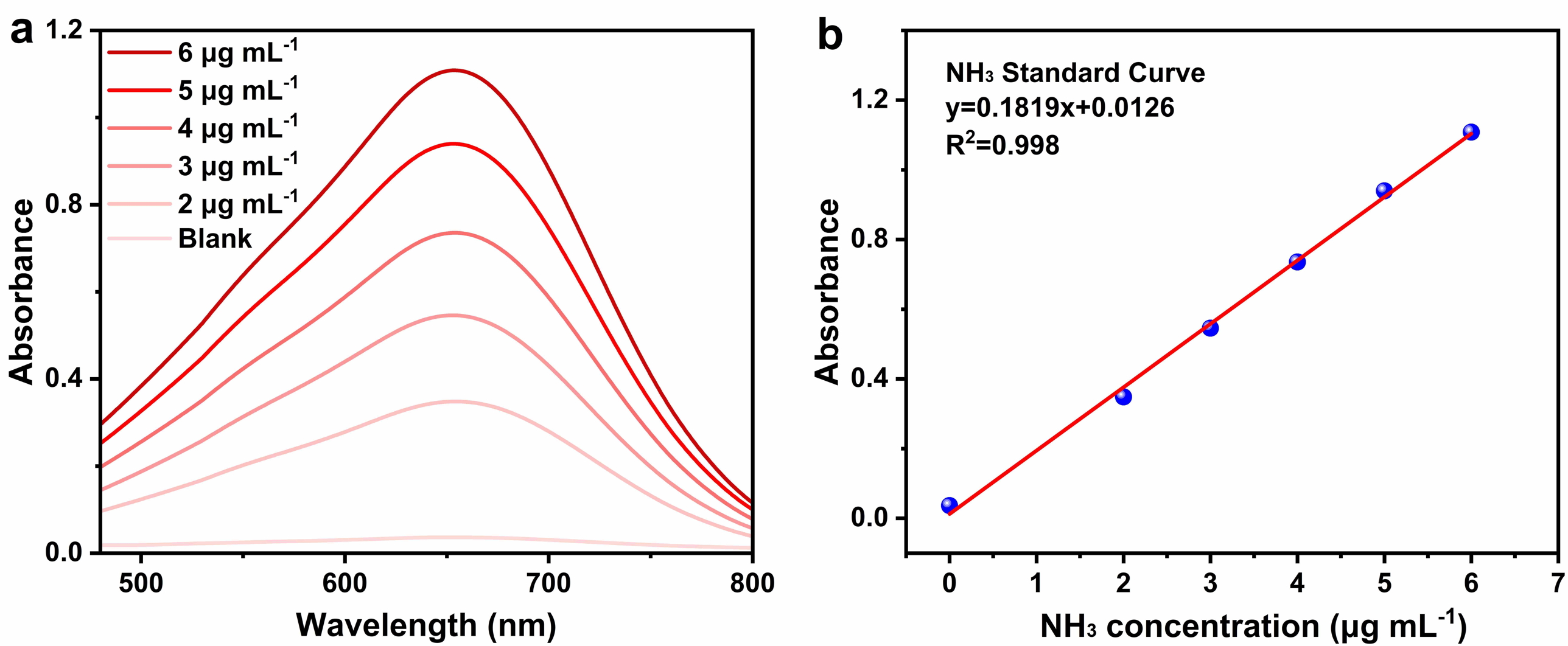


**Figure S16.** (a) UV-vis spectra from the indophenol blue assay for ammonia, following 2 h of standing at room temperature. (b) Concentration-absorbance profiles of ammonia solutions with gradient standard concentrations in 0.1 M KNO_3_. Absorbance at 655 nm was recorded using a UV-vis spectrophotometer, and the standard curve exhibits a strong linear correlation between absorbance and ammonia concentration (y = 0.1819x + 0.0126, R^2^ = 0.998).


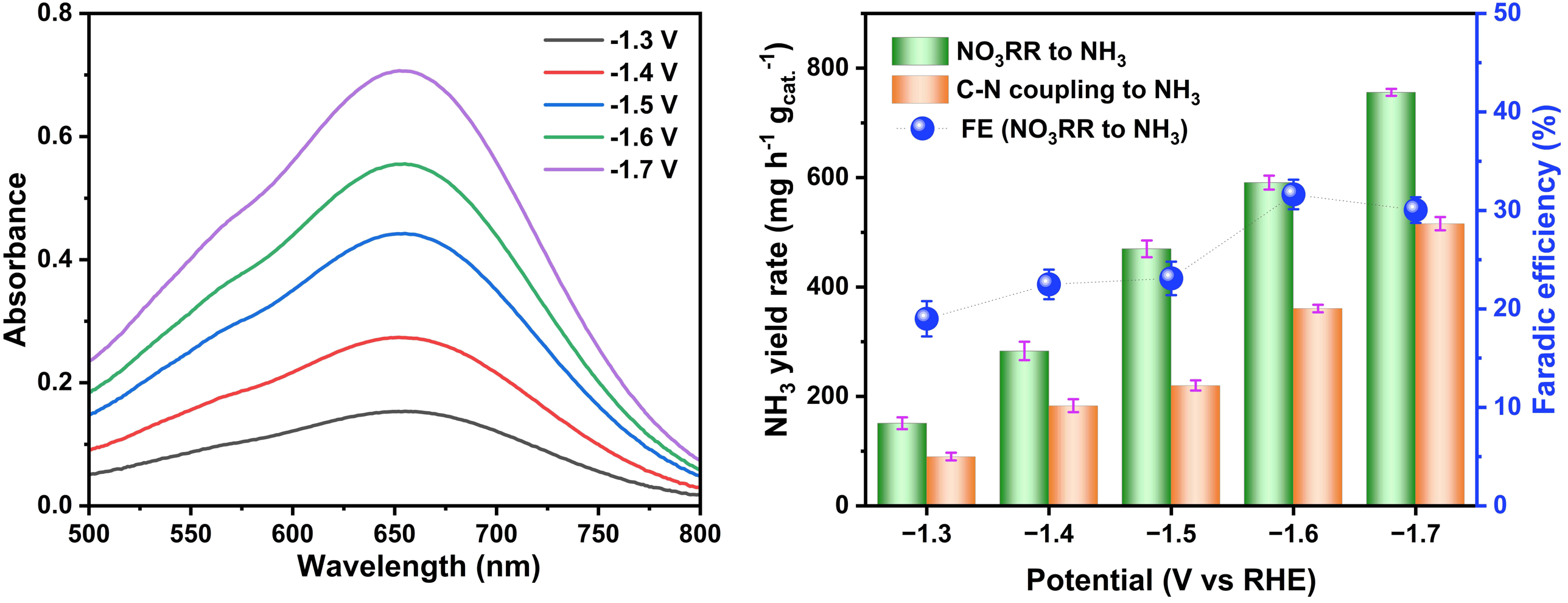


**Figure S17.** (a) UV-vis spectra and (b) ammonia production rates and the corresponding FEs of *d*-CeO_x_/PC during sole NO_3_⁻ reduction reaction and the co-electrocatalytic processes, following 1 h of electrolysis at various potentials via the indophenol blue assay.


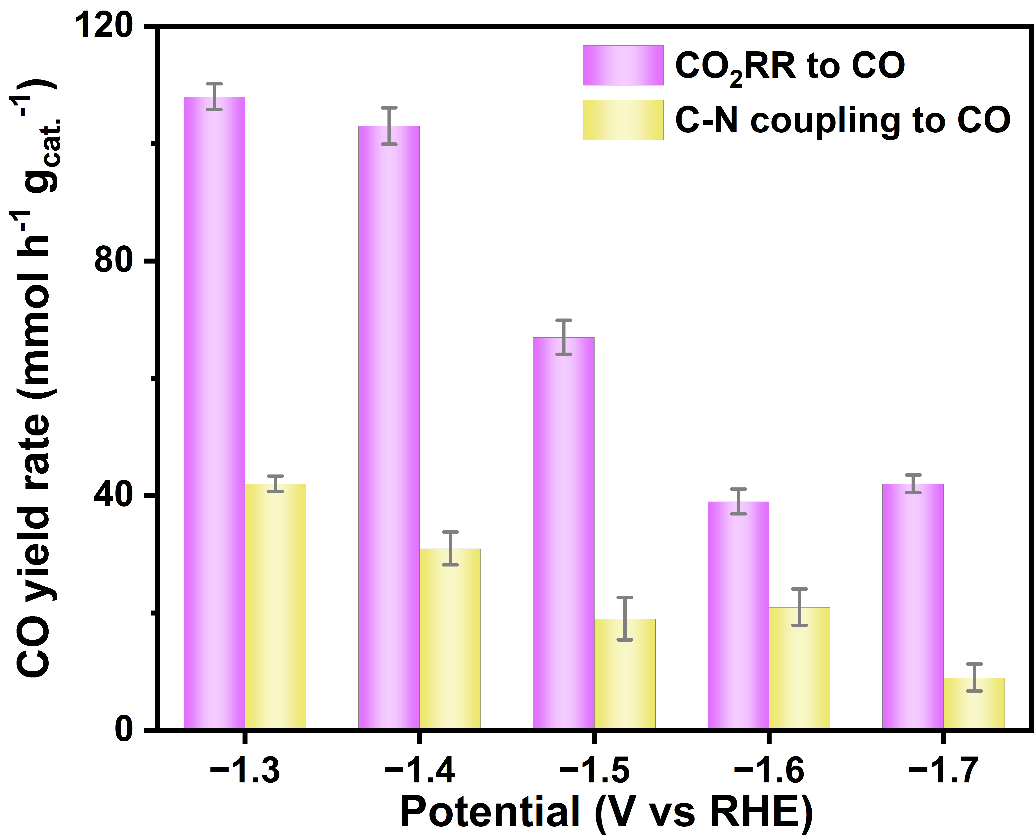


**Figure S18.** The CO yield rates of *d*-CeO_x_/PC during sole CO_2_ reduction reaction and the co-electrocatalytic processes, following 1 h of electrolysis at various potentials via gas chromatography analysis.


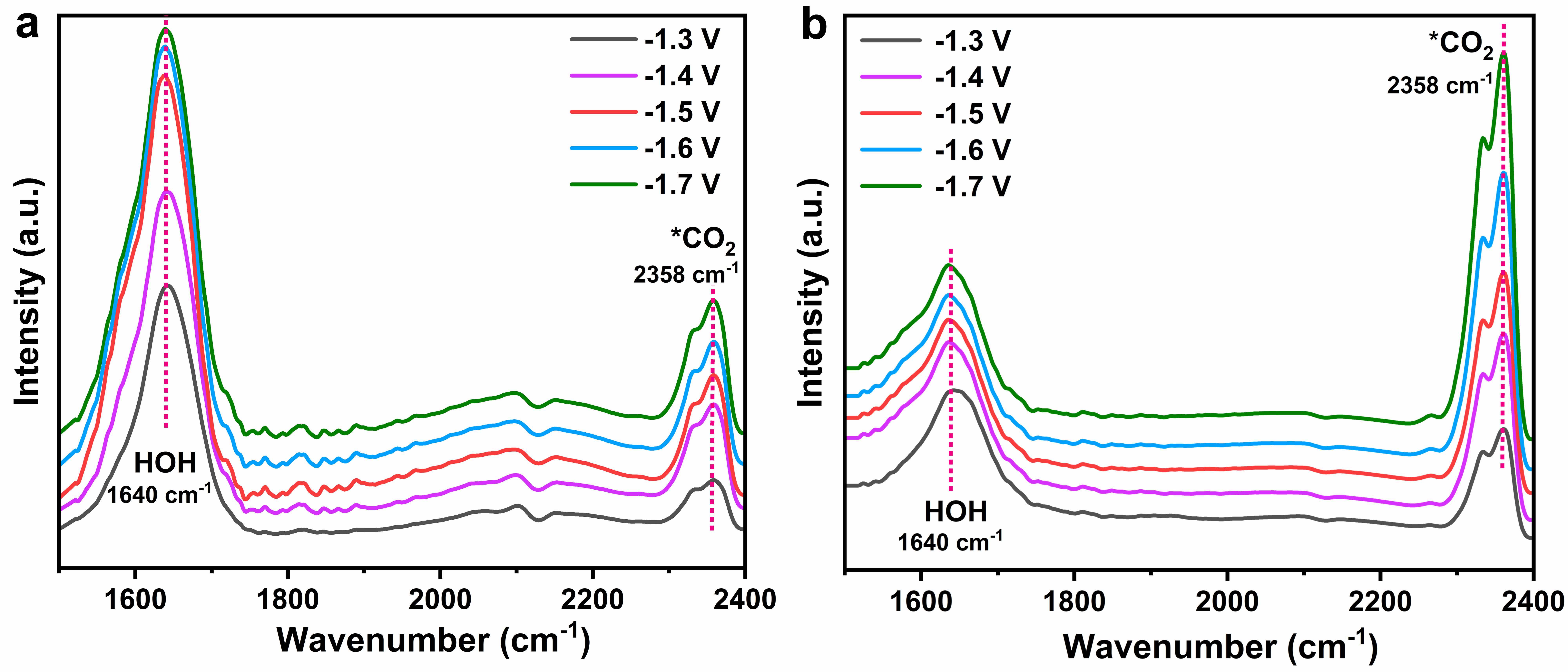


**Figure S19.** *In situ* ATR-FTIR spectroscopy of intermediate species on (a) *d*-CeO_x_/PC (1500-2400 cm^-1^), (b) CeO_2_/PC (1500-2400 cm^-1^) during electrolysis at different potentials (vs. RHE) in CO_2_-saturated 0.1 M KNO_3_ electrolyte.

.


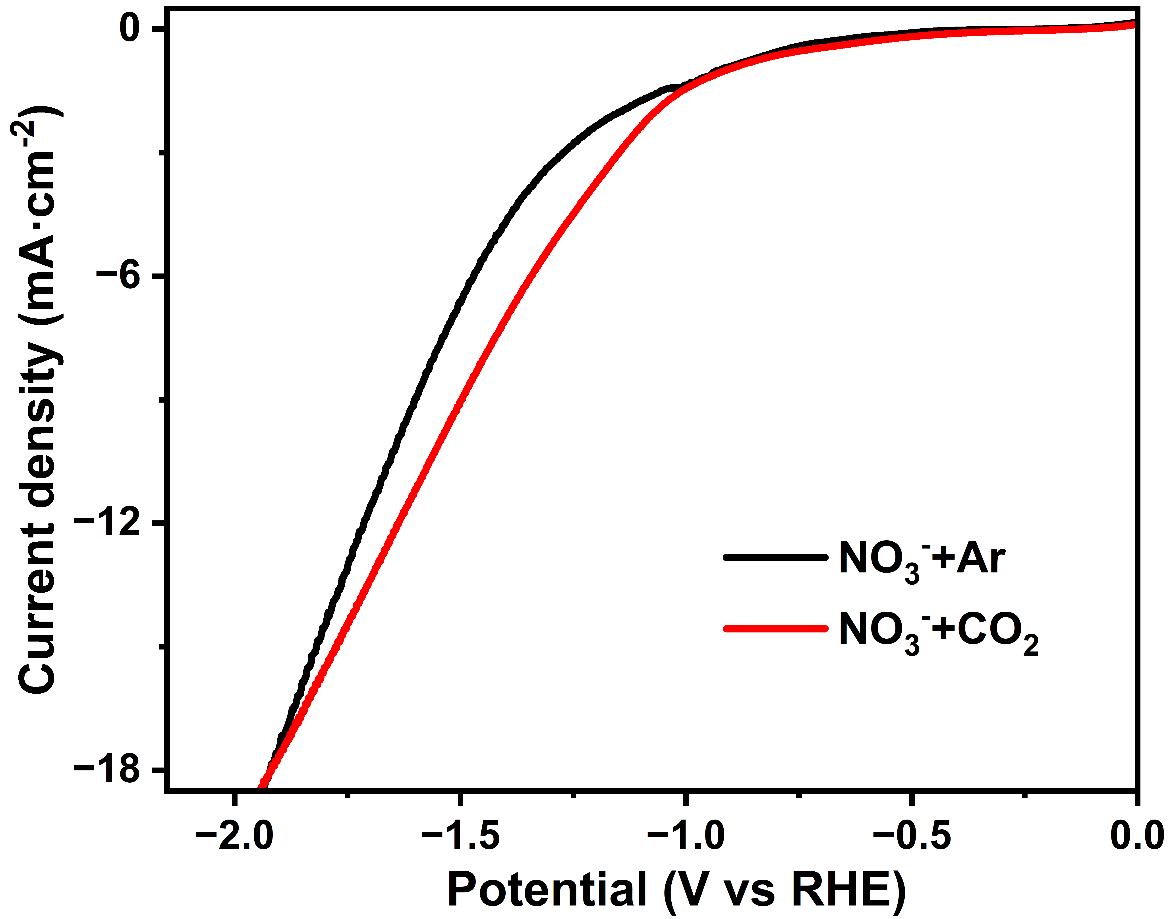


**Figure S20.** LSV curves for *d*-CeO_x_/PC in Ar or CO2 saturated 0.1 M KNO_3_ electrolyte.





**Figure S21.** Cyclic voltammogram curves of (a) *d*-CeO_x_/PC, (b) CeO_x_/PC, (c) *d*-CeO_2_/PC and (d) CeO_2_/PC.


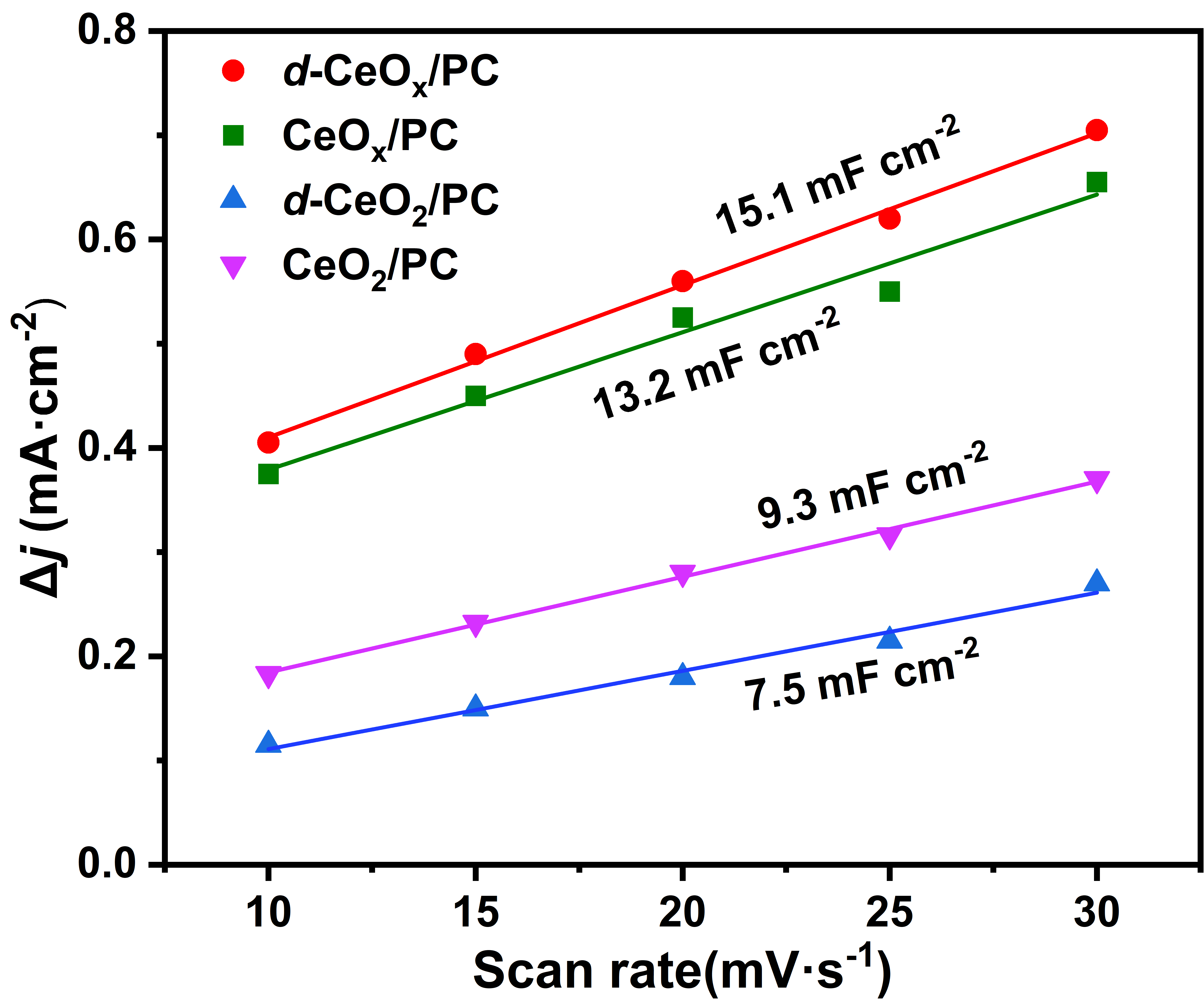


**Figure S22.** The double-layer capacitance of *d*-CeO_x_/PC, CeO_x_/PC, *d*-CeO_2_/PC, and CeO_2_/PC. (The electrochemical active surface area measurements on electrocatalysts)


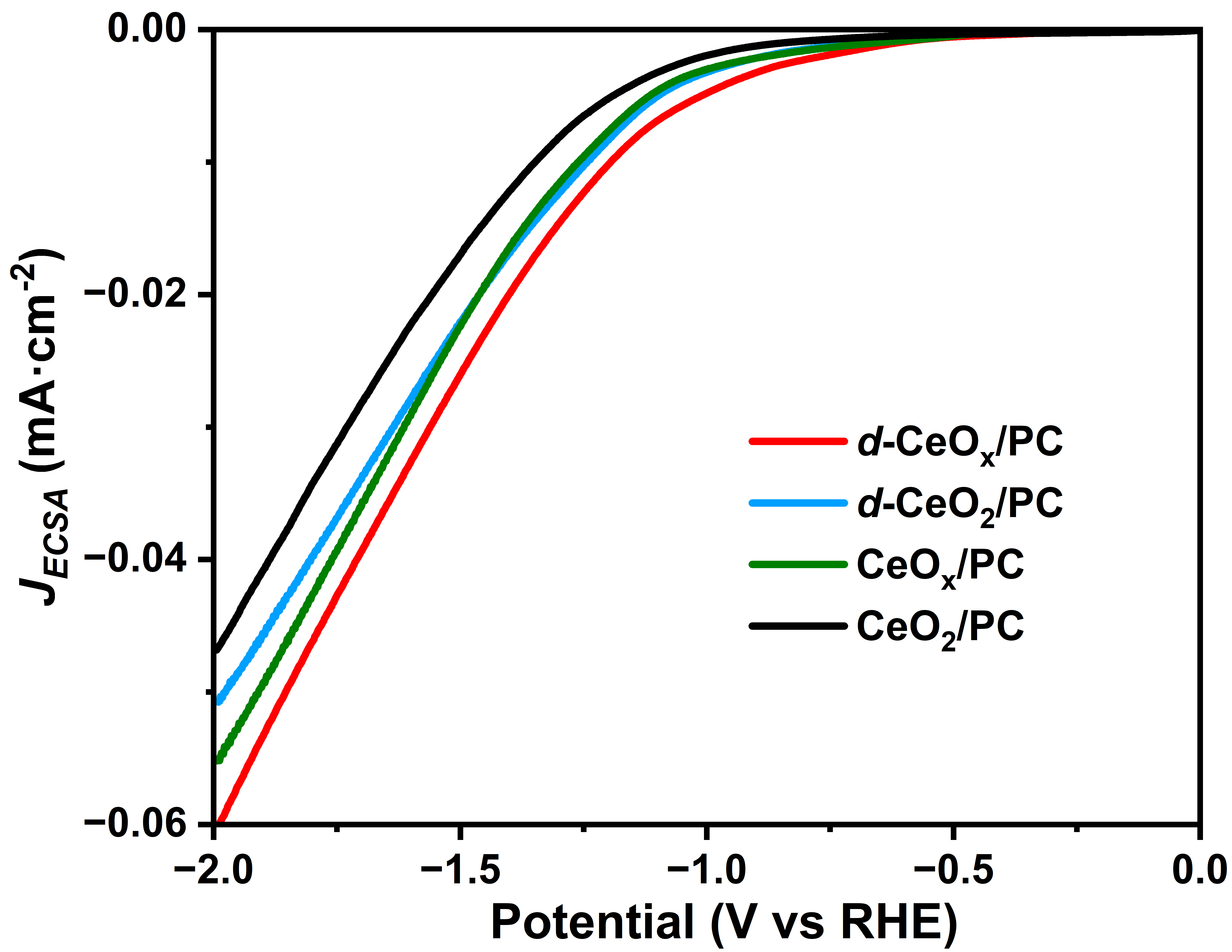


**Figure S23.** Normalized LSV of *d*-CeO_x_/PC, CeO_x_/PC, *d*-CeO_2_/PC, and CeO_2_/PC.


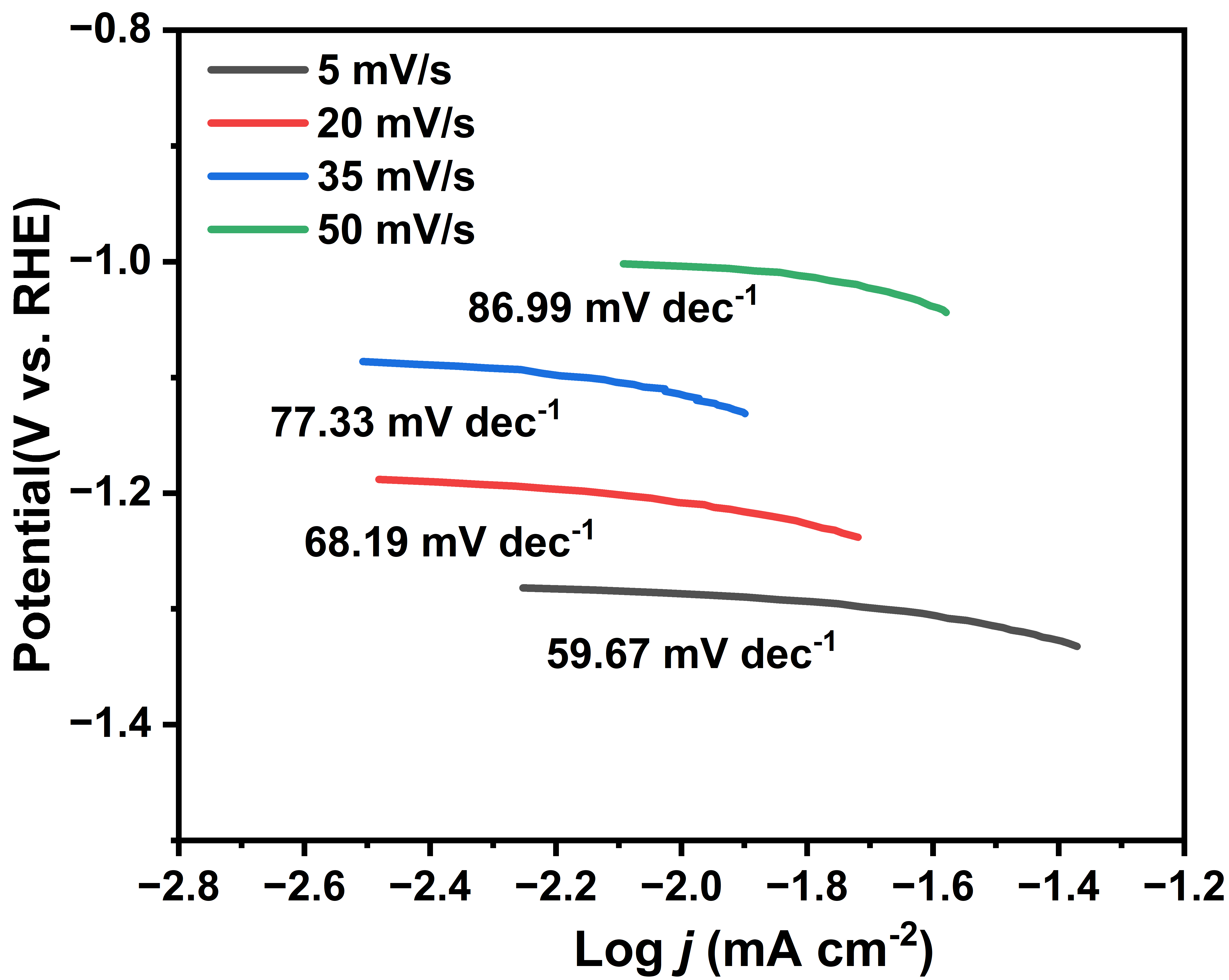


**Figure S24.** Tafel slope of *d*-CeO_x_/PC at different scan speeds.


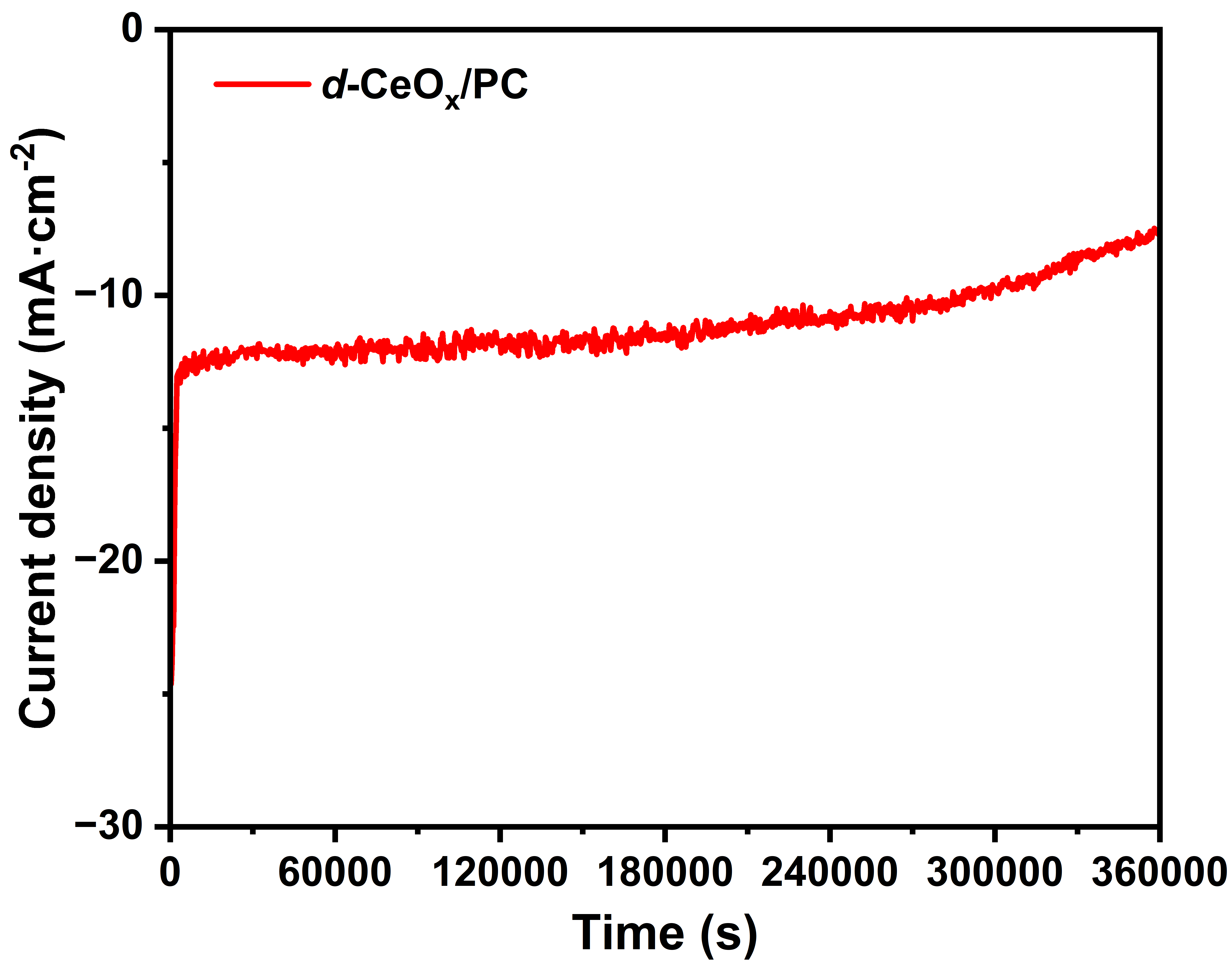


**Figure S25.** Stability test of *d*-CeO_x_/PC at -1.5 V vs. RHE for 100 h.


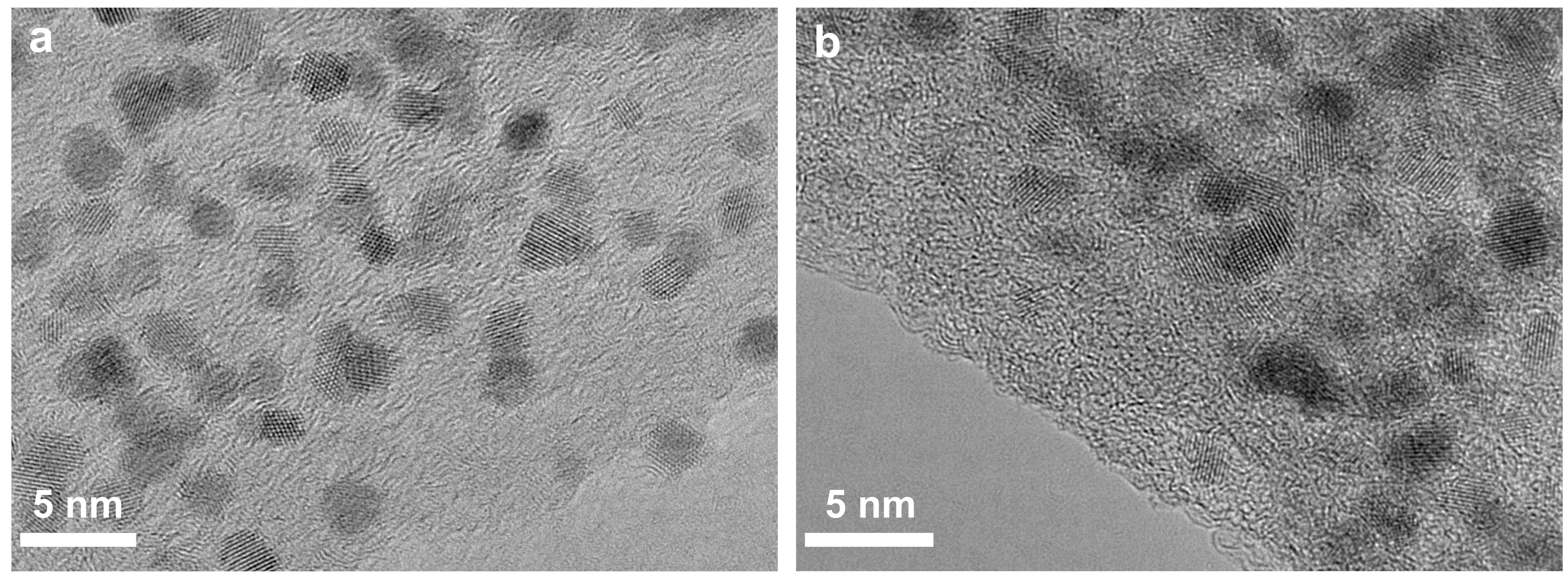


**Figure S26.** TEM image of *d*-CeO_x_/PC (a) before and (b) after the stability test of 100 h.


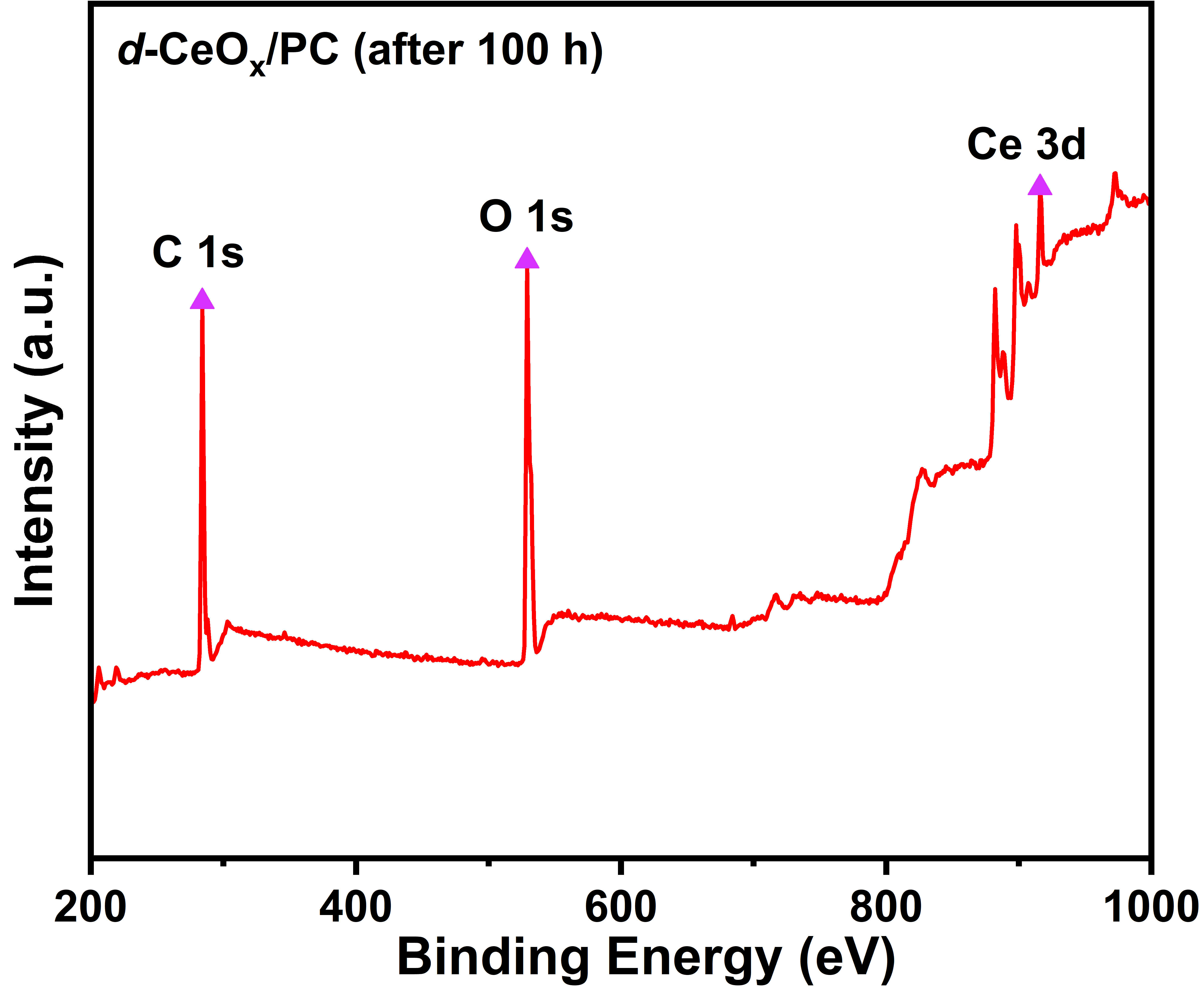


**Figure S27.** XPS survey spectrum of *d*-CeO_x_/PC after the stability test of 100 h.


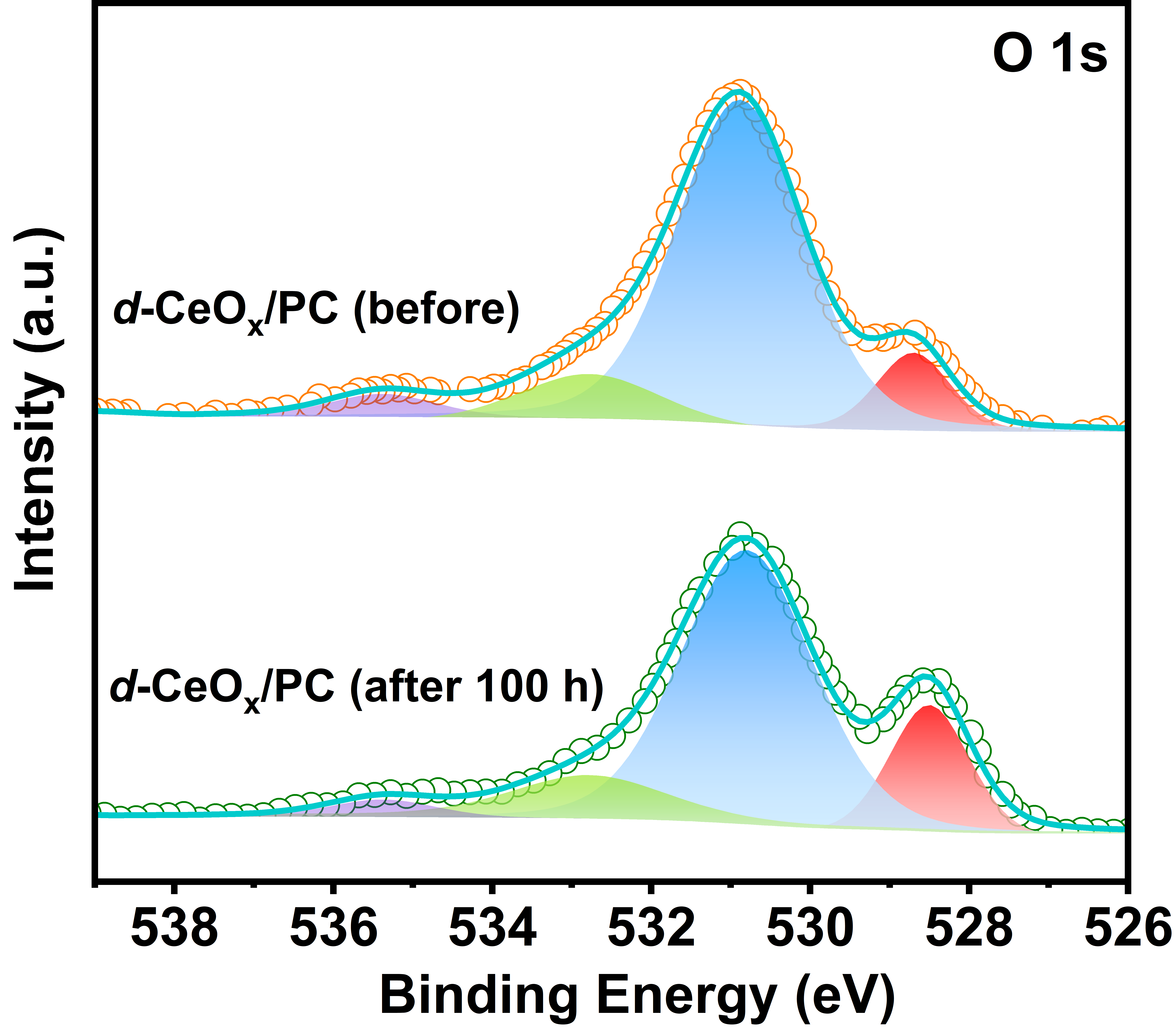


**Figure S28.** O 1s XPS spectra of *d*-CeO_x_/PC before and after the stability test of 100 h.

**
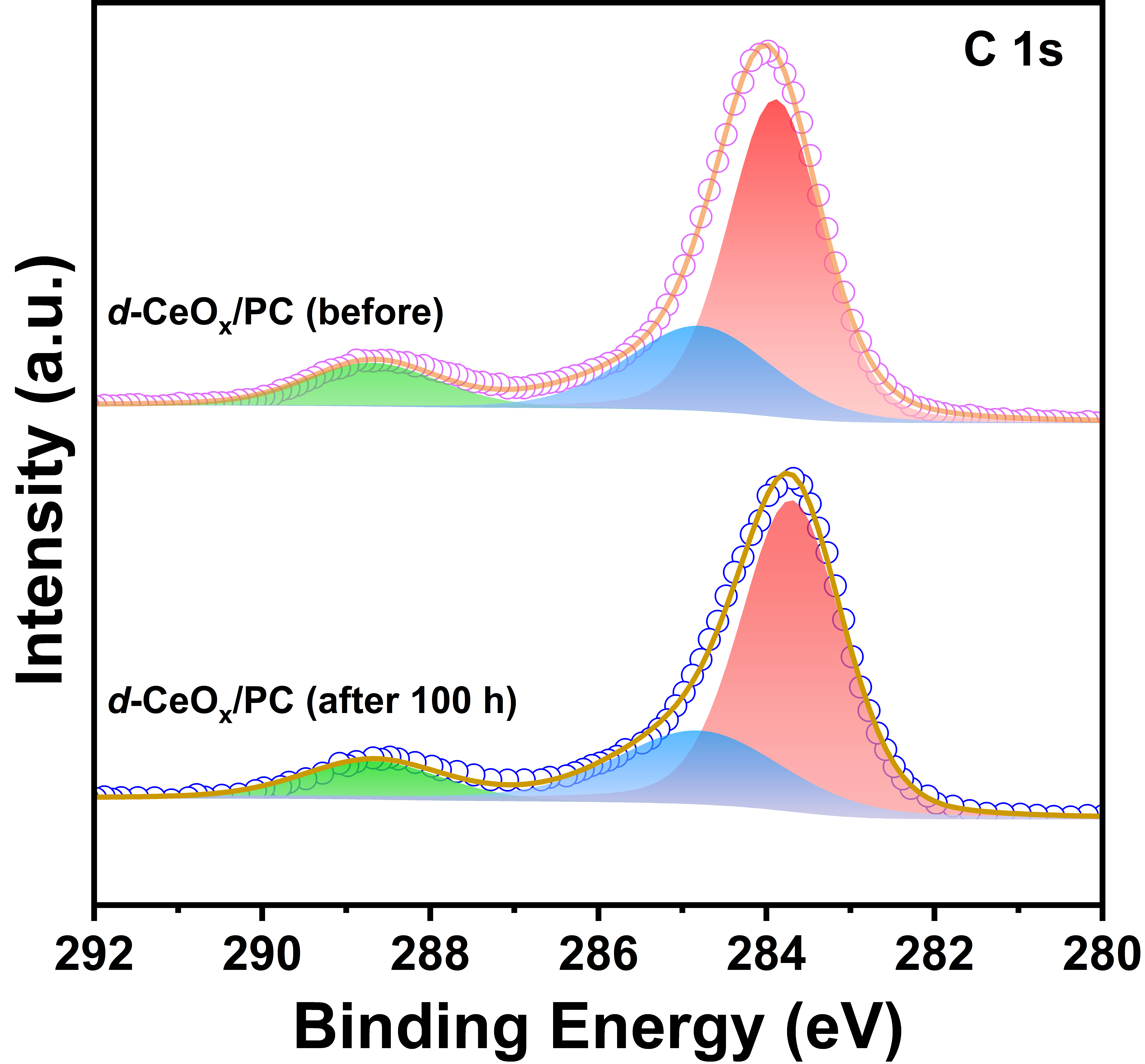
**

**Figure S29.** C 1s XPS spectra of *d*-CeO_x_/PC before and after the stability test of 100 h.


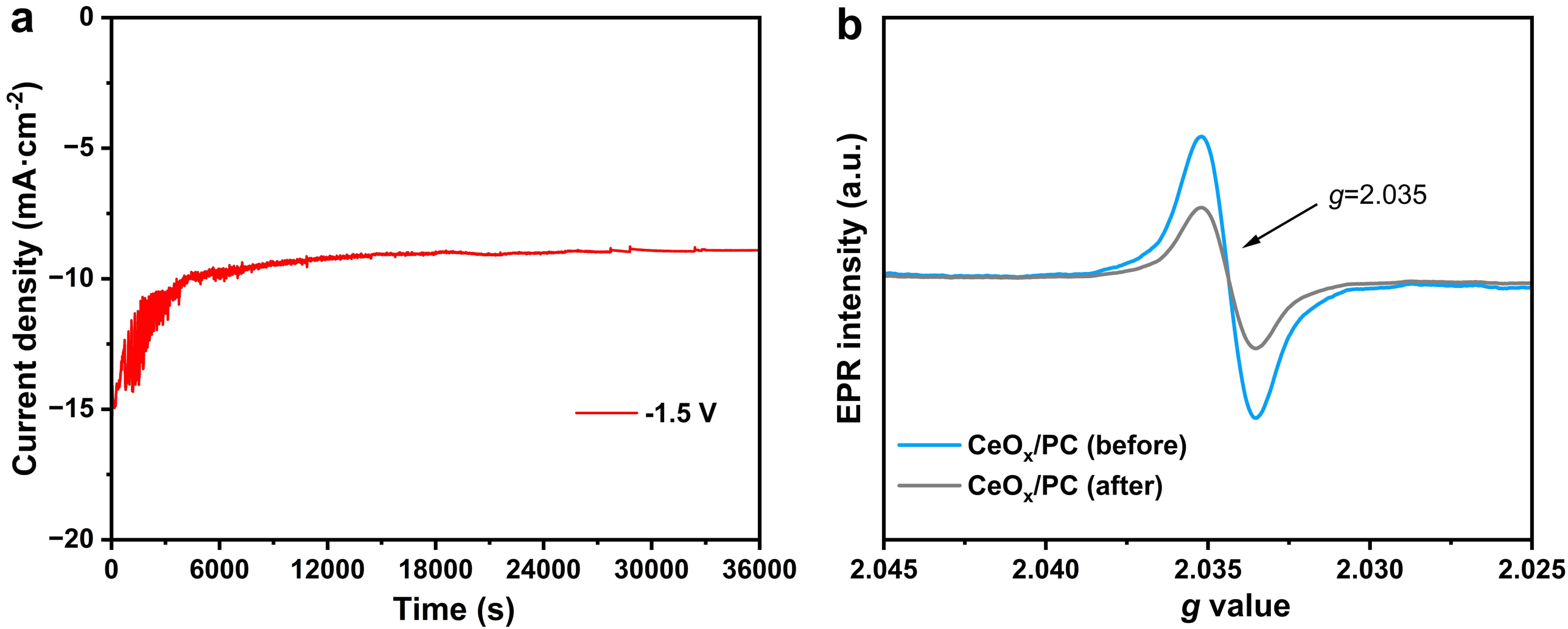


**Figure S30.** (a) Stability tests of CeO_x_/PC at −1.5 V versus RHE; (b) EPR spectra of CeO_x_/PC before and after the stability test of 10 h.


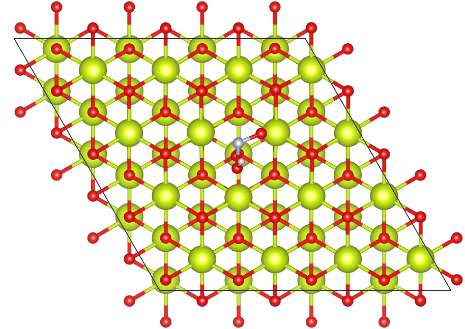

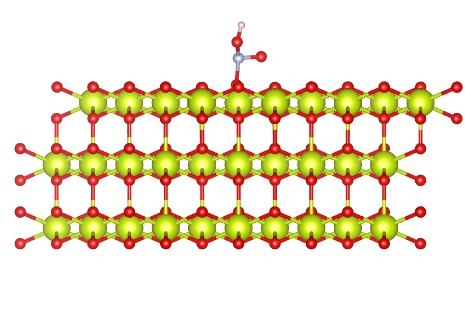


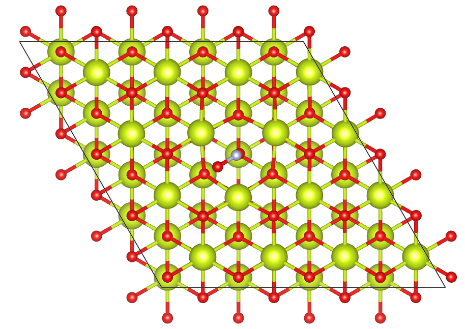

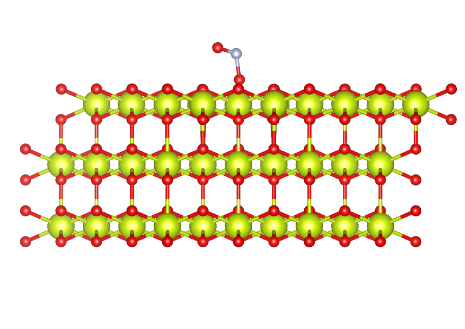


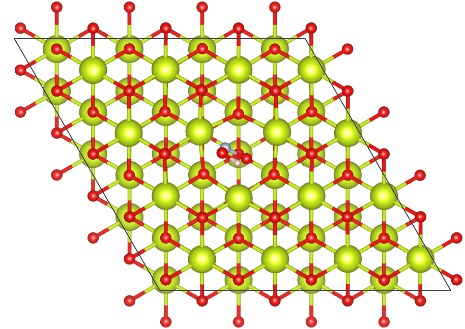

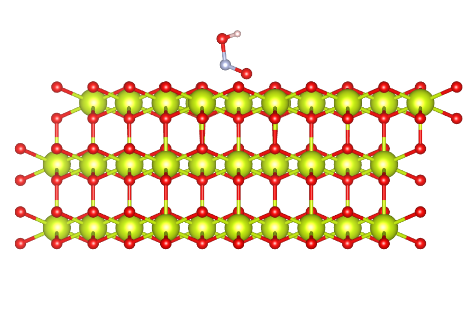


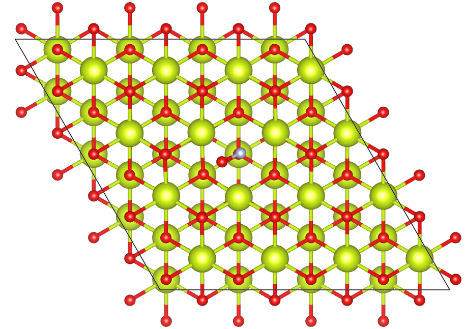

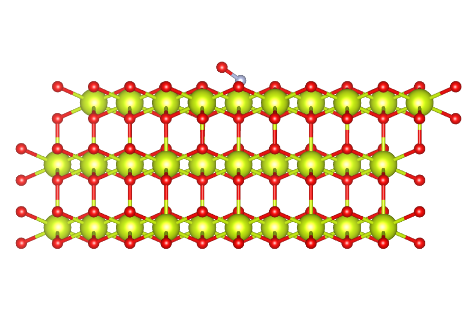


**Figure S31.** The structures of adsorption states on *d*-CeO_x_/PC (111) surface: *HNO_3_, *NO_2_, *NOOH, *NO; The yellow, red, gray, brown and pink balls represent Ce, O, N, C, and H atoms, respectively.


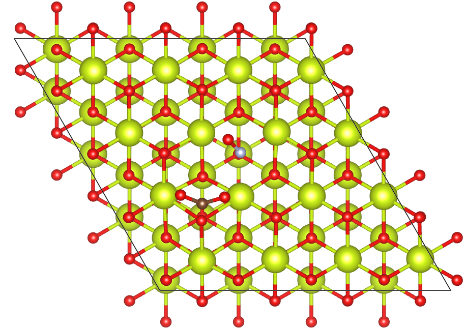

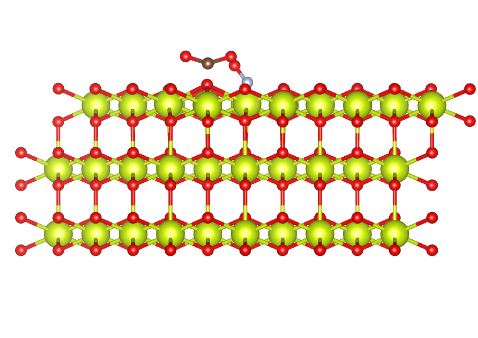


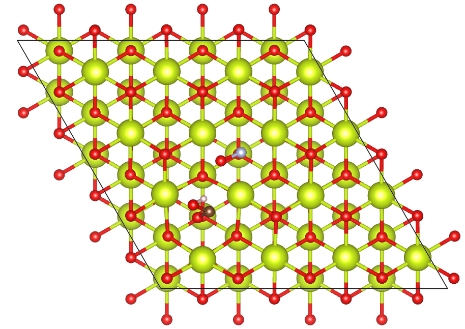

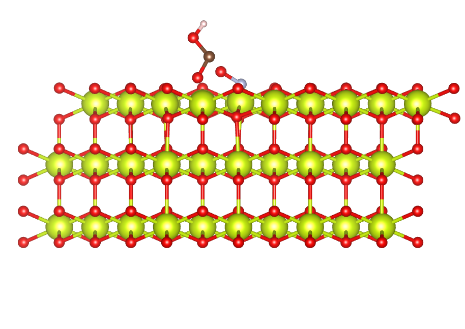


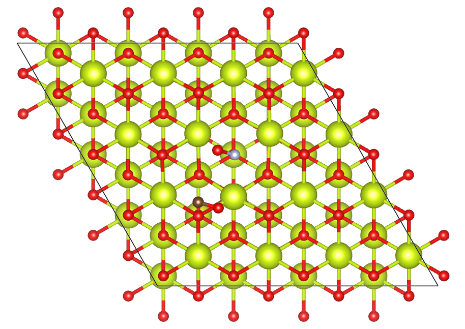

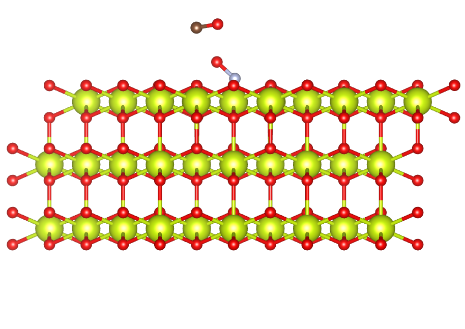


**Figure S32.**The structures of adsorption states on *d*-CeO_x_/PC (111) surface: *CO_2_, *COOH, *CO; The yellow, red, gray, brown and pink balls represent Ce, O, N, C, and H atoms, respectively.


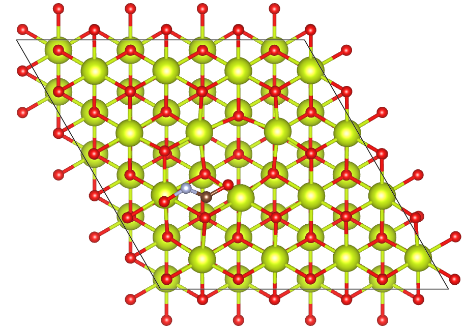

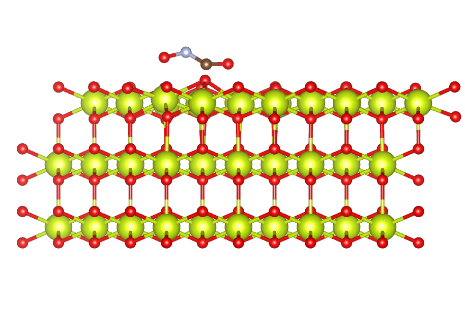


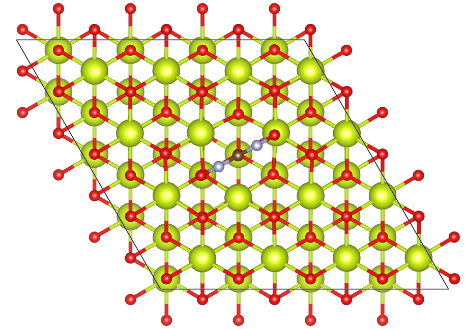

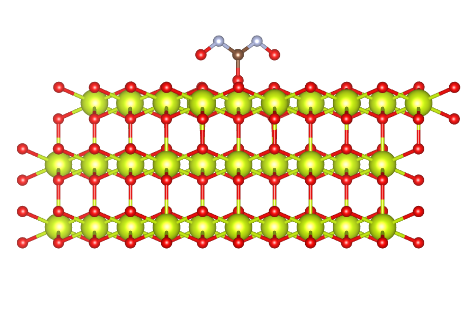


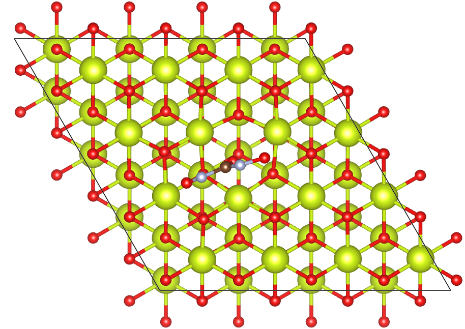

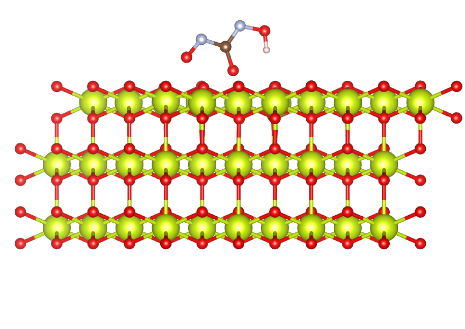


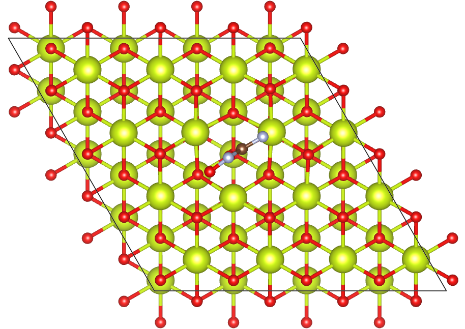

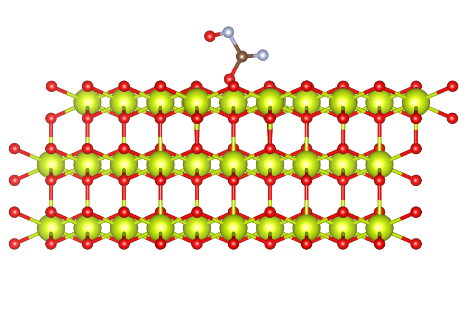


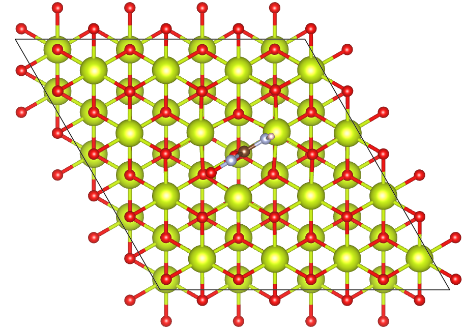

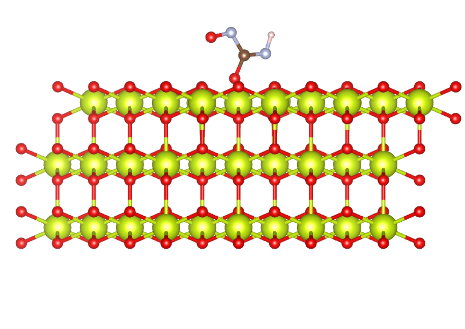


**Figure S33.** The structures of adsorption states on *d*-CeO_x_/PC (111) surface: *OCNO, *ONCONO, *ONCONOH, *ONCON, *ONCONH; The yellow, red, gray, brown and pink balls represent Ce, O, N, C, and H atoms, respectively.


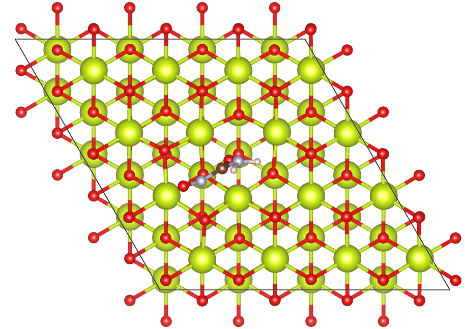

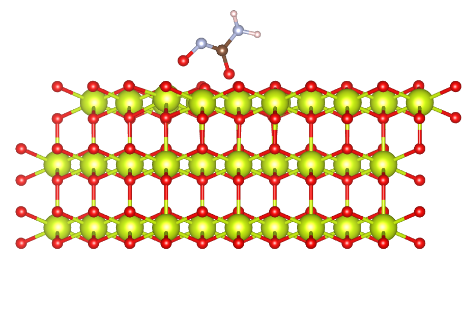


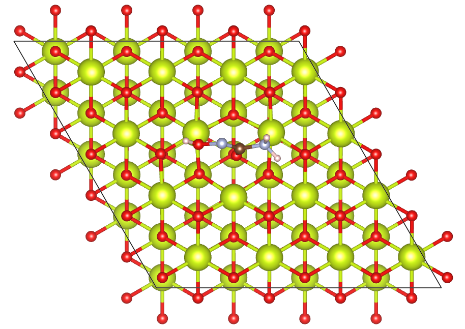

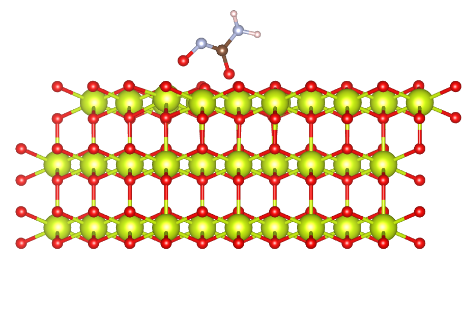


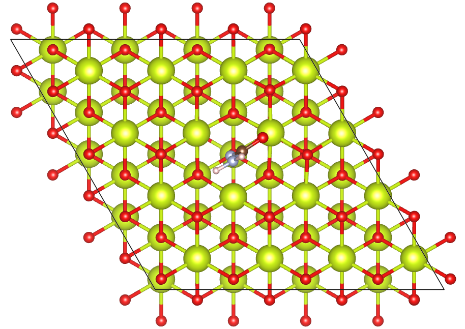

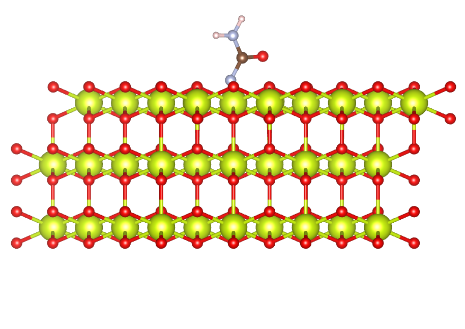


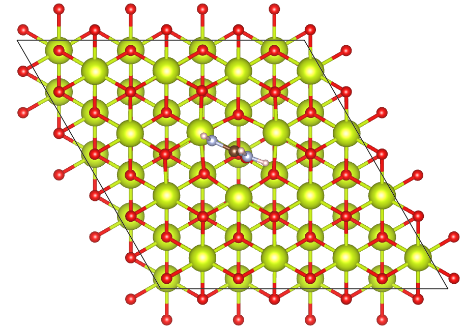

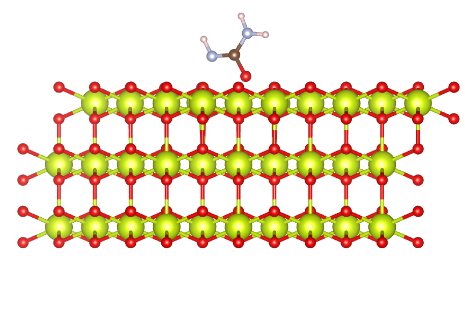


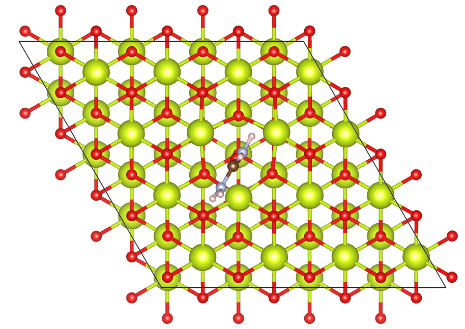

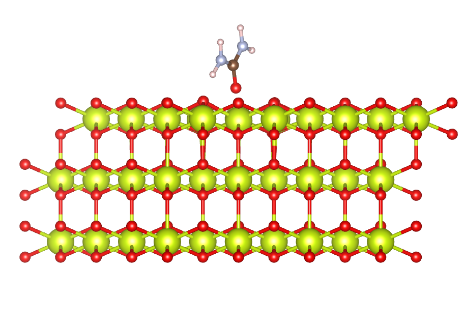


**Figure S34.**The structures of adsorption states on *d*-CeO_x_/PC (111) surface: *ONCONH_2_, *HONCONH_2_, *NCONH_2_, *HNCONH_2_, *CO(NH_2_)_2_. The yellow, red, gray, brown and pink balls represent Ce, O, N, C, and H atoms, respectively.

**Table S1** lattice cell parameter of *d*-CeO_x_/PC, CeO_x_/PC, *d*-CeO_2_/PC and CeO_2_/PC.

| Electrocatalyst | a | b | c | lattice volume |
| --- | --- | --- | --- | --- |
| *d*-CeO_x_/PC | 5.23 Å | 5.25 Å | 5.25 Å | 144.15 Å^3^ |
| CeO_x_/PC | 5.38 Å | 5.40 Å | 5.40 Å | 156.88 Å^3^ |
| *d*-CeO_2_/PC | 5.25 Å | 5.30 Å | 5.30 Å | 147.47 Å^3^ |
| CeO_2_/PC | 5.40 Å | 5.40 Å | 5.40 Å | 157.46 Å^3^ |

**Table S2.** Local structure parameters around Ce in samples estimated by EXAFS analysis.

| Catalyst | Shell | *N^a^* | *R^b^ (Å)* | *σ^2c^(Å^2^)* | *ΔE_0_^d^ (eV)* |
| --- | --- | --- | --- | --- | --- |
| CeO_2_/PC | Ce-O | 8.02 | 2.31 | 0.0098 | 3.96 |
| d-CeO_x_/PC | Ce-O | 7.52 | 2.29 | 0.0096 | 3.15 |

^a^ Coordination numbers. ^b^ Bond distance. ^c^ Debye-Waller factors. ^d^ Inner potential correction. ^e^ Goodness of fit. *Ѕ*_0_^2^ was set to 0.97. The data range used for data fitting in k-space (∆k) and R-space (∆R) are 1-7.8 Å^-1^ and 1.05-2.4 Å, respectively.

**Table S3.** ICP-OES analysis of the Ce content in CeO_2_/PC and *d*-CeO_x_/PC catalysts before and after the reaction in a 0.1 M KNO_3_ + CO_2_-saturated electrolyte using chronoamperometry.

| **Period** | **Ce content in CeO_2_/PC** | **Ce content in d-CeO_x_/PC** |
| --- | --- | --- |
| **0 h** | 21.25% | 25.12% |
| **10 h** | 21.23% | 25.11% |
| **100 h** | **/** | 25.06% |

**Table S4.** Comparison of the electrocatalytic performance of *d*-CeO_x_/PC versus urea synthesis with other electrocatalysts reported in literatrues.

| Catalyst | Reactant | Urea yield rate | FE | Stability | Current density | Ref. |
| --- | --- | --- | --- | --- | --- | --- |
| *d*-CeO_x_/PC | CO_2_/NO_3_⁻ | 532.13 mg h^-1^ g_cat._^-1^ | 35.51% | 100 h | -13 mA cm^-2^ | / |
| NiPd_100-x_Cu_x_/ZnO | CO_2_/NO_3_⁻ | 194.02 mg h^-1^ g_cat._^-1^ | 29.38% | 12 h | -4 mA cm^-2^ | [21] |
| Cu-PMOF | CO_2_/NO_3_⁻ | 25.5 μmol h^-1^ mg_cat._^-1^ | 52.7% | 36 h | -2.5 mA cm^-2^ | [22] |
| VB_12_-CNTs | CO_2_/NO_3_⁻ | 164.04 μg h^-1^ mg_cat._^-1^ | 26.04% | 50 h | -1.5 mA cm^-2^ | [23] |
| Bi: 10 %In/C NPs | CO_2_/NO_3_⁻ | 606.38 μg h^-1^ mg_cat._^-1^ | 20.31% | 14 h | -4 mA cm^-2^ | [24] |
| MnPc-AFG | CO_2_/NO_3_⁻ | 397.61 mg h^-1^ g_cat._^-1^ | 27.06% | 10 h | -5 mA cm^-2^ | [25] |
| a-SnBi NS/rGO | CO_2_/NO_3_⁻ | 462.37 μg h^-1^ mg_cat._^-1^ | 78.36 | 50 h | -0.7 mA cm^-2^ | [26] |
| NC | CO_2_/NO_3_⁻ | 596.1μg h^-1^ mg_cat._^-1^ | 62% | 7.5 h | -0.87 mA cm^-2^ | [27] |

**References**

[1] X. Wei, X. Wen, Y. Liu, et al., “Oxygen Vacancy-Mediated Selective C-N Coupling toward Electrocatalytic Urea Synthesis,” *Journal of the American Chemical Society* 144 (2022): 11530.

https://doi.org/10.1021/jacs.2c03452

[2] X. Liu, P. V. Kumar, Q. Chen, et al., “Carbon nanotubes with fluorine-rich surface as metal-free electrocatalyst for effective synthesis of urea from nitrate and CO_2_,” *Applied Catalysis B-Environment and Energy* 316 (2022): 121618.

https://doi.org/10.1016/j.apcatb.2022.121618

[3] J. Geng, S. Ji, M. Jin, et al., “Ambient Electrosynthesis of Urea with Nitrate and Carbon Dioxide over Iron-Based Dual-Sites,” *Angewandte Chemie International Edition* 62 (2023): e202210958.

https://doi.org/10.1002/anie.202210958

[4] X. Zhang, X. Zhu, S. Bo, et al., “Identifying and tailoring C-N coupling site for efficient urea synthesis over diatomic Fe-Ni catalyst,” *Nature Communications* 13 (2022): 5337.

https://doi.org/10.1038/s41467-022-33066-6

[5] X. Wei, Y. Liu, X. Zhu, et al., “Dynamic Reconstitution Between Copper Single Atoms and Clusters for Electrocatalytic Urea Synthesis,” *Advanced Materials* 35 (2023): 2300020.

https://doi.org/10.1002/adma.202300020

[6] Q. Zhao, X. Lu, Y. Wang, et al., “Sustainable and High-Rate Electrosynthesis of Nitrogen Fertilizer,” *Angewandte Chemie International Edition*62 (2023): 202307123.

https://doi.org/10.1002/anie.202307123

[7] Q. Zhao, Y. Liu, Y. Zhang, et al., “Activity‐Selectivity Trends in Electrochemical Urea Synthesis: Co-Reduction of CO_2_ and Nitrates Over Single‐Site Catalysts,” *Advanced Science* 12 (2025): 2501882.

https://doi.org/10.1002/advs.202501882

[8] Y. Yoon, B. Yan, Y. Surendranath, “Suppressing ion transfer enables versatile measurements of electrochemical surface area for intrinsic activity comparisons,” *Journal of the American Chemical Society* 140 (2018): 2397.

https://doi.org/10.1021/jacs.7b10966

[9] S. Jung, C. C. McCrory, I. M. Ferrer, J. C. Peters, T. F. Jaramillo, “Benchmarking nanoparticulate metal oxide electrocatalysts for the alkaline water oxidation reaction,” *Journal of Materials Chemistry A* 4 (2016): 3068.

https://doi.org/10.1039/C5TA08468G

[10] R. Kötz, M. Carlen, “Principles and applications of electrochemical capacitors,” *Electrochimica Acta* 45 (2000): 2483.

https://doi.org/10.1016/S0013-4686(00)00354-6

[11] Z. Zhao, H. Yang, Y. Zhu, S. Luo, J. Ma, “Interfacial N-Cu-S coordination mode of CuSCN/C_3_N_4_ with enhanced electrocatalytic activity for hydrogen evolution,” *Nanoscale* 11 (2019): 12938.

https://doi.org/10.1039/c9nr02860a

[12] G. Kresse, J. Hafner, “Norm-conserving and ultrasoft pseudopotentials for first-row and transition elements,” *Journal of Physics: Condensed Matter* 6 (1994): 8245.

https://doi.org/10.1088/0953-8984/6/40/015

[13] G. Kresse, J. Furthmüller, “Efficient iterative schemes for ab initio total-energy calculations using a plane-wave basis set,” *Physical Review B* 54 (1996): 11169.

https://doi.org/10.1103/PhysRevB.54.11169

[14] P. E. Blochl, “Projector augmented-wave method,” *Journal of Physics: Condensed Matter* 50 (1994): 17953.

https://doi.org/10.1103/physrevb.50.17953

[15] G. Kresse, D. Joubert, “From ultrasoft pseudopotentials to the projector augmented-wave method,” *Physical Review B* 59 (1999): 1758.

https://doi.org/10.1103/PhysRevB.59.1758

[16] J. P. Perdew, K. Burke, M. Ernzerhof, “Generalized Gradient Approximation Made Simple,” *Physical Review Letters* 77 (1996): 3865.

https://doi.org/10.1103/PhysRevLett.77.3865

[17] J. K. Nørskov, J. Rossmeisl, A. Logadottir, et al., “Origin of the Overpotential for Oxygen Reduction at a Fuel-Cell Cathode,” *Journal of Physical Chemistry B* 108 (2004): 17886.

https://doi.org/10.1021/jp047349j

[18] J. K. Nørskov, T. Bligaard, A. Logadottir, et al., “Trends in the Exchange Current for Hydrogen Evolution,” *Journal of the Electrochemical Society* 152 (2005): J23.

https://doi.org/10.1149/1.1856988

[19] A. A. Peterson, F. Abild-Pedersen, F. Studt, J. Rossmeisl, J. K. Nørskov, “How copper catalyzes the electroreduction of carbon dioxide into hydrocarbon fuels,” *Energy & Environmental Science* 3 (2010): 1311.

https://doi.org/10.1039/C0EE00071J

[20] J. H. Montoya, C. Tsai, A. Vojvodic, J. K. Nørskov, “The Challenge of Electrochemical Ammonia Synthesis: A New Perspective on the Role of Nitrogen Scaling Relations,” *ChemSusChem* 8 (2015): 2180.

https://doi.org/10.1002/cssc.201500322

[21] K. Li, Z. Li, J. Guo, G. Zhao, J. Liu, H. Xu, “Electrocatalytic Synthesis of Urea from Carbon Dioxide and Nitrate over ZnO-Based Supported Palladium-Copper Alloy Catalysts,” *Industrial & Engineering Chemistry Research* 64 (2025): 14841.

https://doi.org/10.1021/acs.iecr.5c01792

[22] Y. Tan, X. Chen, J. Yuan, G. Sheng, W. Deng, H. Wu, “Concentration-Adaptive Electrocatalytic Urea Synthesis From CO_2_ and Nitrate via Porphyrin and Metalloporphyrin MOFs,” *Angewandte Chemie International Edition* 137 (2025): e202513441.

https://doi.org/10.1002/anie.202513441

[23] M. Cong, Q. Liu, D. Wang, et al., “Electrocatalytic urea synthesis from CO_2_ and nitrate co-reduction on natural vitamin B12 coupled carbon nanotubes,”  *Applied Catalysis B-Environment and Energy* 351 (2024): 123941.

https://doi.org/10.1016/j.apcatb.2024.123941

[24] Y. Mao, Y. Jiang, Q. Gou, et al., “Indium-activated bismuth-based catalysts for efficient electrocatalytic synthesis of urea,” *Applied Catalysis B-Environment and Energy* 340 (2024): 123189.

https://doi.org/10.1016/j.apcatb.2023.123189

[25] A. Adalder, K. Mitra, N. Barman, et al., “Electrochemical Synthesis of Urea-Ammonium-Nitrate (UAN) Fertilizer via Dual Reduction of CO_2_ and Nitrate,” *Small* 21 (2025): 2505313.

https://doi.org/10.1002/smll.202505313

[26] X. Chen, S. Lv, H. Gu, et al., “Amorphous bismuth-tin oxide nanosheets with optimized C-N coupling for efficient urea synthesis,” *Journal of the American Chemical Society* 146 (2024): 13527.

https://doi.org/10.1021/jacs.4c03156

[27] Y. Li, S. Zheng, H. Liu, et al., “Sequential co-reduction of nitrate and carbon dioxide enables selective urea electrosynthesis,” *Nature Communications* 15 (2024): 176.

https://doi.org/10.1038/s41467-023-44131-z
